# Phenotype-tissue expression and exploration (PTEE) resource facilitates the choice of tissue for RNA-seq-based clinical genetics studies

**DOI:** 10.1186/s12864-021-08125-9

**Published:** 2021-11-07

**Authors:** Akhil Velluva, Maximillian Radtke, Susanne Horn, Bernt Popp, Konrad Platzer, Erind Gjermeni, Chen-Ching Lin, Johannes R. Lemke, Antje Garten, Torsten Schöneberg, Matthias Blüher, Rami Abou Jamra, Diana Le Duc

**Affiliations:** 1 Department of Evolutionary Genetics, Max Planck Institute for Evolutionary Anthropology, 04103 Leipzig, Germany; 2 Rudolf Schönheimer Institute of Biochemistry, Medical Faculty, University of Leipzig, Johannisallee 30, 04103 Leipzig, Germany; 3 Institute of Human Genetics, University Medical Center Leipzig, 04103 Leipzig, Germany; 4 Department of Electrophysiology, Heart Center Leipzig at University of Leipzig, 04289 Leipzig, Germany; 5 Department of Cardiology, Median Centre for Rehabilitation Schmannewitz, 04774 Dahlen, Germany; 6 Institute of Biomedical Informatics, National Yang Ming Chiao Tung University, Taipei, 11221 Taiwan; 7 Pediatric Research Center, University Hospital for Children and Adolescents, Leipzig University, 04103 Leipzig, Germany; 8 Helmholtz Institute for Metabolic, Obesity and Vascular Research (HI-MAG) of the Helmholtz Zentrum München at the University of Leipzig and University Hospital Leipzig, 04103 Leipzig, Germany

## Abstract

**Background:**

RNA-seq emerges as a valuable method for clinical genetics. The transcriptome is “dynamic” and tissue-specific, but typically the probed tissues to analyze (TA) are different from the tissue of interest (TI) based on pathophysiology.

**Results:**

We developed Phenotype-Tissue Expression and Exploration (PTEE), a tool to facilitate the decision about the most suitable TA for RNA-seq. We integrated phenotype-annotated genes, used 54 tissues from GTEx to perform correlation analyses and identify expressed genes and transcripts between TAs and TIs. We identified skeletal muscle as the most appropriate TA to inquire for cardiac arrhythmia genes and skin as a good proxy to study neurodevelopmental disorders. We also explored RNA-seq limitations and show that on-off switching of gene expression during ontogenesis or circadian rhythm can cause blind spots for RNA-seq-based analyses.

**Conclusions:**

PTEE aids the identification of tissues suitable for RNA-seq for a given pathology to increase the success rate of diagnosis and gene discovery. PTEE is freely available at https://bioinf.eva.mpg.de/PTEE/

**Supplementary Information:**

The online version contains supplementary material available at 10.1186/s12864-021-08125-9.

## Background

Exome sequencing (ES) is a well-established method for diagnosing Mendelian disorders and improving precision medicine. Yet, ∼50–75% of patients remain undiagnosed after ES [1–6], although an underlying genetic disorder is highly suspected. Genome sequencing (GS) of patients offered a promising alternative, however, GS led to only a marginal increase in the yield compared to ES, with additional 10–15% of patients being diagnosed [4, 7–9]. The minimal boost of GS in the diagnostic yield is caused by a poor prioritization and interpretation of variants because of the lack of our current functional knowledge specifically of non-coding regions [3, 4, 8, 10, 11]. Hence, there is a growing interest of clinicians to use transcriptomics to facilitate variant interpretation [3, 11–13]. While DNA sequencing reveals variants in coding and non-coding regions, the investigator is still blind to the effect of regulatory variants that may affect RNA abundance and splicing. RNA-seq addresses this important gap by directly probing gene expression and splicing patterns. Yet, RNA-seq holds distinct limitations for the detection of DNA variants, made difficult by monoallelic expression or nonsense mediated decay. Thus, beginning experience with RNA-seq has proven successful in improving diagnosis of individuals with unresolved diagnosis after exome- or genome sequencing, when introduced complementary to GS or ES [3, 7, 11, 12, 14–16].

One major challenge of transcriptomics is tissue-specific gene expression [7, 14, 16]. In the endeavor of finding the diagnosis for the patient’s phenotype, clinicians are left with the decision of which tissue is most suitable to inquire, since biopsies to acquire the tissue of interest (TI) based on inferred pathophysiology are fairly rare. Recently, MAJIQ-CAT, a web-based tool has been designed to inform the tissue choice according to splicing pattern similarities between a clinically accessible tissue for analysis (TA) and a TI [7]. While the tool is very useful when the gene of interest is known, incorporation of human phenotype ontology [17] could prove additional utility for candidate gene identification and diagnosis [7].

We designed the online resource, Phenotype Tissue Expression and Exploration (PTEE), that incorporates data of 54 adult tissues from Genotype-Tissue Expression (GTEx) Project [18] and genes annotated to a multitude of phenotypes based on Phenomizer, human phenotype ontology (HPO) [17, 19], and expert opinion for neurodevelopmental (NDD)- [20], heart rhythm -[21, 22], and monogenic obesity-related disorders [23]. We identify tissues that are most suitable for performing RNA-seq in individuals with NDD or cardiac arrhythmia conditions. We show that genes annotated with these phenotypes are not necessarily expressed in the TI (e.g., NDD genes / inherited cardiac arrhythmia genes are not always expressed in the brain / heart). Our observations on gene expression correlations between distinct tissues could explain why, although blood is not considered to be a representative tissue for neurologic disorders, RNA-seq on blood proved successful for such conditions [12]. In summary, the present resource informs clinicians and scientists about which TA should be collected given the individual’s phenotype and a TI, provided a valid ethical and individual consent.

### Implementation

#### Data processing

Data processing was performed in R [24] version 4.0.3. We used gene median transcript per million counts (TPM) of 54 tissues and a total of 17,382 samples from GTEx version 8 (Supplementary Material – File 1 displays number of expressed genes as a function of number of samples/individuals per tissue). The gene expression analysis appears to be most robust and the number of expressed genes plateaus when the number of samples per tissue exceeds 100. For transcript specific expression, we considered the transcript TPMs and calculated the median per tissue. A gene was required to have TPM ≥ 1.5 to be considered expressed [25] and included in subsequent analyses.

Phenotype annotation data was obtained from Phenomyzer [17], SysID Database (release 1.1:2021-04-10) for primary NDD genes [20], the gene compilation by Gray and Behr for cardiac rhythm disorders [21], and the compilation by Rhode and colleagues for monogenic obesity disorders [23].

A workflow of the analysis is presented in Supplementary Material – File 2. Briefly, analyses can be restricted to gene lists annotated for specific phenotypes, custom input, or all genes expressed in a specific tissue. Further, the tissue of interest and the tissue of analysis are defined. To calculate Pearson’s correlation based on gene expression profiles only genes with TPM ≥ 1.5 are considered and the correlation is calculated as: ${r}_{xy}=\frac{\mathit{\operatorname{cov}}\left(x,y\right)}{SDx\times SDy}$, where *cov* is the covariance of the *x* = gene expression levels in the tissue of interest and *y* = gene expression levels in the tissue of analysis and SD = standard deviation of the variables [26]. To control for the influence of the tested genes on the correlation analysis we performed 100 randomization tests in which we calculated correlation coefficients based on random gene lists, which included the same number of genes as the real non-randomized gene list (e.g., 39 genes for inherited cardiac arrhythmias). We ran a binomial test to check whether the tissue that showed highest number of best correlations in the randomization tests was significantly different from the other tested TAs.

To identify which genes are expressed in the tissue of interest and the tissue of analysis, we considered only the genes included in the input list (phenotype of interest, custom genes, or all genes expressed in a specific tissue). For each considered tissue we then filtered the list of genes to include only the ones with TPM ≥ 1.5, considered to be expressed. To identify overlaps of gene expression in different tissues we used the merge function from R and identified common genes between the different tissues. If multiple tissues were input as TA/TI, genes were considered expressed if they were present in at least one of the considered tissues. For visualization of the graphs, we used ggplot [27] and venn diagram [28] packages.

In single gene analysis we display the expression of the gene in multiple samples and multiple tissues using violin graphs created with the package ggplot [27] – geom_violin from R.

The code and data for each analysis are deposited at the GitHub PTEE (https://github.com/akhilvelluva/PTEE) repository.

#### Web tool implementation and data access

We developed a user-friendly web interface using the R shiny package [29] – Phenotype Tissue Expression and Exploration (https://bioinf.eva.mpg.de/PTEE/). Graphics were generated using BioRender.com. Instructions for PTEE usage are presented in Supplementary Material – File 3. Users can select either a phenotype of interest based on an individual’s phenotype or input a list of genes to be inquired. The selection of the phenotype of interest restricts analyses to genes annotated with the respective HPO term, or genes that are annotated to be causative or candidates for NDD, heart rhythm-, or monogenic obesity-related disorders. Additionally, users can also upload custom gene lists according to their interests. Users can identify which genes belong to the HPO term in the table displayed online, with the possibility of download. Based on the phenotype the individual displays, users select a TI, which in general reflects the most affected organ and the disease pathophysiology.

Accessible TAs are: whole blood, skin, Epstein Barr virus (EBV)- transformed lymphocytes, cultured fibroblasts, and skeletal muscle. Users can visualize the correlation based on gene expression levels between TI and the TA, considering genes that are annotated to the individual’s phenotype. Based on random gene lists that contain the same number of genes as the one selected in the phenotype of interest, users can determine whether the tissue with best correlation coefficient generally performs best, or the correlation is influenced by the number of considered genes. Another feature of the tool allows users to visualize the overlap of expressed transcripts between TI and TA and to inquire the expression of each transcript in the two tissues. Also, the users can visualize which tissue expresses most genes included in the list they inquire.

In the gene expression analysis, users can directly visualize the expression of genes in different tissues.

#### Inquiry of heart rhythm disorders for tool validation

To validate the tool, we inquired genes related to inherited cardiac arrhythmias which are often induced by channelopathies. The underlying genetic defects can alter the ionic currents and change the shape and duration of the cardiac action potential [22]. Thus, most of the responsible genes are expressed in cardiomyocytes. Given the fact that the heart is a specialized muscle, among the easily accessible TAs skeletal muscle is expected to have the highest similarity to heart. Using analyses implemented within PTEE we prove this hypothesis, which also served as a sanity check for our tool.

#### Transcriptional profiling in the developing human brain and protein-protein interaction networks

To identify patterns of gene expression which are informative about neuronal developmental processes, we used the Allen Brain Atlas expression data and ABAEnrichment package implemented in R [30]. To this end, we identified the maximum expression in each developmental stage, followed by one-way ANOVA test to establish significance and Tukey’s HSD for the pairwise comparisons between the different groups, using the R-implemented corresponding functions [24]. The code for this analysis has been deposited under http://rpubs.com/Akhil_Velluva/ptee_aba.

We performed protein-protein interaction network (PIN) functional enrichment analysis of genes not expressed in the TI to delineate molecular processes in which these genes are involved. We incorporated the protein interaction partners of these genes to increase the power of functional module identification [31]. Functional annotations of genes were obtained from Gene Ontology (GO) [32] and protein-protein interaction data from InBio Map [33]. We then used a hypergeometric test to determine the enrichment of genes (conventional) and functional PPIs (network-wise) involved in the functional modules. The functional PPIs are interactions formed by two genes with the same GO annotation. We adjusted network-wise *p*-values using the Benjamini and Hochberg multiple testing procedures [34]. Functional modules with 1) adjusted conventional *p*-value < 0.05, 2) adjusted network-wise *p*-value < 0.05, and 3) at least one studied gene were considered to be significantly enriched.

## Results

### Skeletal muscle is the most appropriate accessible tissue to inquire for cardiac arrhythmias

The greatest challenge faced by clinicians and researchers in the transition to RNA-seq-based diagnostics is the tissue-specific gene expression [14]. Thus, both the underlying pathology and the expected most representative tissue must be factored in the decision regarding which tissue should be analyzed by RNA-seq. The choice can prove very complicated in the case of heterogeneous phenotypes for which a representative tissue is hard to establish, e.g., in the case of hereditary cancer syndromes.

Hence, for the validation of the resource we developed, we initially chose a homogenous phenotype – inherited heart rhythm disorders – which involves a highly specialized organ – the heart. The phenotype is very suitable to validate the approach of our tool for two reasons: (i) inherited cardiac arrhythmias are often a result of perturbed ionic channels, generally expressed in the heart – thus, the choice of TI is very clear; (ii) the heart is a highly specialized muscle, hence the accessible TA expected to be most similar is skeletal muscle.

**Fig. 1:**
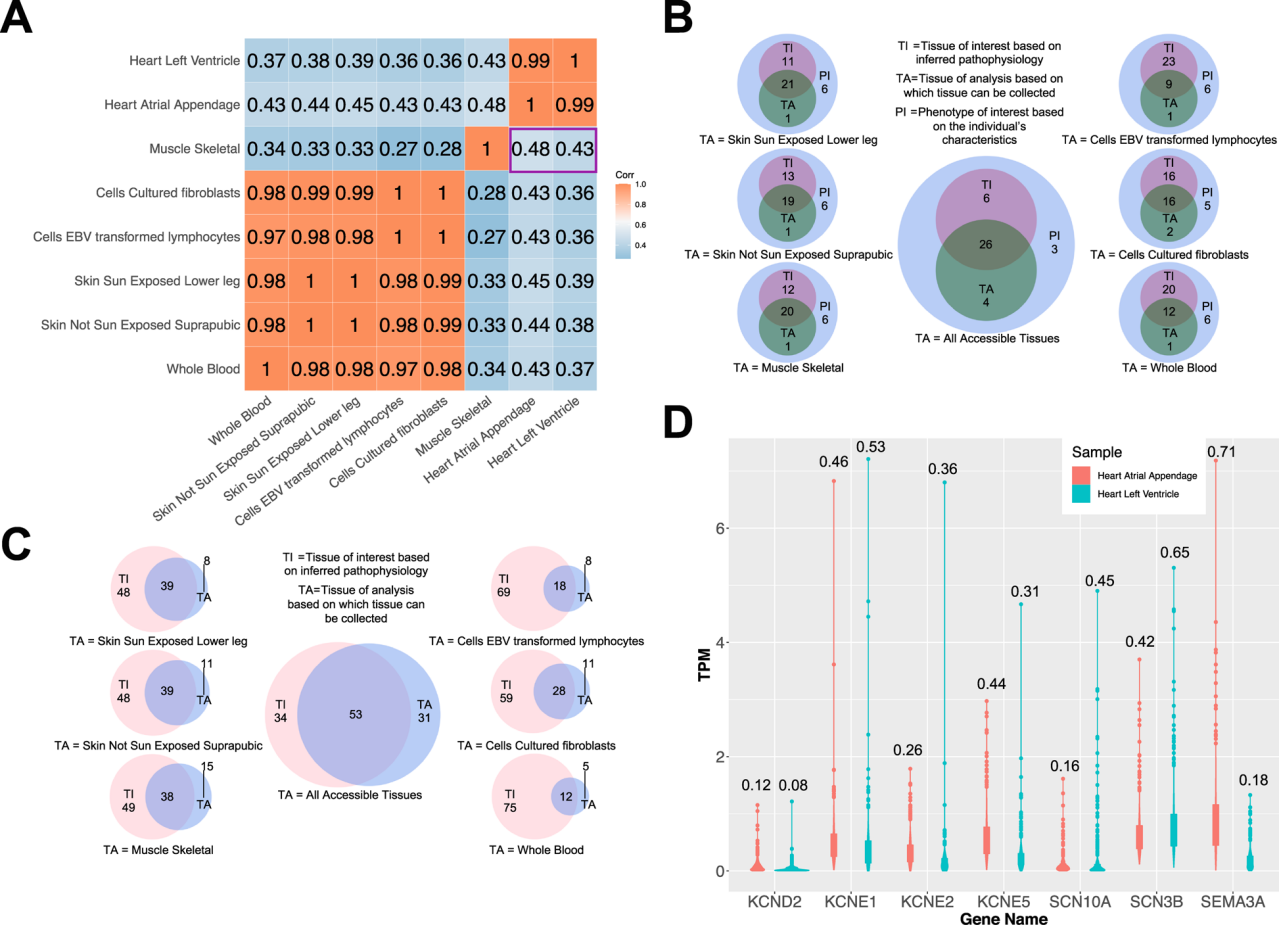
Most appropriate tissue of analysis to inquire for cardiac arrhythmias. **A.** Correlation analysis based on genes with median TPM > 1.5 between the TI (heart) and all easily accessible TAs. The highest correlation coefficient between TI and TAs was obtained by skeletal muscle (correlation coefficients marked in the purple rectangular: *r* = 0.43 for the heart left ventricle and *r* = 0.48 heart atrial appendage). **B.** Venn diagrams showing the number of expressed genes (median TPM > 1.5) in TI (heart) and different TAs. Skin and skeletal muscle express most of the genes included in the “Cardiac Arrhythmia” list. When all TAs are considered, 30 of the 39 genes annotated in the “Cardiac arrhythmia” list (PI = phenotype of interest) are captured and only 9 genes are not covered in any of the TAs. **C.** Venn diagrams showing the number of transcripts (median TPM > 1.5) in TI (heart) and different TAs. Using the “Cardiac Arrhythmia” list, skin and skeletal muscle show the highest number of overlapping transcripts with heart. **D.** Violin plot of genes with low cardiac expression (median TPM < 1.5). Numbers above violins represent standard deviation. The only gene with low expression and low variability is *KCND2*

To test this we used the “Cardiac Arrhythmia” list, which includes 39 genes, reviewed by Gray and Behr [21]. We show that, based on expression levels, the highest correlation occurs, as expected, for skeletal muscle in comparison to all other accessible TAs (*r* = 0.48 compared to other accessible TAs with r ≤ 0.45 Fig. 1A). Also, in multiple randomization tests *p*-value was lower than 0.001, suggesting that skeletal muscle compared to the other accessible TAs generally correlates better with heart. Beside the correlation analysis based on gene expression levels, it is important to know how many of the target genes are expressed by the TI and TA to identify blind spots of the analysis. This analysis showed that skeletal muscle and skin are the accessible TAs with highest overlap of genes expressed in the TI – heart (20 and 21 genes, respectively, compared to all other accessible TAs with ≤16 genes, Fig. 1B). Moreover, skin and skeletal muscle are also the accessible TAs which share most transcripts with heart (39 and 38 transcripts, respectively, compared to all other accessible TAs with ≤28 transcripts, Fig. 1C). Furthermore, we show that if multiple TAs are sequenced the yield of expressed genes overlapping with TI is higher.

Based on the “Expression Analysis” tab, we identified 7 genes with no cardiac expression (median TPM < 1.5, Table 1). Thus, we used the “Single gene analysis” tab to visualize expression levels of those genes. Except for *KCND2*, all other genes displayed a very high variability in expression levels in the heart (Fig. 1D); individuals with high expression levels of those genes could have increased susceptibility to cardiac arrhythmia. Next, we identified which PINs are significantly enriched among the genes with low heart expression. We observed an enrichment of functional modules related to regulation of heart contraction and ion transport for potassium channel encoding genes *KCND2*, *KCNE1*, *KCNE2*, and *KCNE5* (Supplementary Material – Supplementary Table). Furthermore, we identified significantly enriched functional modules formed by PINs of *SEMA3A* partners. These were related to regulation of neurogenesis and sympathetic neuron projection (Supplementary Material – Supplementary Table), which suggests an indirect effect of this gene on heart function.

**Table 1:** Genes annotated for cardiac arrhythmias or NDD with very low expression (median TPM < 1.5) in the TI. Genes marked in bold show highest expression in brain during the prenatal stage of development. Underlined genes are not expressed in brain in any of the developmental stages

**Cardiac arrhythmia genes not expressed in heart**	*KCNE2*, *KCNE5*, *SCN10A*, *SEMA3A*, *SCN3B*, *KCNE1*, *KCND2*
**NDD genes not expressed in adult brain**	***STRA6***, *ARSE*, *TM4SF20*, *SLC6A19*, *CA5A*, ***GSX2***, ***ZBTB20***, ***NEUROG1***, *SCN10A*, *KPNA7*, *AGMO*, *HIST1H4C*, ***TAT***, *RNU4ATAC*, *RMRP*, ***IGF1***, *ASPM*, ***GLI2***, ***WDR62***, ***ORC1***, ***KIF4A***, *UPB1*, *HOXA1*, ***CENPF***, *KIF14*, *TWIST1*, ***STIL***, ***FOXP2***, ***KIF11***, ***CEP55***, ***CENPE***, ***GATA6***, ***SIK1***, ***PLK4***, *FANCD2*, *MAT1A*, ***BUB1B***, *HPD*, *HIST1H1E*, ***CKAP2L***, ***ESCO2***, ***CCBE1***, ***FAT4***, *OCLN*, *MIR17HG*, ***ALG11***

Thus, using our tool we confirmed our initial hypothesis that skeletal muscle is the most suitable TA as proxy for heart. Yet, based on our results skin appears to be another TA suitable to test genes related to inherited cardiac arrhythmia. At a deeper exploration of genes involved in cardiac arrhythmias we identified those with lower and variable cardiac expression and with potential indirect effects.

### Skin is the most suitable accessible tissue for RNA-seq testing in individuals with neurodevelopmental disorders (NDD)

**Fig. 2:**
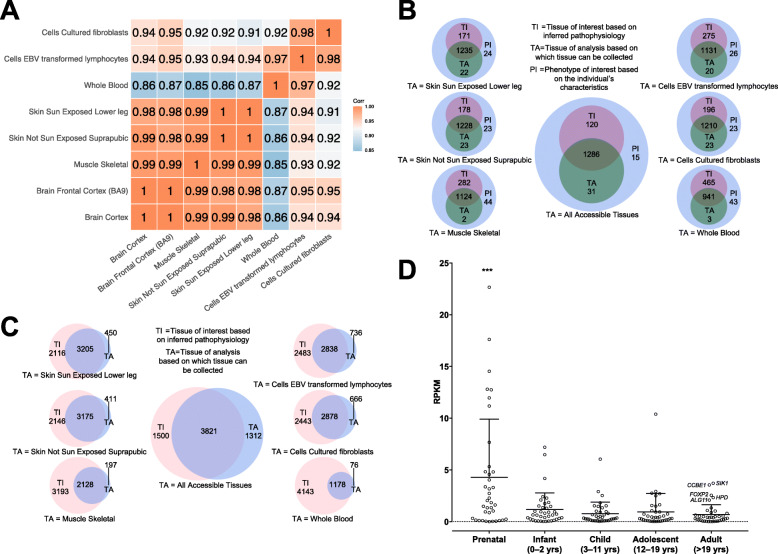
Most appropriate tissue of analysis to inquire for neurodevelopmental disorders. **A**. Correlation analysis based on genes with median TPM > 1.5 between the TI (different regions of brain cortex) and all easily accessible TAs. The highest correlation coefficient was obtained by skin (*r* = 0.99). **B**. Based on the “NDD Genes from SysID (Primary ID Genes)” set, the Venn diagrams show the number of expressed genes (median TPM > 1.5) in TI (central nervous system) and different easily accessible TAs. Skin shows the highest number of expressed genes (86.6%). When all easily accessible TAs are considered, 1317 of the 1452 genes annotated in the NDD list (PI = phenotype of interest) are captured and only 135 genes are not covered in any of the considered TAs. **C**. Venn diagrams showing the number of transcripts (median TPM > 1.5) in TI (central nervous system) and different TAs. Based on the NDD gene list, skin shows the highest number of overlapping transcripts with TI. **D**. Expression in different developmental stages of genes not expressed in adult brain (GTEx median TPM < 1.5). There is a significant enrichment of genes with a maximum expression level in the prenatal stage (Table 1). Based on Allen Brain Atlas data *SIK1*, *CCBE1*, *FOXP2*, *HPD*, and *ALG11* are expressed in the adult brain. *** *p*-value < 0.0001 RPKM = Reads Per Kilobase of transcript, per Million mapped reads

To evaluate which is the most appropriate accessible TA for RNA-seq in individuals with NDD, we used the list of genes from SysID [20]. This is an expert curated database, which includes genes with an already established genotype-phenotype correlation. We initially considered all central nervous system (CNS) tissues as TI and performed correlation analyses based on expression levels of all easily accessible TAs. The highest correlation coefficient was attained by skeletal muscle and skin. However, in multiple randomization none of the accessible TAs reached significance, suggesting that the correlation with the central nervous system can suffer considerable variation depending on the chosen gene list. While blood seemed least suitable to inquire gene expression of NDD-genes, the correlation coefficient to areas of the CNS was still very high (≥ 0.86, Fig. 2A). Next, we show that skin is the TA which shares most expressed NDD genes (85%) with CNS (Fig. 2B). Furthermore, most transcripts expressed in CNS could be recovered in skin, while the overlap between CNS and whole blood was lowest (Fig. 2C). Interestingly, 46 (3%) of the NDD genes are not expressed in brain (median TPM < 1.5), of which 3 (*FANCD2*, *HPD*, *HIST1H1E*) show blood expression (Fig. 2B).

### Genes involved in NDD and not expressed in the adult brain show significantly higher expression during prenatal brain development

To determine whether NDD genes, which are not expressed in the adult brain based on GTEx data are active during brain organogenesis or different developmental stages we inquired the Allen Human Brain Atlas [35] using the ABAEnrichment package [30]. We observed a significantly higher expression of these genes in the prenatal stage of brain development (*p*-value < 0.0001, Fig. 2D) followed by an apparent switch-off of the majority of these genes in the stages immediately after birth. While based on GTEx data *ALG11*, *CCBE1*, *FOXP2*, *HPD*, and *SIK1* are not expressed in the adult brain, based on Allen Human Brain Atlas data from 6 donors they show brain expression (Fig. 2D). This agrees with our observation based on the GTEx data that the number of expressed genes shows more variation when there are less than 100 samples considered (Supplementary Material – File 1).

Yet, 8 genes showed no brain expression during any of the inquired developmental stages (Table 1). To understand how these genes are involved in nervous system development we performed a PIN analysis. We identified a significant enrichment of functional modules formed by PIN partners of *KIF14*, *FANCD2*, and *OCLN* (Supplementary Material – Supplementary Table). These modules were related to apoptosis, including neuronal apoptotic processes, or DNA repair and cell-cell junction assemblies.

## Discussion

RNA-seq is on its way to be integrated into clinical laboratory genomics, since it holds the promise to facilitate the interpretation of variants of unclear significance [36]. Previous studies have touched on the potential of RNA-seq to enable rare disease diagnosis as well as novel gene discovery [11, 12, 14], but the extent to which RNA-seq is useful and when alternative approaches are needed remains largely unknown. The tissue-specific splicing pattern has been regarded as the major concern when using a TA as proxy for a TI based on the individual’s phenotype and inferred disease pathophysiology [7]. While clinicians already have access to tools that allow splicing pattern comparisons between different tissues [7], the decision of which TA is most suitable for RNA-seq given a specific pathology is still largely uninformed.

Here, we provide custom gene lists based on Human Phenotype Ontology [17, 19] and expert opinion [20, 21, 23], which enable clinicians to restrict analyses to genes related to a specific phenotype. We show that while clinicians may consider a specific tissue as relevant for the observed pathology (e.g., brain for NDD or heart for cardiac arrhythmias), it is not mandatory that the disease-causing gene is expressed in that tissue. Interestingly, for both phenotypes that we inquired closely – cardiac arrhythmias and NDD – we identified genes which are not expressed in the TI (Table 1).

Our results suggest that skeletal muscle or skin are the TA which best represent heart gene expression (Fig. 1). We identified genes encoding for ionic channels, with very low expression levels (median TPM < 1.5), which display a high variability in the heart (Fig. 1D). One possible explanation for their involvement in cardiac arrhythmias, despite their general low cardiac expression, is that given their increased variability, individuals at risk for heart rhythm pathologies could show higher expression levels. Interestingly, *KCND2*, which encodes the pore-forming subunit of the Kv4.2 cardiac potassium channel involved in the repolarization phase of the ventricular action potential, displays low variability and low expression levels in the heart (Fig. 1D). Gain-of-function variants in *KCND2* have been implicated in nocturnal atrial fibrillation [37]. The nocturnal occurrence of symptoms was attributed to the circadian variation of Kv4.2 in murine hearts with a substantial 2-fold change in expression between night and day [38]. Hence, condition-dependent variation of gene expression adds another layer of complexity for RNA-seq approaches, in addition to tissue-specific expression. Based on our results inquiry of TI does not guarantee that all genes are properly represented; e.g., we identified *SEMA3A*, which is poorly expressed in heart (Fig. 1D) to be involved in sympathetic neuron projection (Supplementary Material – Supplementary Table). This suggested an indirect role of this gene in the generation of cardiac arrhythmias. Indeed, *SEMA3A* has been indirectly implicated in cardiac arrest and ventricular fibrillation [39] by affecting the cardiac sympathetic innervation [40]. Based on our results the most suitable tissues for RNA-seq analyses with the aim to inquire inherited cardiac arrhythmias are skeletal muscle and skin. Yet, inherited cardiac arrhythmias can be accompanied by abnormalities in the skeletal muscle [41], which may affect the expression profile. Still, the comparison to the normal state can aid the identification of expression outliers [42].

Furthermore, we focused on NDD genes and the identification of the most appropriate TA for RNA-seq, considering brain as TI. Our results suggested that the best proxy for brain given the considered easily accessible TAs is skin (Fig. 2). This is also supported by embryonic gene expression profiles which show higher clustering of the surface ectoderm (precursory of skin) and neuroectoderm (precursory of CNS) compared to blood mesoderm [43]. As in the case of cardiac arrhythmias we identified genes which do not show expression in any of the adult brain areas (Table 1). Among these, based on the Allen Brain Atlas [30] data, there is a significant enrichment (*p*-value < 0.0001) of genes with highest expression during the prenatal stage followed by silencing in the other developmental stages (Fig. 2D). Similar to the previous example of genes with expression levels influenced by circadian rhythm, this result brings awareness to the difficulties of RNA-seq-based studies. Thus, a gene which may be relevant in organogenesis and hence for a specific pathology, in this case NDD, can be turned off during adulthood. Such genes will be blind spots for RNA-seq-based diagnosis when only the TI is inquired.

Interestingly, for some of these genes whole blood RNA-seq would be a better option to increase the chances of detecting expression of specific transcripts, or alterations in gene expression (Fig. 2B). For example, 3 NDD genes (*FANCD2*, *HPD*, *HIST1H1E* – identified using the table in the “Expression Analysis” tab) are expressed in blood, but not in the central nervous system (Fig. 2C). Pathogenic variants of all three genes cause syndromic diseases, where the CNS symptoms represent only a part of the clinical picture. An example of an indirect effect on CNS is *HIST1H1E*, which encodes histone H1.4 that regulates the accessibility of regulatory proteins to the target sites and DNA; pathogenic variants in this gene cause epigenetic modifications of genes that are highly expressed in brain tissues [44], influencing indirectly the CNS.

Still, the overall high correlation in expression levels of NDD genes between blood and brain (r = 0.86, Fig. 2A) may explain why Frèsard and colleagues had a higher-than-expected rate of success for gene identification in neurological cases on blood RNA, although this is not assumed to be a representative tissue for the pathology [12].

## Conclusions

We provide a tool which, based on the individual’s phenotype and an inferred tissue of interest, facilitates an informed decision regarding the most suitable TA for RNA-seq, according to gene expression correlations and number of expressed genes/transcripts. Our results suggest that there is no perfect tissue to analyze for a specific pathology. Counterintuitively, the TI does not hold a 100% guarantee that the disease-causing gene is well represented. This could be related to the fact that gene expression is dynamic during ontogenesis since transcription and expression levels can change during cell differentiation and the transition between different developmental stages [45, 46]. We show that RNA-seq-based studies can be complicated by condition-specific expression patterns, switching on-and-off of a gene, or even indirect effects on expression profiles. The webtool we developed helps clinicians and scientists to directly explore these limitations and identify the most suitable tissue or combination of tissues to increase the success rate of RNA-seq based analyses.

## Supplementary Information

**Additional file 1: Supplementary Material – File 1**: Displays number of expressed genes as a function of number of samples/individuals per tissue.

**Additional file 2: Supplementary Material – File 2**: Scheme of PTEE workflow. (Original image created with BioRender.com, Agreement for Publication License CA233O9R60)

**Additional file 3: Supplementary Material – File 3**: Instructions for using PTEE.

**Additional file 4: Supplementary Table**: PIN analysis results of selected genes.

## Data Availability

All datasets used in this study were obtained from the Genotype-Tissue Expression (GTEx) project (https://gtexportal.org/home/datasets), SysID database (https://www.sysid.dbmr.unibe.ch/table/overview), and HPO (https://raw.githubusercontent.com/obophenotype/human-phenotype-ontology/master/hp.obo).

## Abbreviations

PTEE: Phenotype Tissue Expression and Exploration
HPO: Human phenotype ontology
NDD: Neuro development disorder
PI: Phenotype of interest
PIN: Protein-protein interaction network
TA: Tissue of analysis
TI: Tissue of interest
