# Supplementary material for: Phenotype-tissue expression and exploration (PTEE) resource facilitates the choice of tissue for RNA-seq-based clinical genetics studies
Source: BMC Genomics. 2021 Nov 7;22:802. doi: 10.1186/s12864-021-08125-9 (PMC8573933; doi:10.1186/s12864-021-08125-9)
Supplement: Supplementary file 1 — Additional file 1: Supplementary Material – File 1: Displays number of expressed genes as a function of number of samples/individuals per tissue. [file 12864_2021_8125_MOESM1_ESM.pdf]

Phenotype-Tissue Expression and Exploration (PTEE) resource facilitates the choice of tissue for RNA-seq-based clinical genetics studies

Akhil Velluva [1,2*], Maximillian Radtke [3], Susanne Horn [2], Bernt Popp [3], Konrad Platzer [3], Erind Gjermeni [4,5], Chen-Ching Lin [6], Johannes R. Lemke [3], Antje Garten [7], Torsten Schöneberg [2], Matthias Blüher [8], Rami Abou Jamra [3], Diana Le Duc [1,3,8*]

Supplementary File 1

To determine whether the number of expressed genes can be influenced by the number of available samples/individuals per tissue we plotted the number of expressed genes (median TPM > 1.5) when sequentially incrementing the number analyzed samples by 1 (see graphs below).

We observed that for low number of samples the variation in the number of expressed genes is higher. The number of expressed genes reaches saturation for sample numbers over 100. This suggests that for tissues with low number of available samples: kidney cortex, kidney medulla, bladder, endocervix, ectocervix, and fallopian tube the analyses (correlation of gene expression between tissues, number of expressed genes, number of expressed transcripts) may display high variation.

**Tissue Name**	**Number of RNAseq Samples (GTEx)**	**Tissue Name**	**Number of RNAseq Samples (GTEx)**	**Tissue Name**	**Number of RNAseq Samples (GTEx)**
Muscle – Skeletal	803	Stomach	359	Ovary	180
Whole Blood	755	Testis	361	Brain – Hippocampus	197
Skin – Sun Exposed (Lower	701	Colon – Sigmoid	373	Brain – Anterior cingulate cortex	176
Artery – Tibial	663	Pancreas	328	Cells – EBV–transformed lymphocytes	174
Adipose – Subcutaneous	663	Pituitary	283	Minor Salivary Gland	162
Thyroid	653	Adrenal Gland	258	Vagina	156
Nerve – Tibial	619	Spleen	241	Brain – Amygdala	152
Skin – Not Sun Exposed	604	Prostate	245	Uterus	142
Lung	578	Artery – Coronary	240	Brain – Spinal cord (cervical c–1)	159
Esophagus – Mucosa	555	Brain – Cerebellum	241	Brain – Substantia nigra	139
Cells – Cultured fibroblasts	504	Liver	226	Kidney – Cortex	85
Adipose – Visceral	541	Brain – Cortex	255	Bladder	21
Esophagus – Muscularis	515	Brain – Nucleus accumbens (basal ganglia)	246	Cervix – Endocervix	10
Breast – Mammary Tissue	459	Brain – Caudate (basal ganglia)	246	Cervix – Ectocervix	9
Artery – Aorta	432	Brain – Cerebellar Hemisphere	215	Fallopian Tube	9
Heart – Left Ventricle	432	Brain – Frontal Cortex (BA9)	209	Kidney – Medulla	4
Heart – Atrial Appendage	429	Small Intestine – Terminal Ileum	187		
Colon – Transverse	406	Brain – Hypothalamus	202		
Esophagus –	375	Brain – Putamen (basal ganglia)	205		

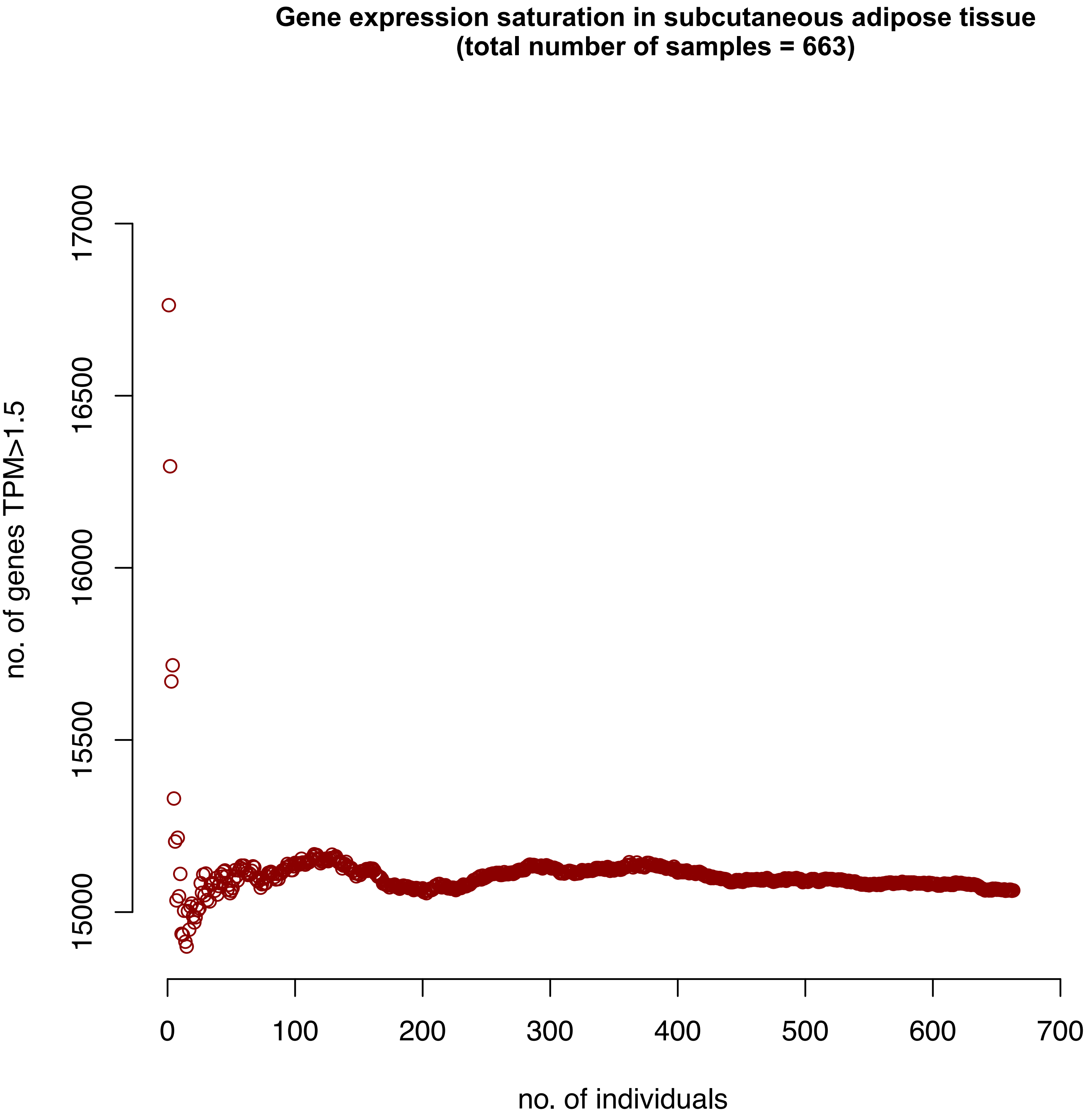
Gene expression saturation in subcutaneous adipose tissue
(total number of samples = 663)
no. of genes TPM>1.5
17000
16500
16000
15500
15000
0
100
200
300
400
500
600
700
no. of individuals

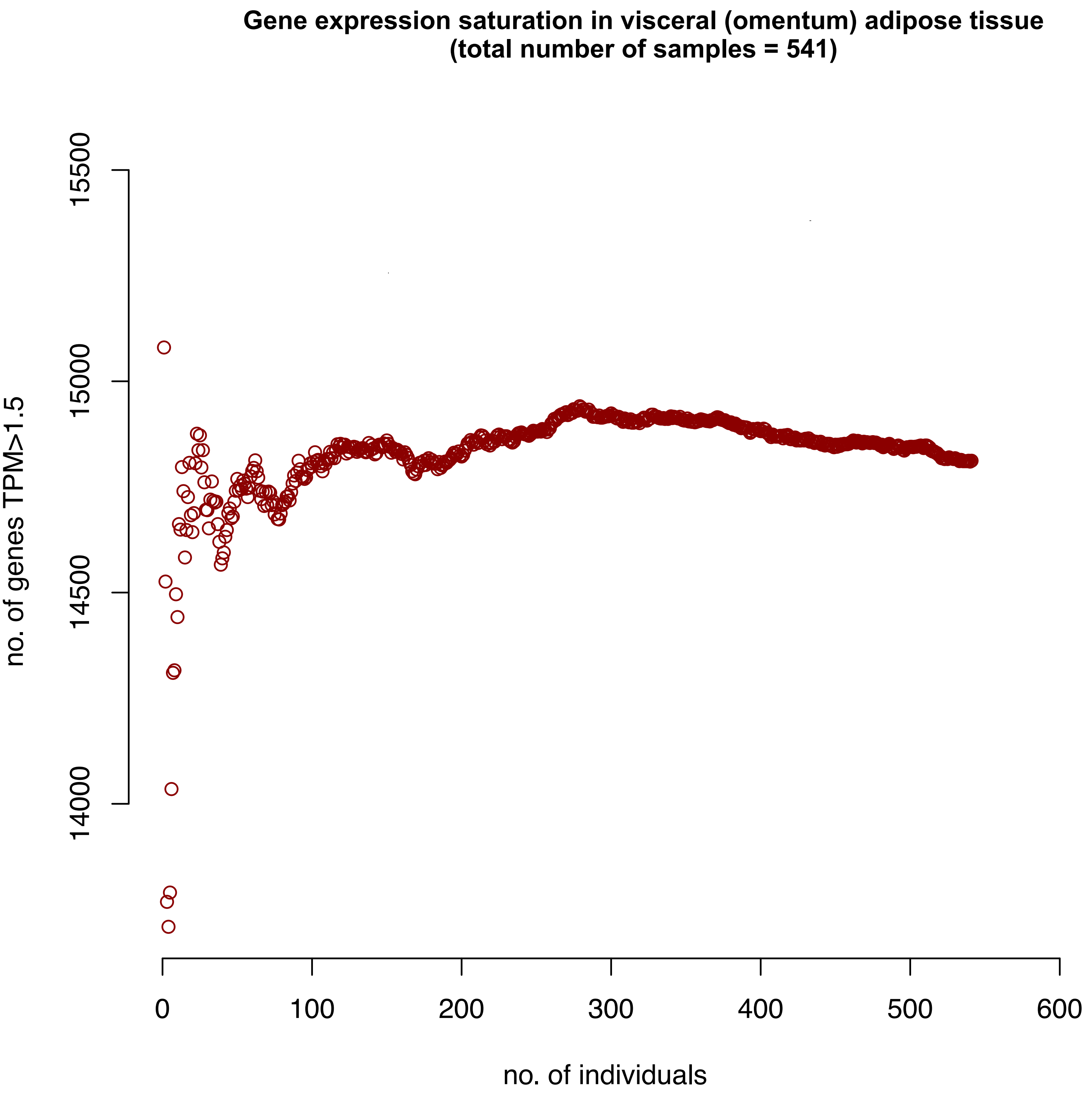
Gene expression saturation in visceral (omentum) adipose tissue
(total number of samples = 541)
no. of genes TPM>1.5
14000
14500
15000
15500
0
100
200
300
400
500
600
no. of individuals

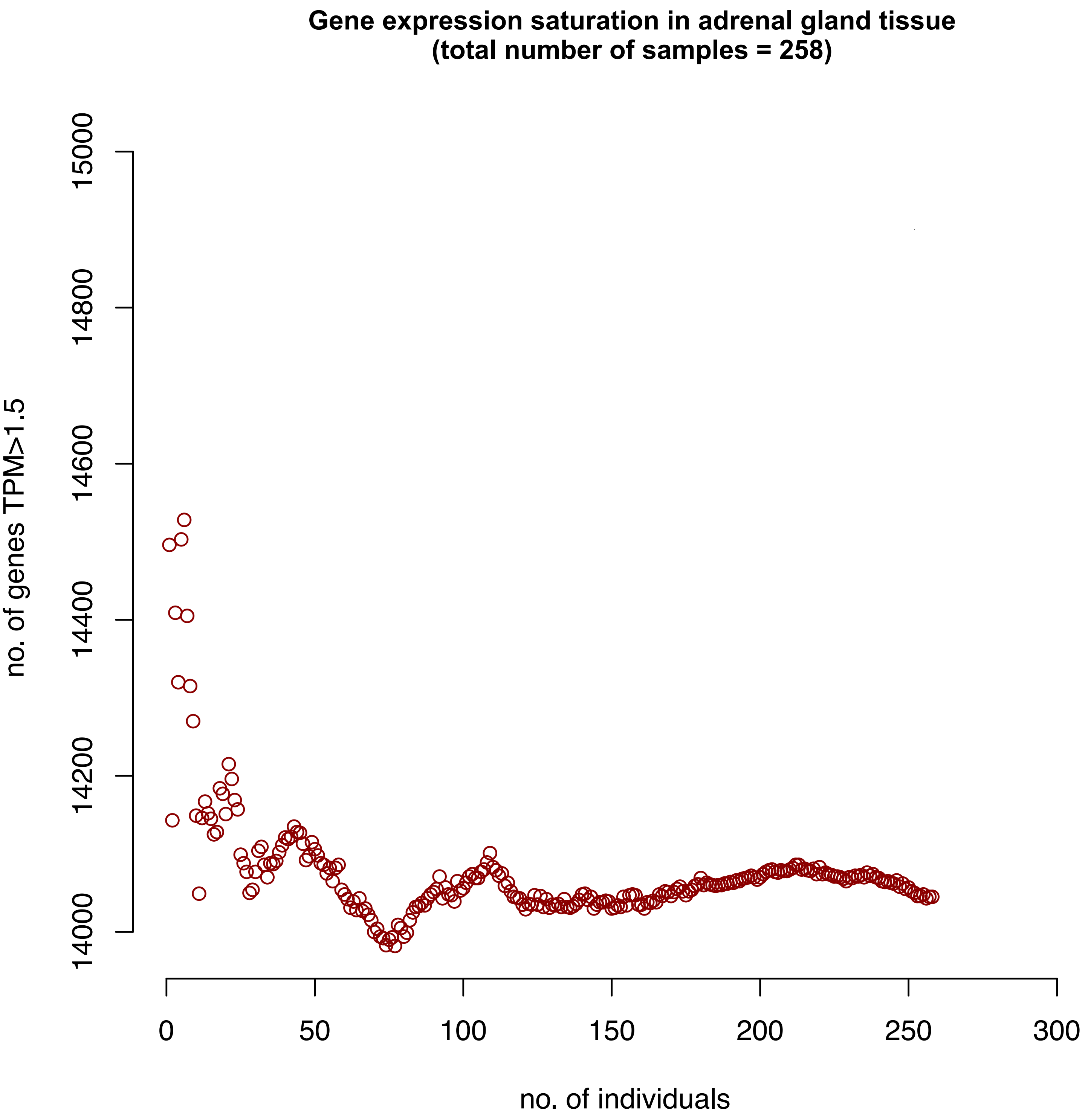
Gene expression saturation in adrenal gland tissue
(total number of samples = 258)
no. of genes TPM>1.5
14000
14200
14400
14600
14800
15000
0
50
100
150
200
250
300
no. of individuals

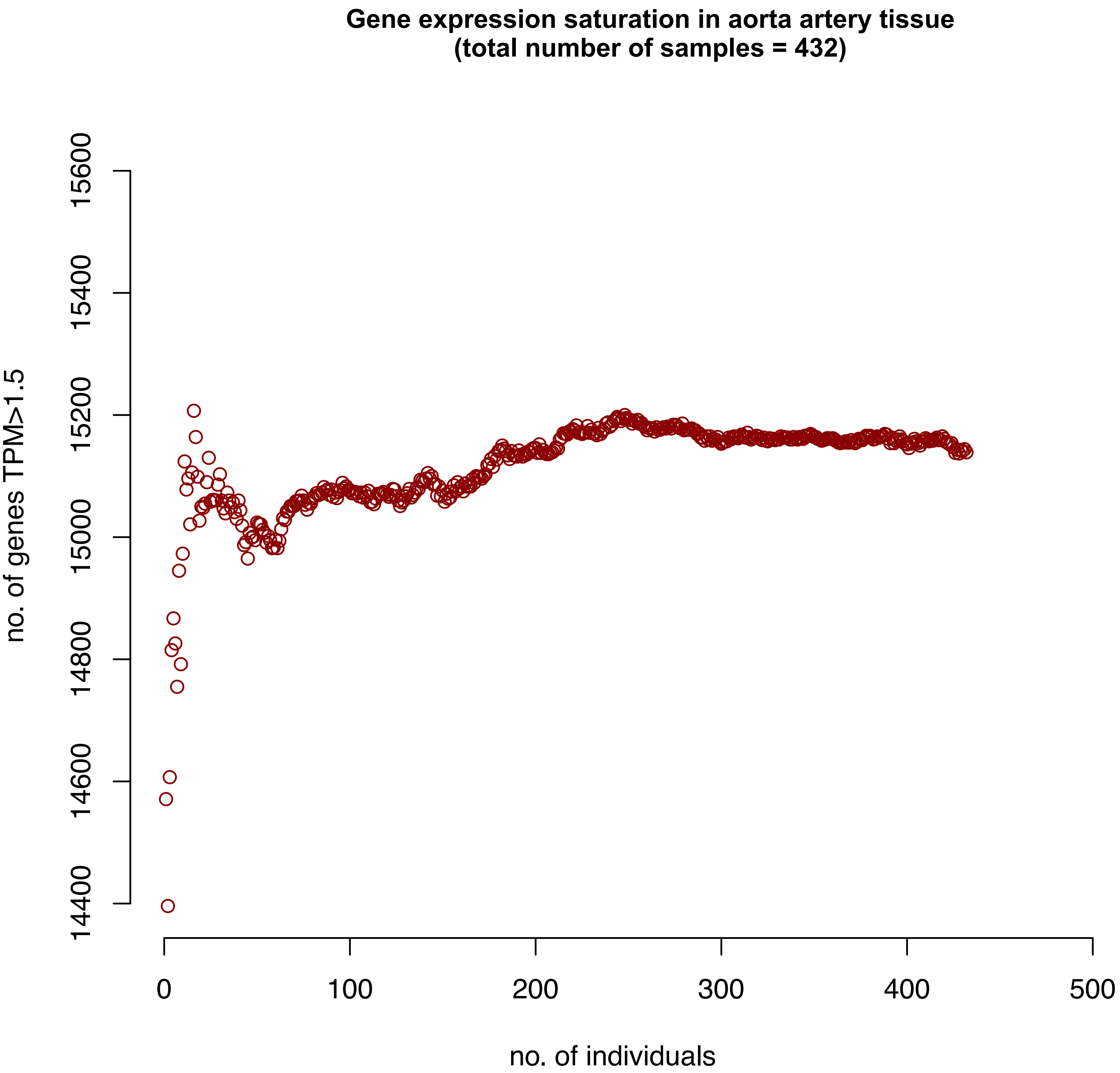
Gene expression saturation in aorta artery tissue
(total number of samples = 432)
no. of genes TPM>1.5
14400
14600
14800
15000
15200
15400
15600
0
100
200
300
400
500
no. of individuals

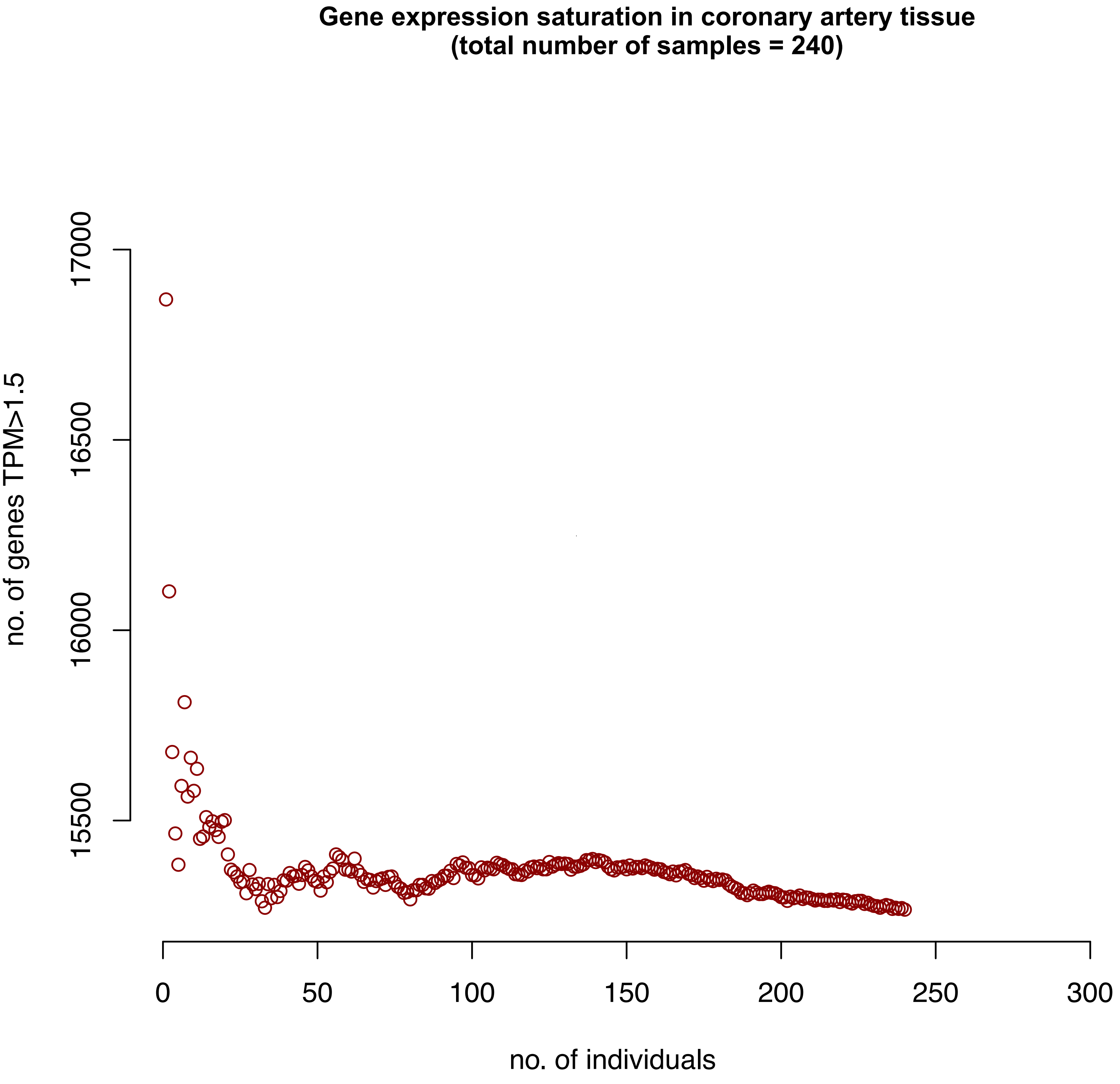

Gene expression saturation in coronary artery tissue
(total number of samples = 240)
no. of genes TPM>1.5
17000
16500
16000
15500
0
50
100
150
200
250
300
no. of individuals

Gene expression saturation in tibial artery tissue (total number of samples = 663)

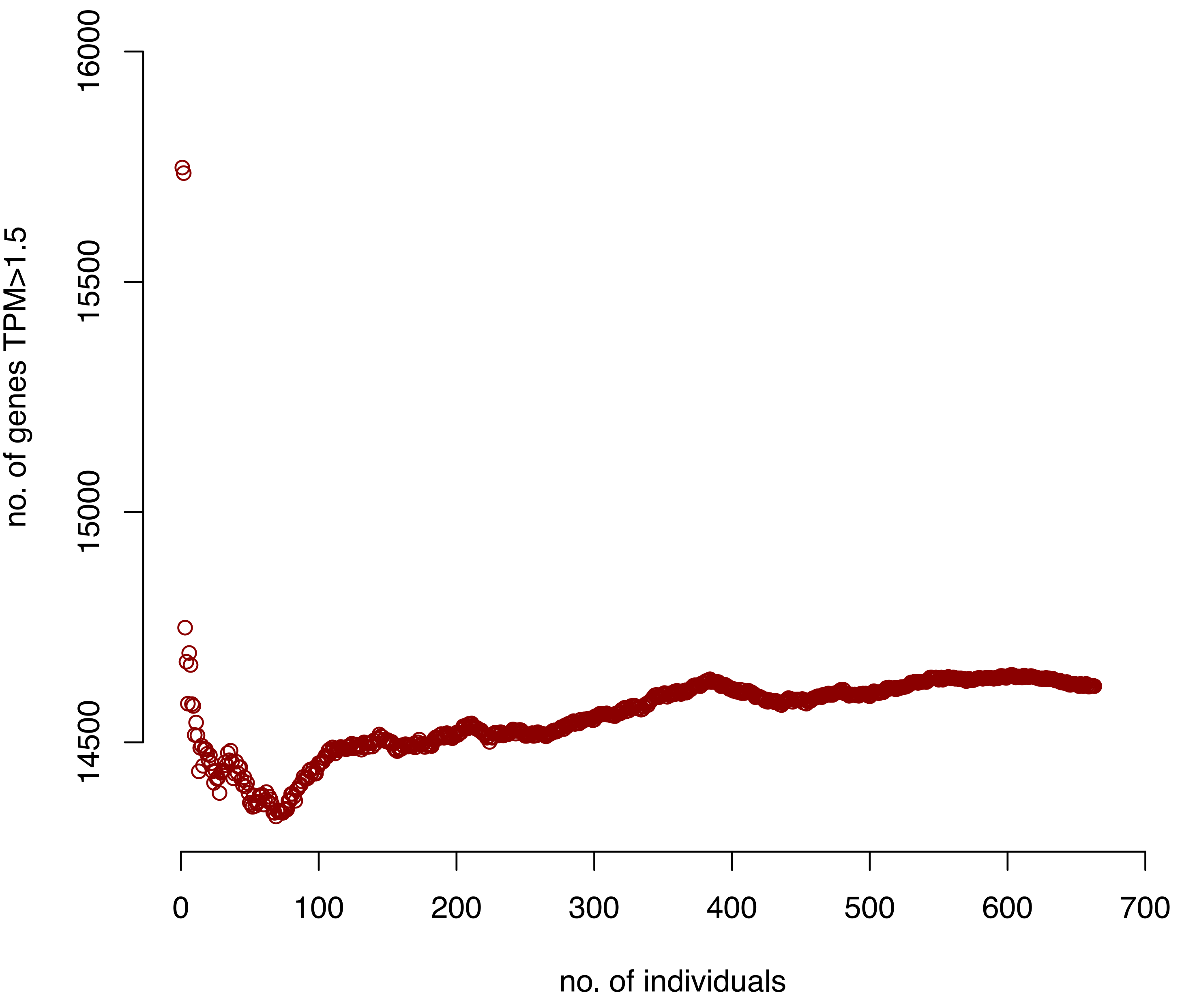

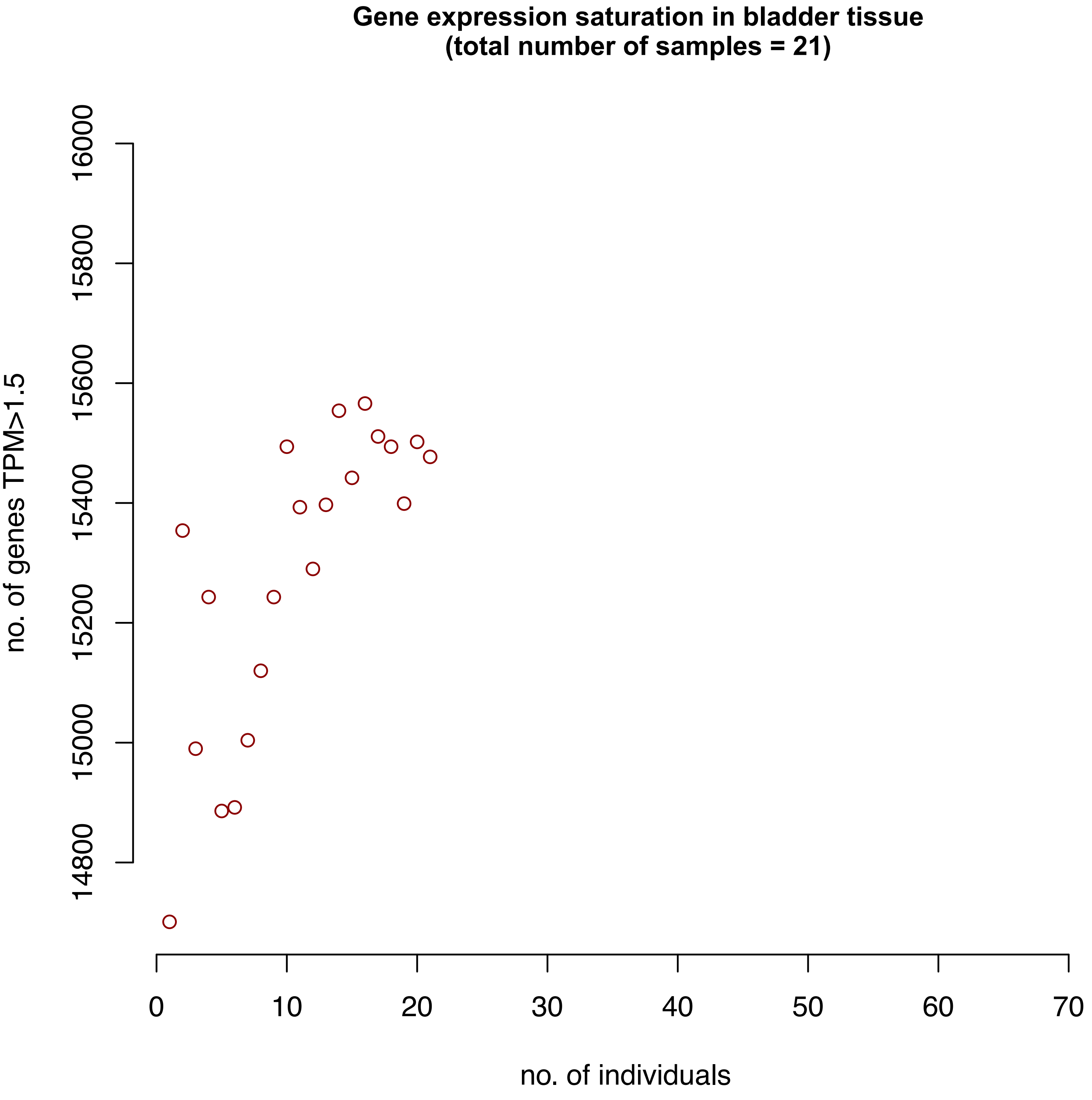

Gene expression saturation in bladder tissue
(total number of samples = 21)
no. of genes TPM>1.5
14800
15000
15200
15400
15600
15800
16000
0
10
20
30
40
50
60
70
no. of individuals

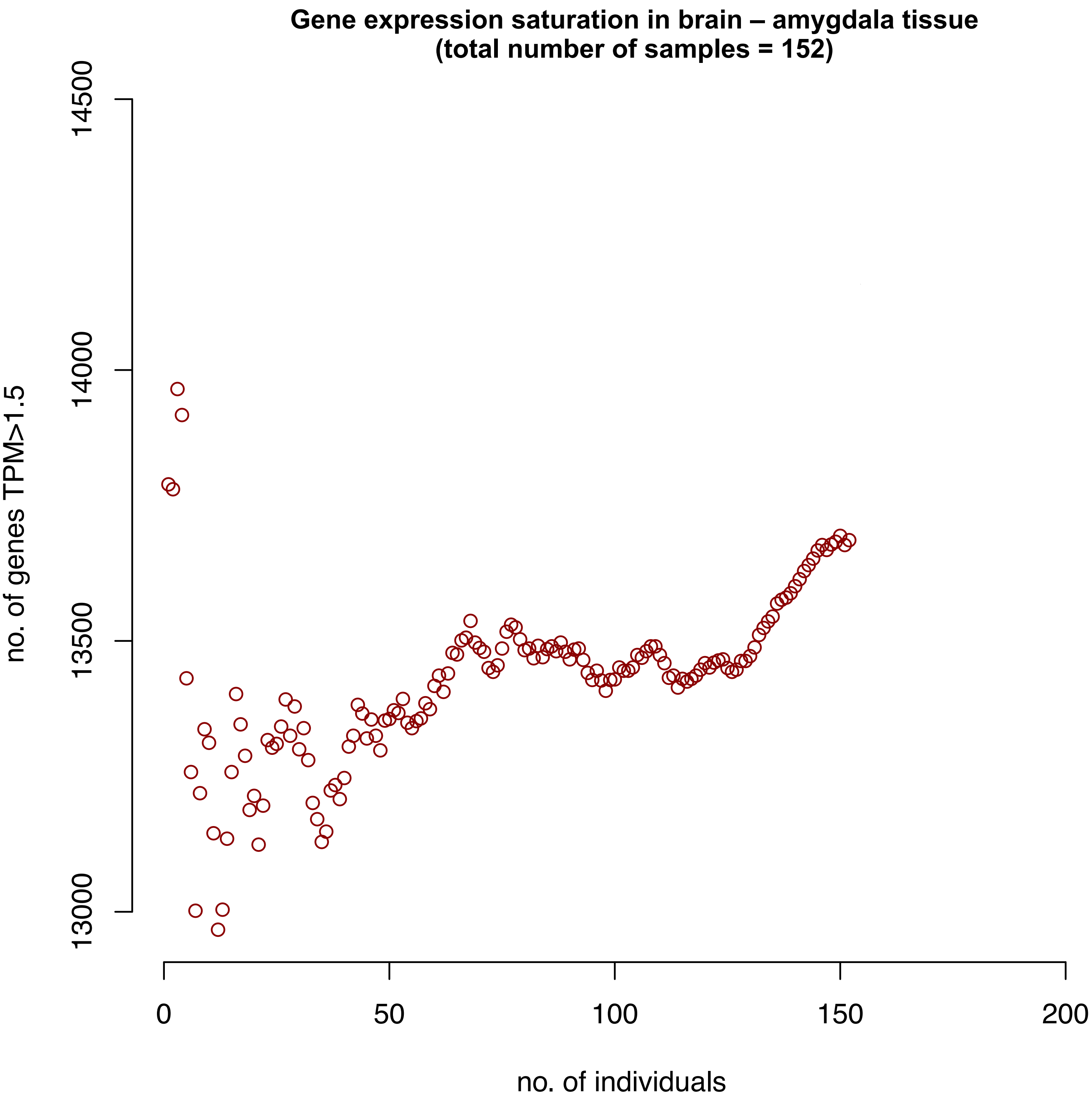

Gene expression saturation in brain – amygdala tissue
(total number of samples = 152)
no. of genes TPM>1.5
14500
14000
13500
13000
0
50
100
150
200
no. of individuals

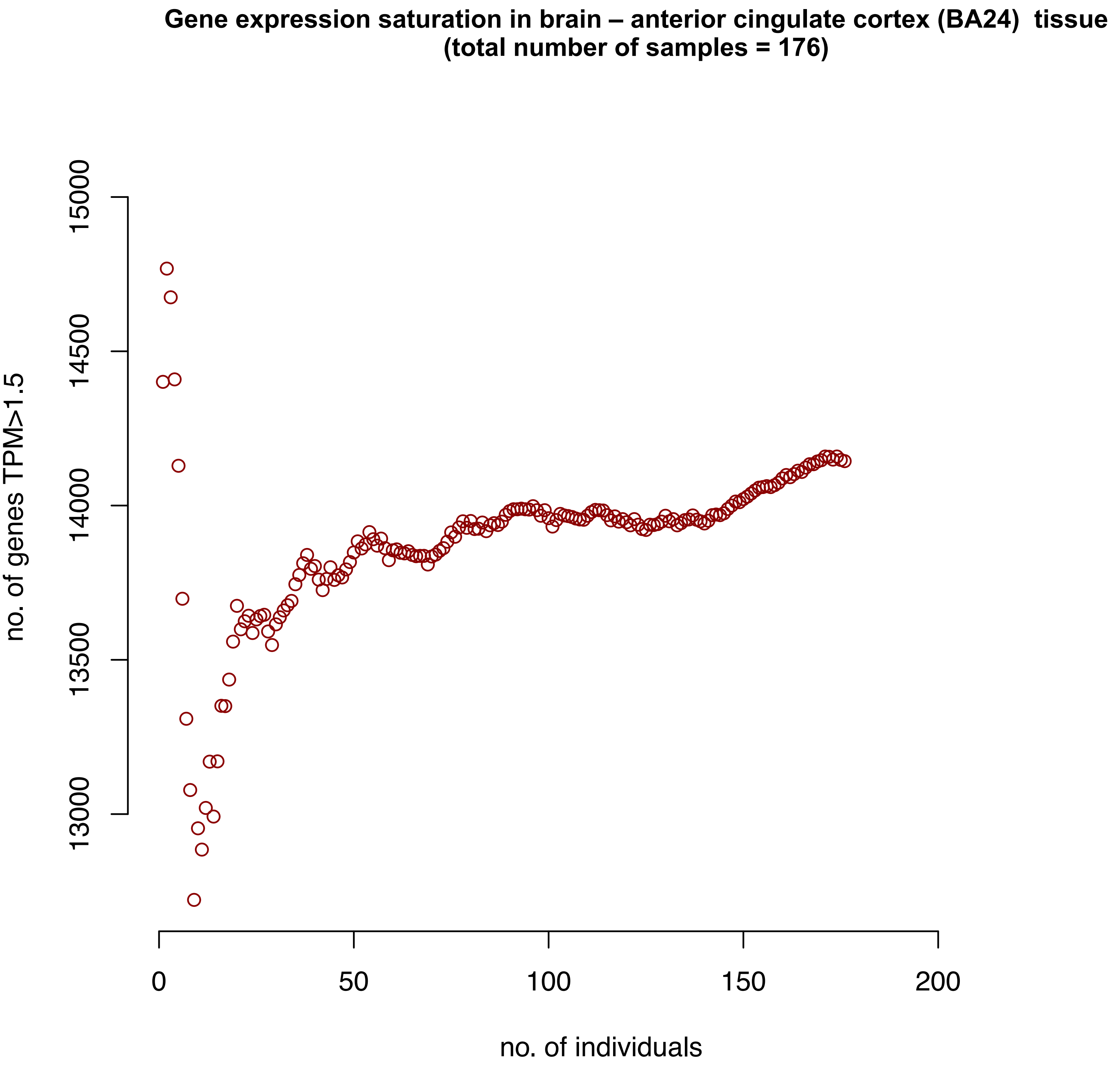

Gene expression saturation in brain – anterior cingulate cortex (BA24) tissue
(total number of samples = 176)
no. of genes TPM>1.5
15000
14500
14000
13500
13000
0
50
100
150
200
no. of individuals

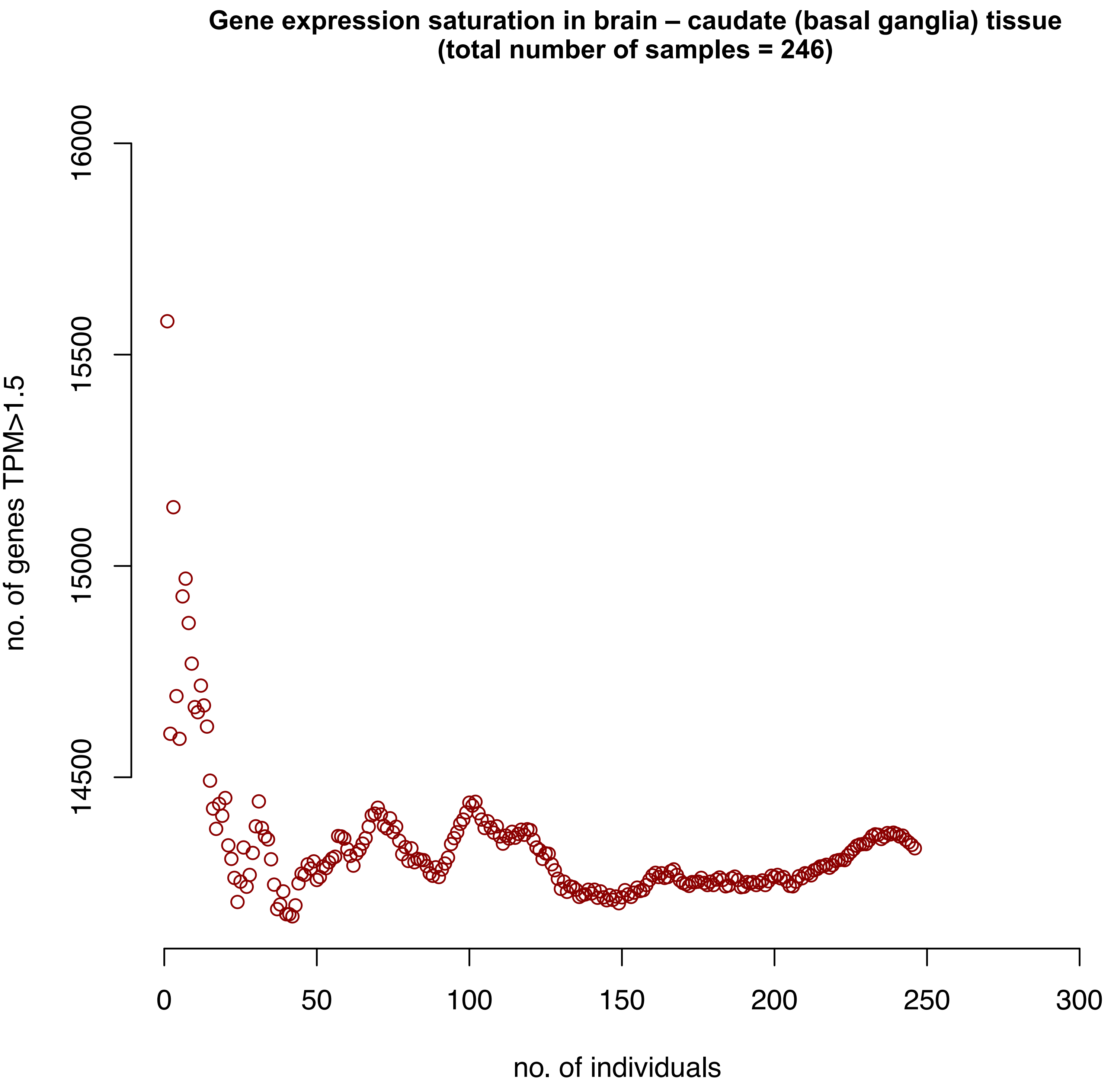

Gene expression saturation in brain – caudate (basal ganglia) tissue
(total number of samples = 246)
no. of genes TPM>1.5
16000
15500
15000
14500
0
50
100
150
200
250
300
no. of individuals

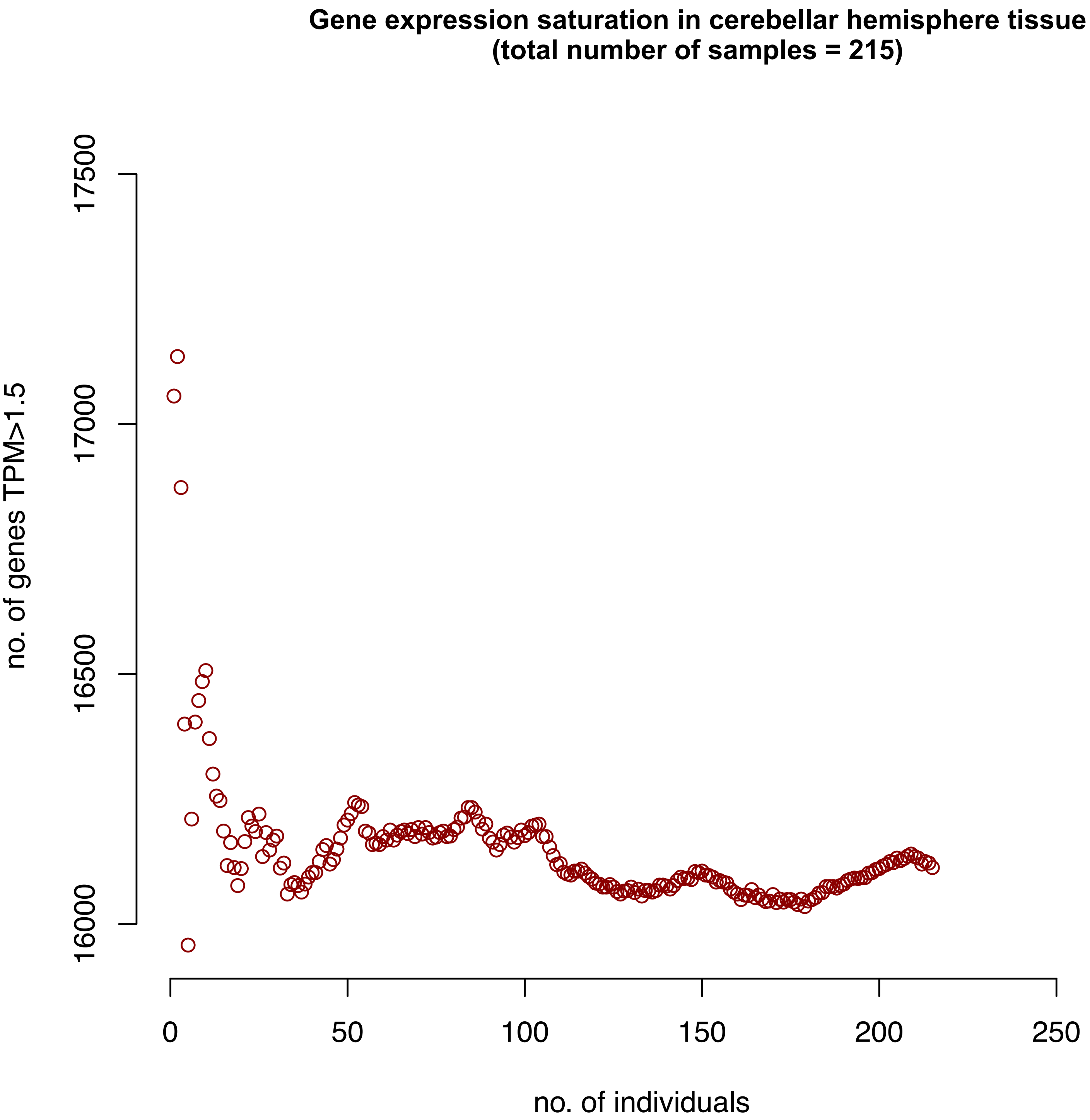
Gene expression saturation in cerebellar hemisphere tissue
(total number of samples = 215)
no. of genes TPM>1.5
16000
16500
17000
17500
0
50
100
150
200
250
no. of individuals

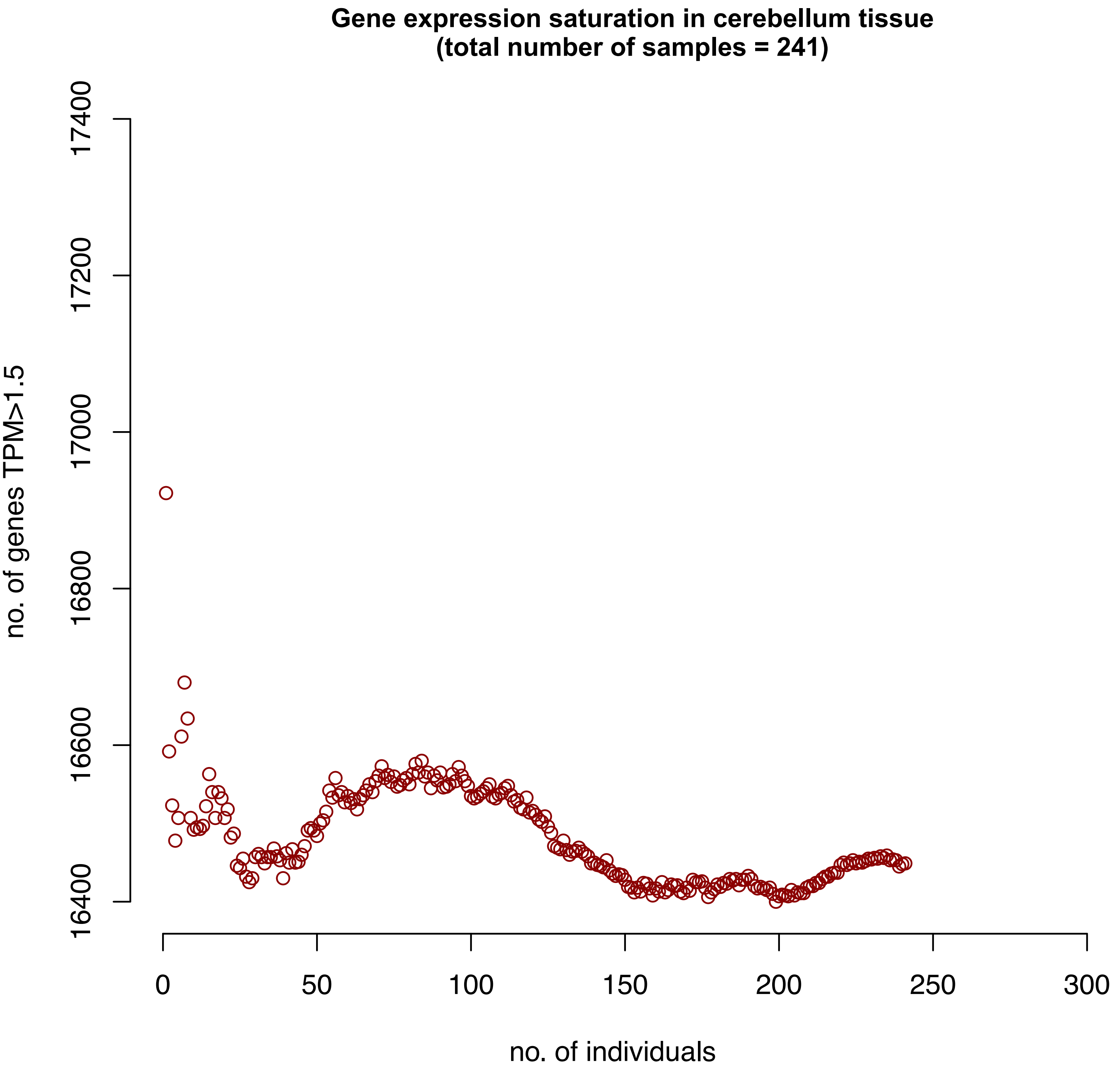

Gene expression saturation in cerebellum tissue
(total number of samples = 241)
17400
17200
17000
16800
16600
16400
no. of genes TPM>1.5
0
50
100
150
200
250
300
no. of individuals

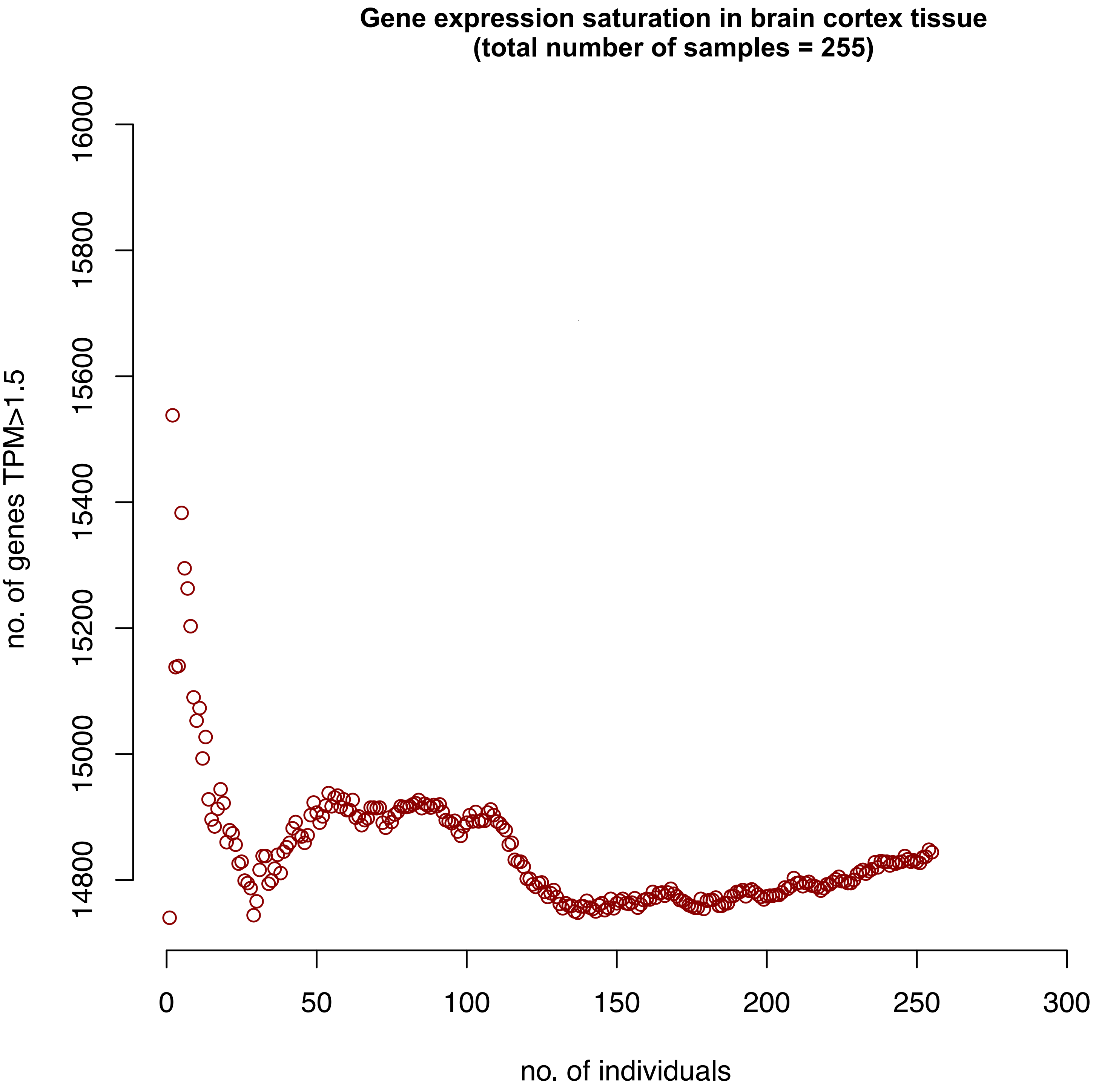
Gene expression saturation in brain cortex tissue
(total number of samples = 255)
no. of genes TPM>1.5
16000
15800
15600
15400
15200
15000
14800
0
50
100
150
200
250
300
no. of individuals

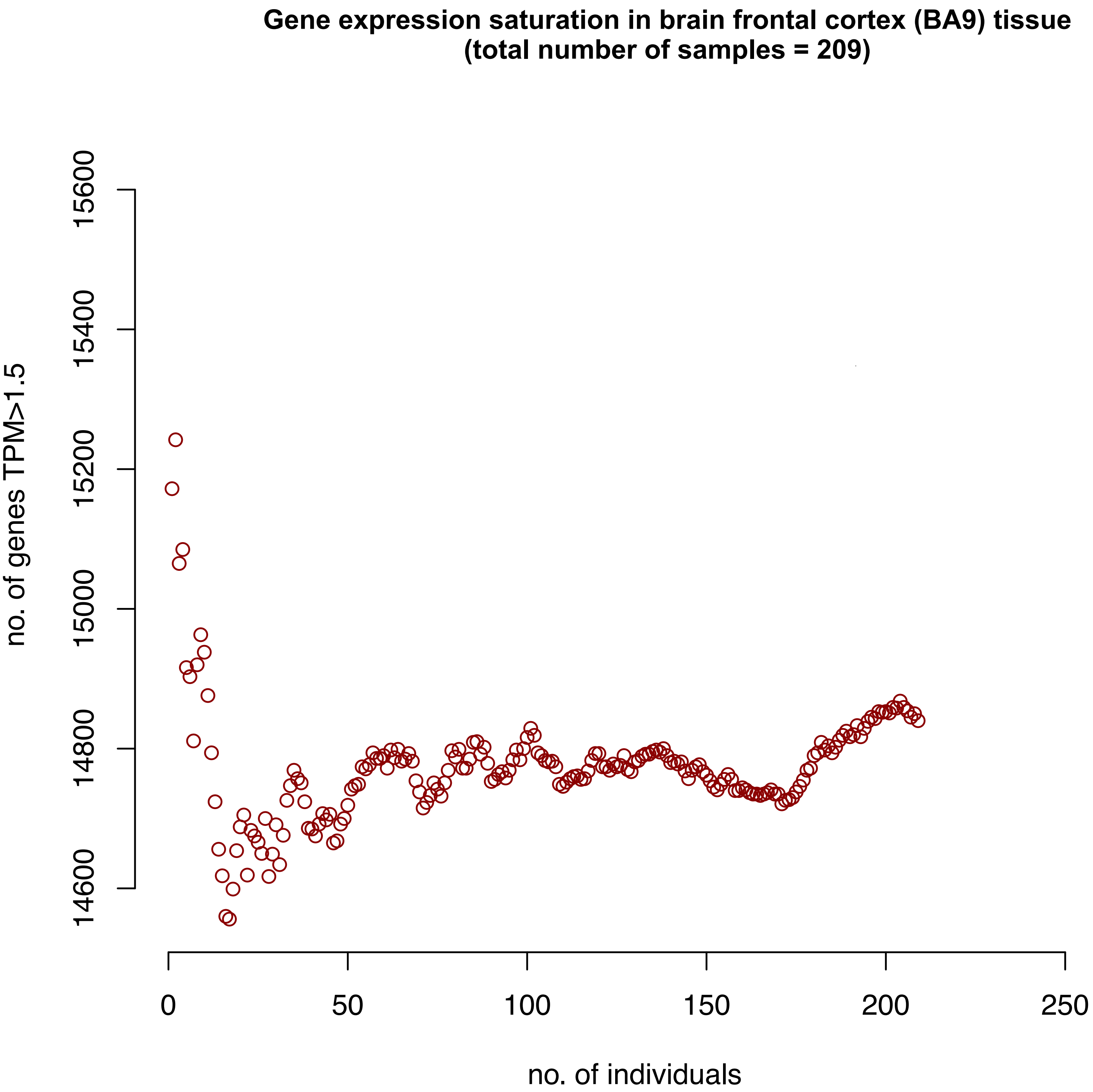
Gene expression saturation in brain frontal cortex (BA9) tissue
(total number of samples = 209)
no. of genes TPM>1.5
14600
14800
15000
15200
15400
15600
0
50
100
150
200
250
no. of individuals

Gene expression saturation in brain hippocampus tissue
(total number of samples = 197)

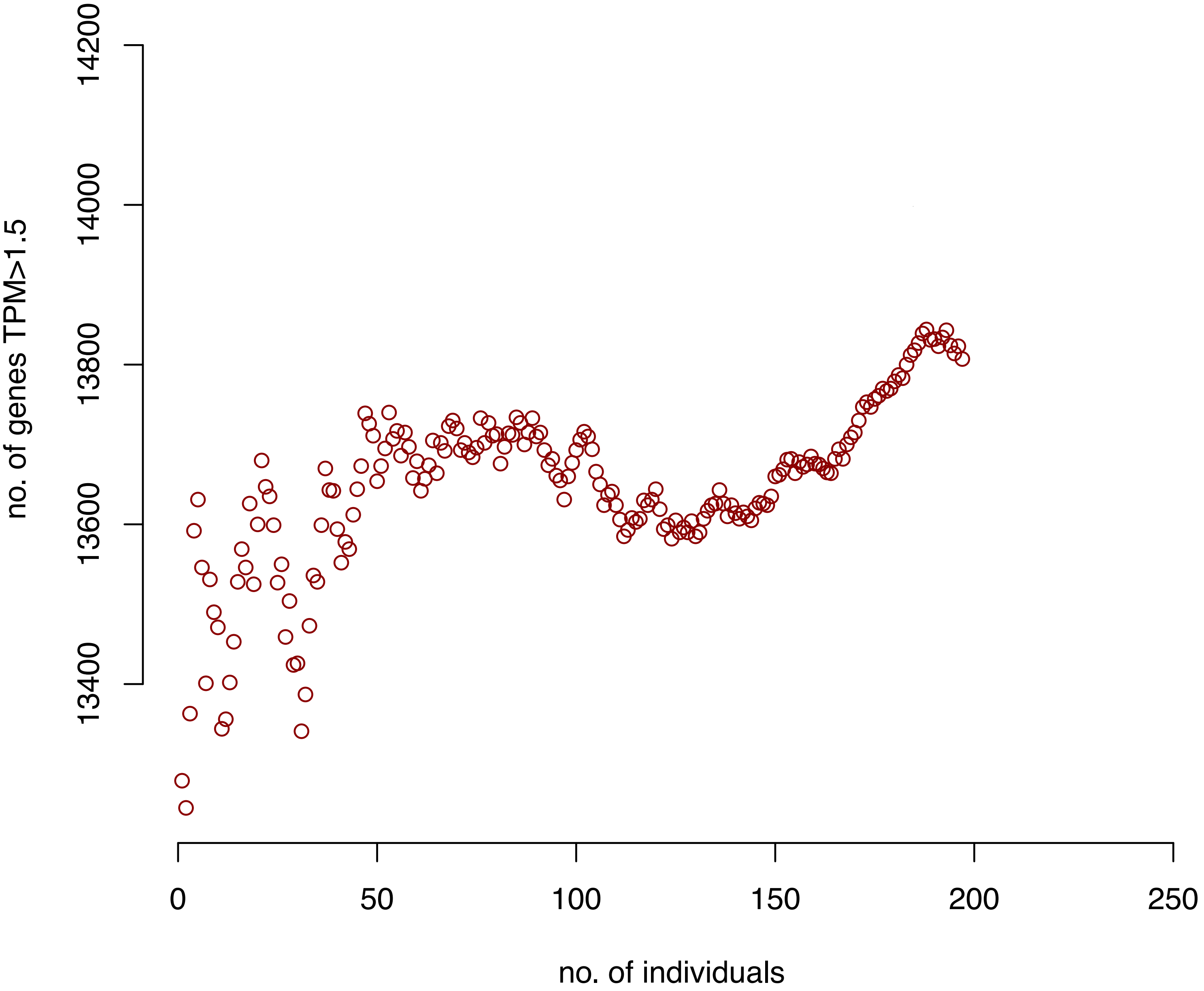

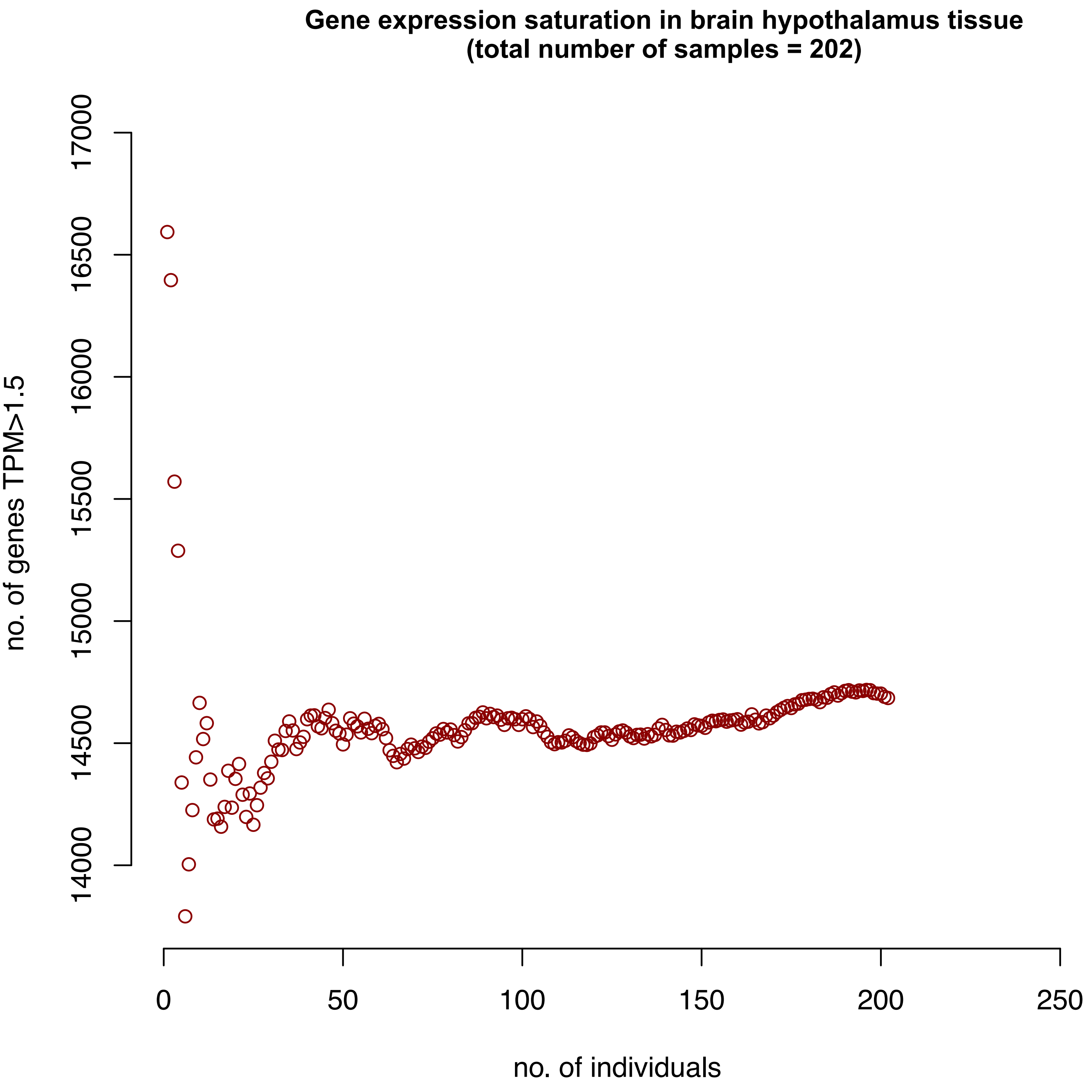
Gene expression saturation in brain hypothalamus tissue
(total number of samples = 202)
no. of genes TPM>1.5
14000
14500
15000
15500
16000
16500
17000
0
50
100
150
200
250
no. of individuals

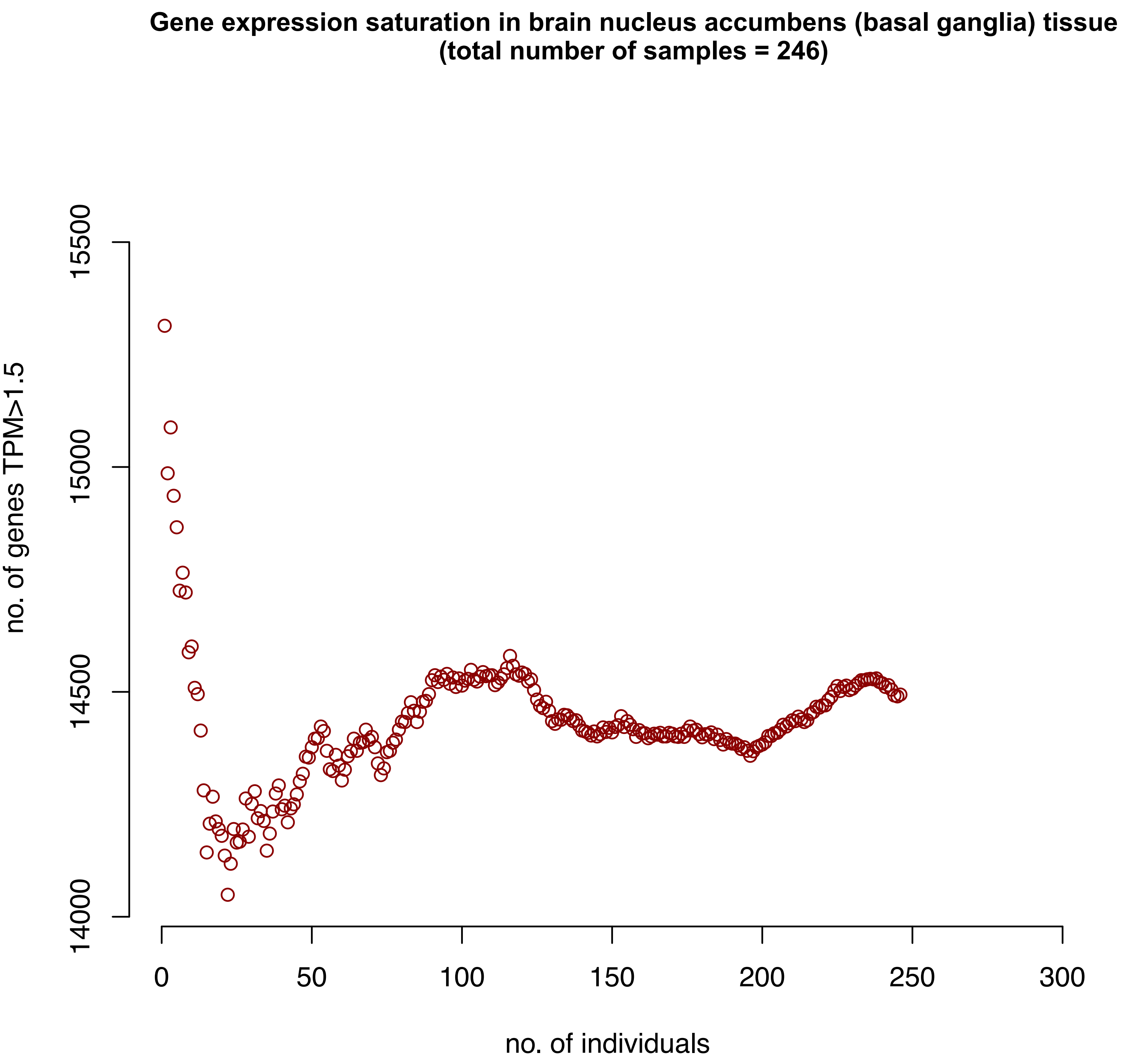
Gene expression saturation in brain nucleus accumbens (basal ganglia) tissue
(total number of samples = 246)
no. of genes TPM>1.5
14000
14500
15000
15500
0
50
100
150
200
250
300
no. of individuals

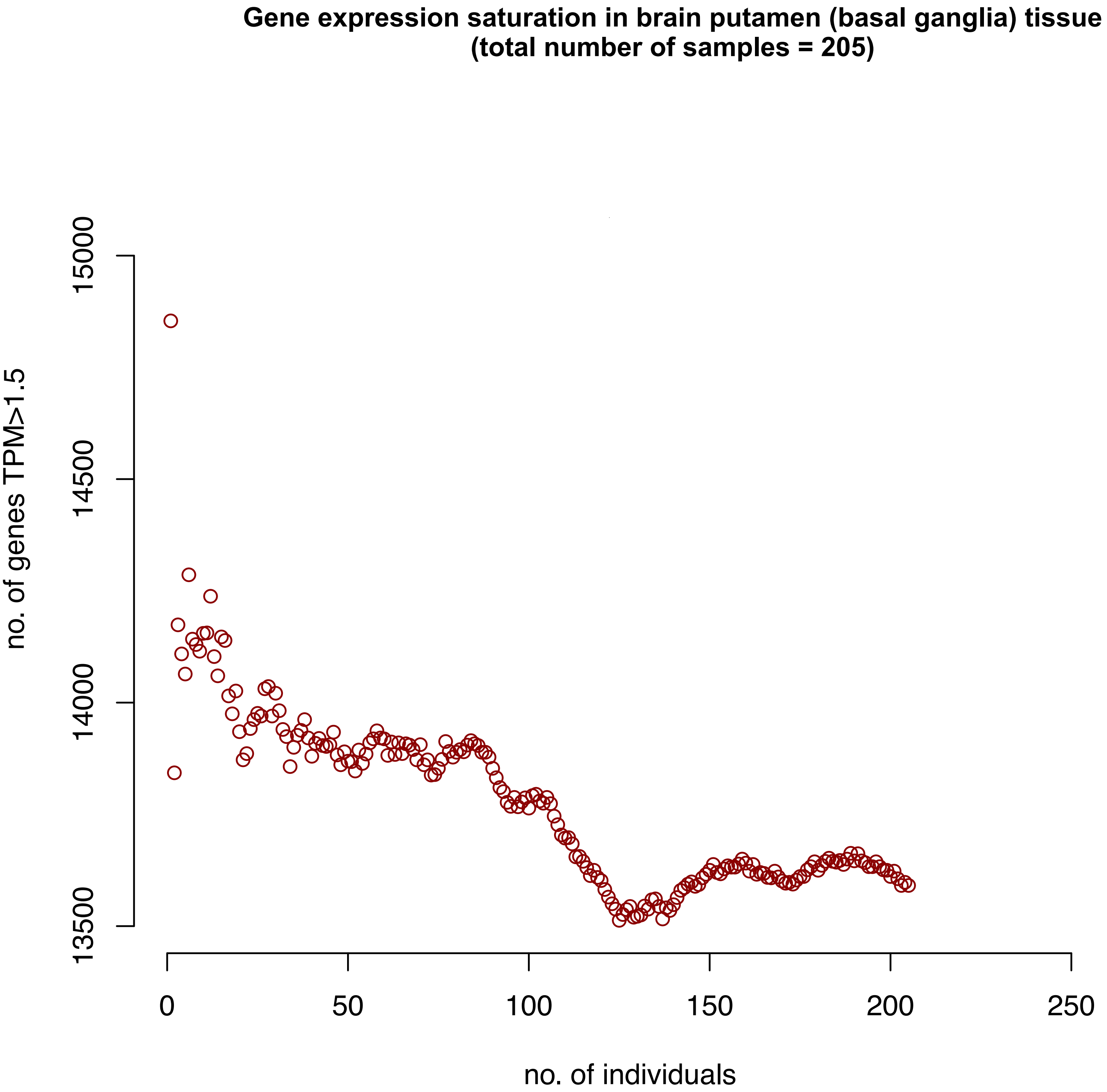
Gene expression saturation in brain putamen (basal ganglia) tissue
(total number of samples = 205)
no. of genes TPM>1.5
15000
14500
14000
13500
0
50
100
150
200
250
no. of individuals

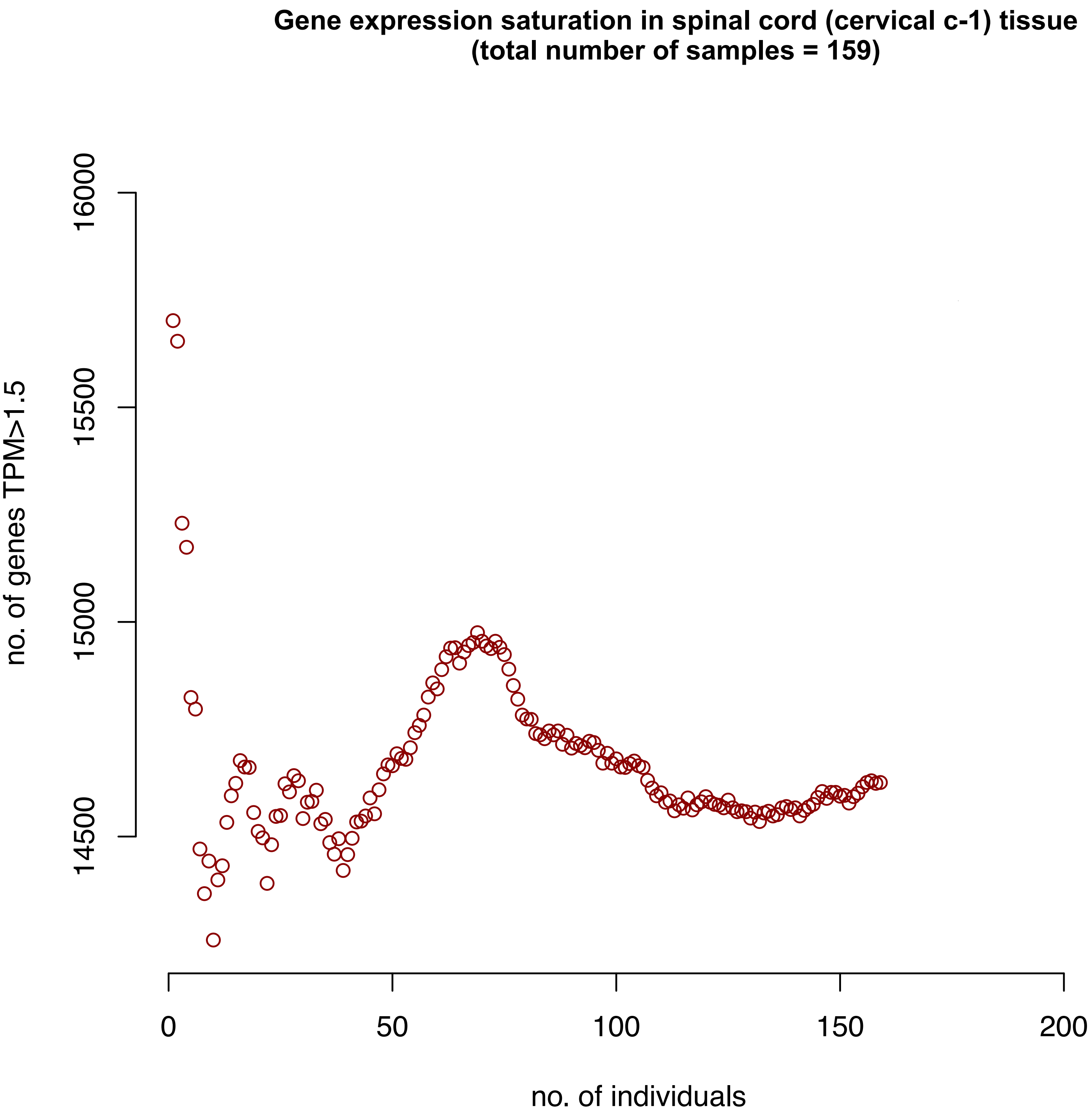
Gene expression saturation in spinal cord (cervical c-1) tissue
(total number of samples = 159)
no. of genes TPM>1.5
14500
15000
15500
16000
0
50
100
150
200
no. of individuals

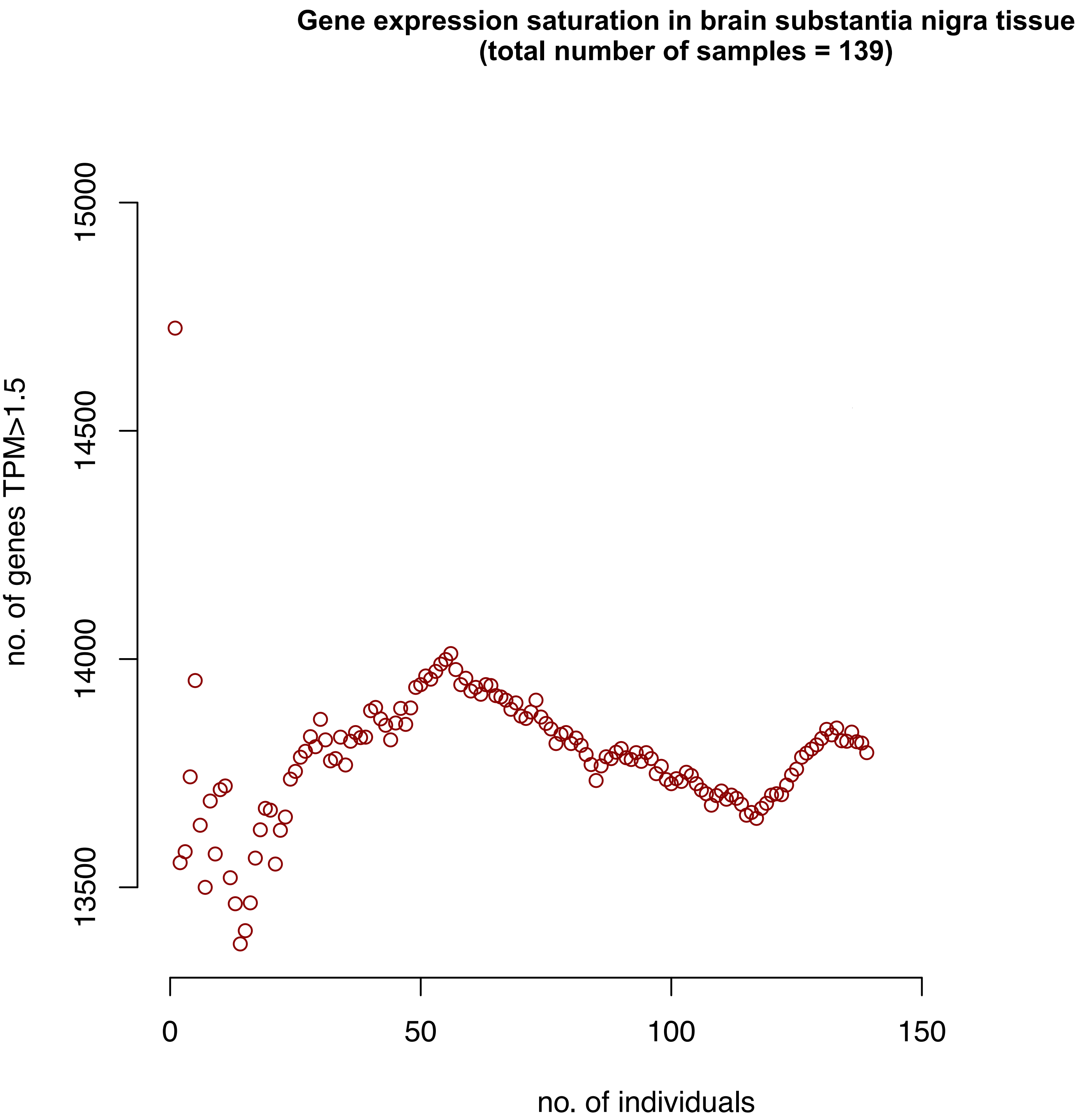
Gene expression saturation in brain substantia nigra tissue
(total number of samples = 139)
no. of genes TPM>1.5
13500
14000
14500
15000
0
50
100
150
no. of individuals

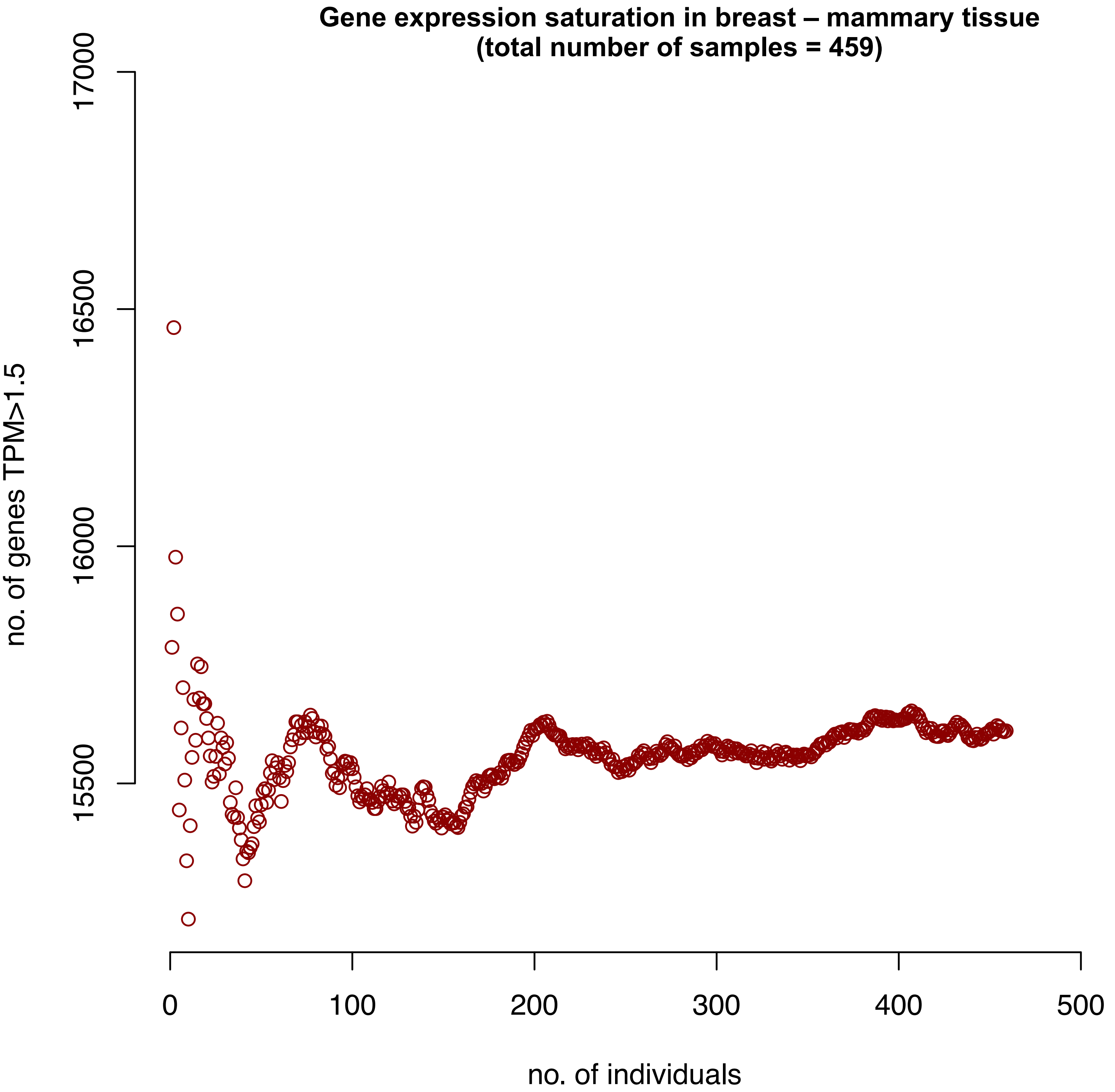
Gene expression saturation in breast – mammary tissue
(total number of samples = 459)
no. of genes TPM>1.5
no. of individuals
15500
16000
16500
17000
0
100
200
300
400
500

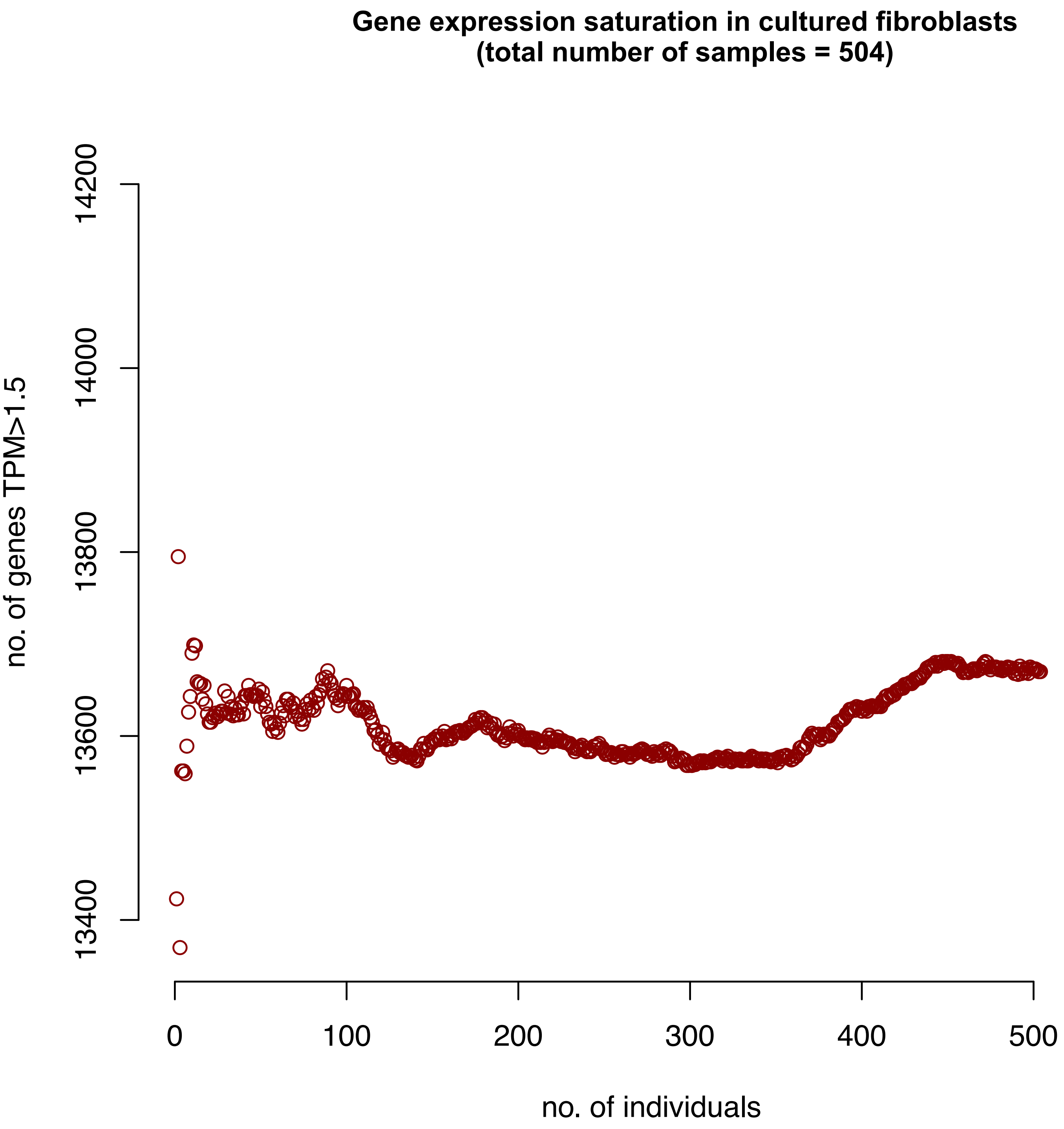
Gene expression saturation in cultured fibroblasts
(total number of samples = 504)
no. of genes TPM>1.5
14200
14000
13800
13600
13400
0
100
200
300
400
500
no. of individuals

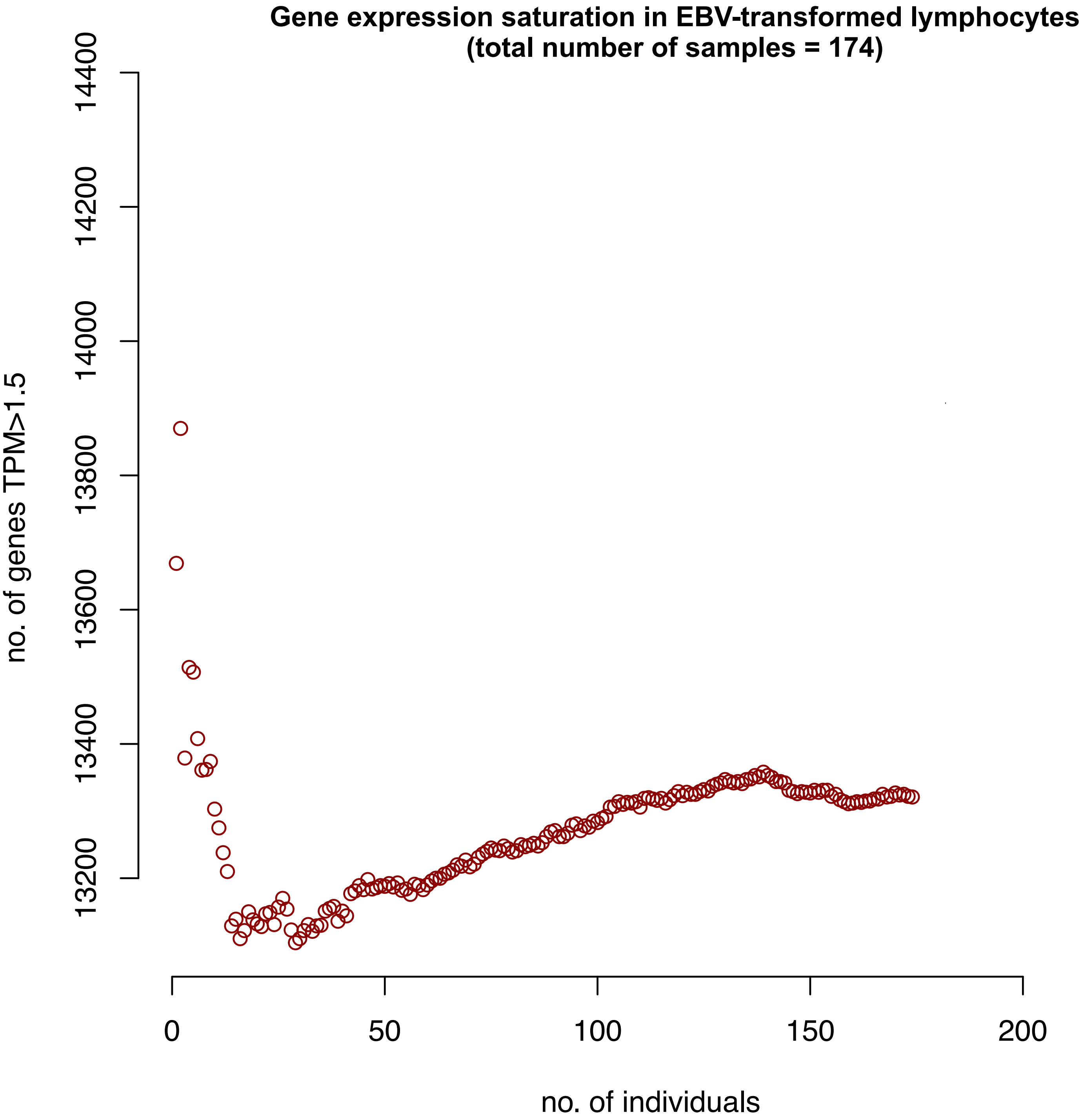
Gene expression saturation in EBV-transformed lymphocytes
(total number of samples = 174)
no. of genes TPM>1.5
13200
13400
13600
13800
14000
14200
14400
0
50
100
150
200
no. of individuals

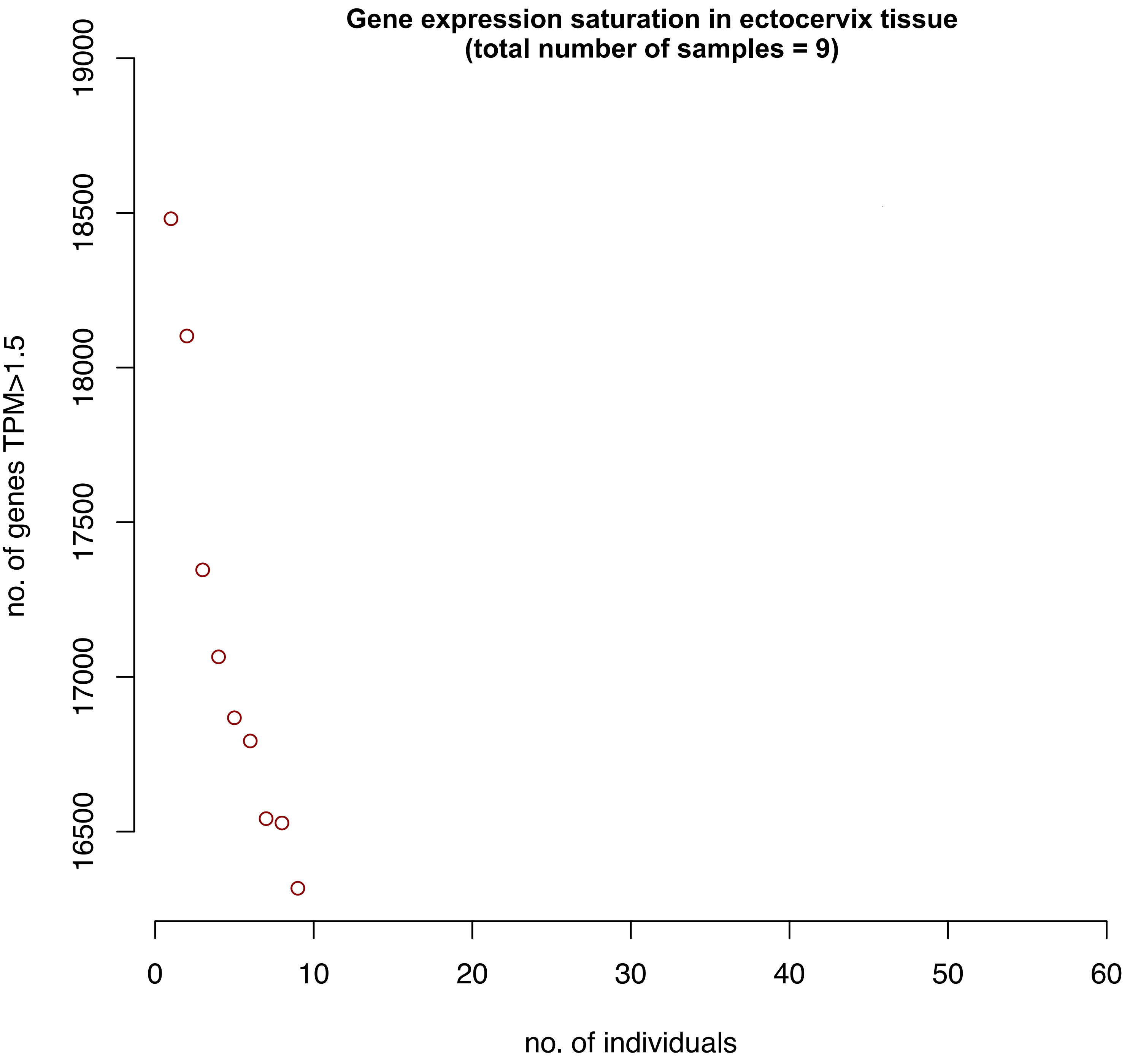
Gene expression saturation in ectocervix tissue
(total number of samples = 9)
no. of genes TPM>1.5
19000
18500
18000
17500
17000
16500
0
10
20
30
40
50
60
no. of individuals

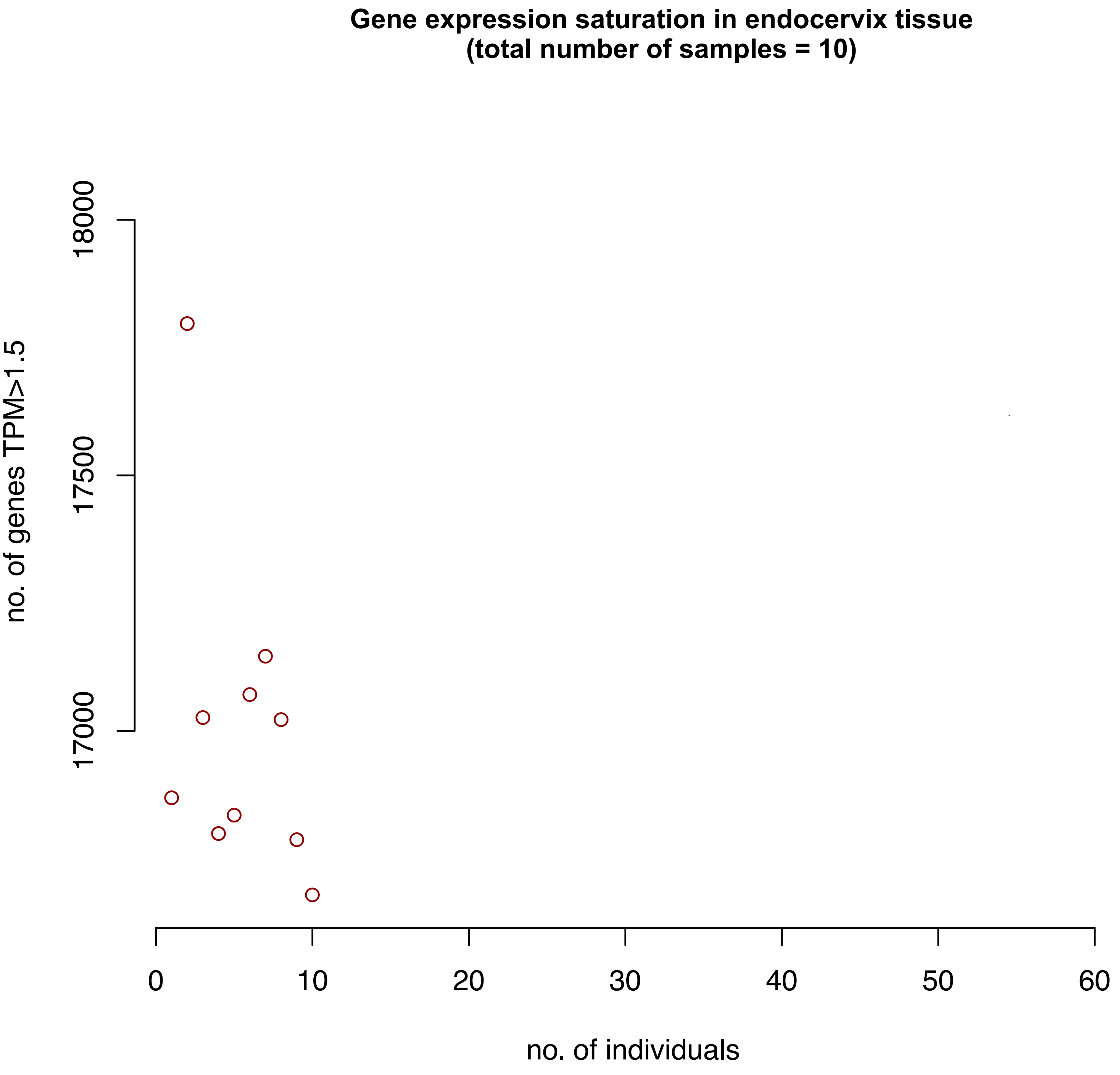

Gene expression saturation in endocervix tissue
(total number of samples = 10)
no. of genes TPM>1.5
17000
17500
18000
0
10
20
30
40
50
60
no. of individuals

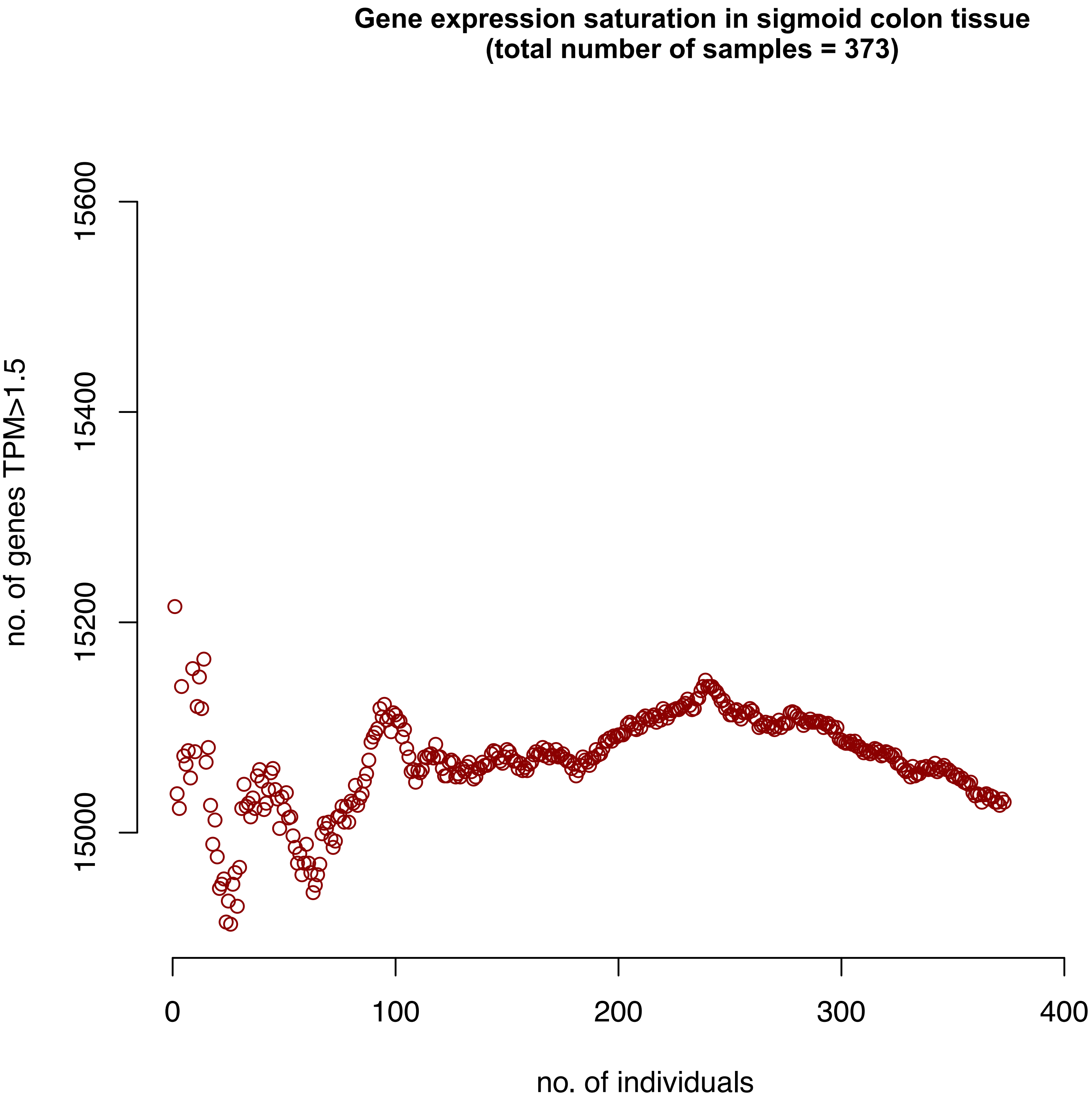
Gene expression saturation in sigmoid colon tissue
(total number of samples = 373)
no. of genes TPM>1.5
15000
15200
15400
15600
no. of individuals
0
100
200
300
400

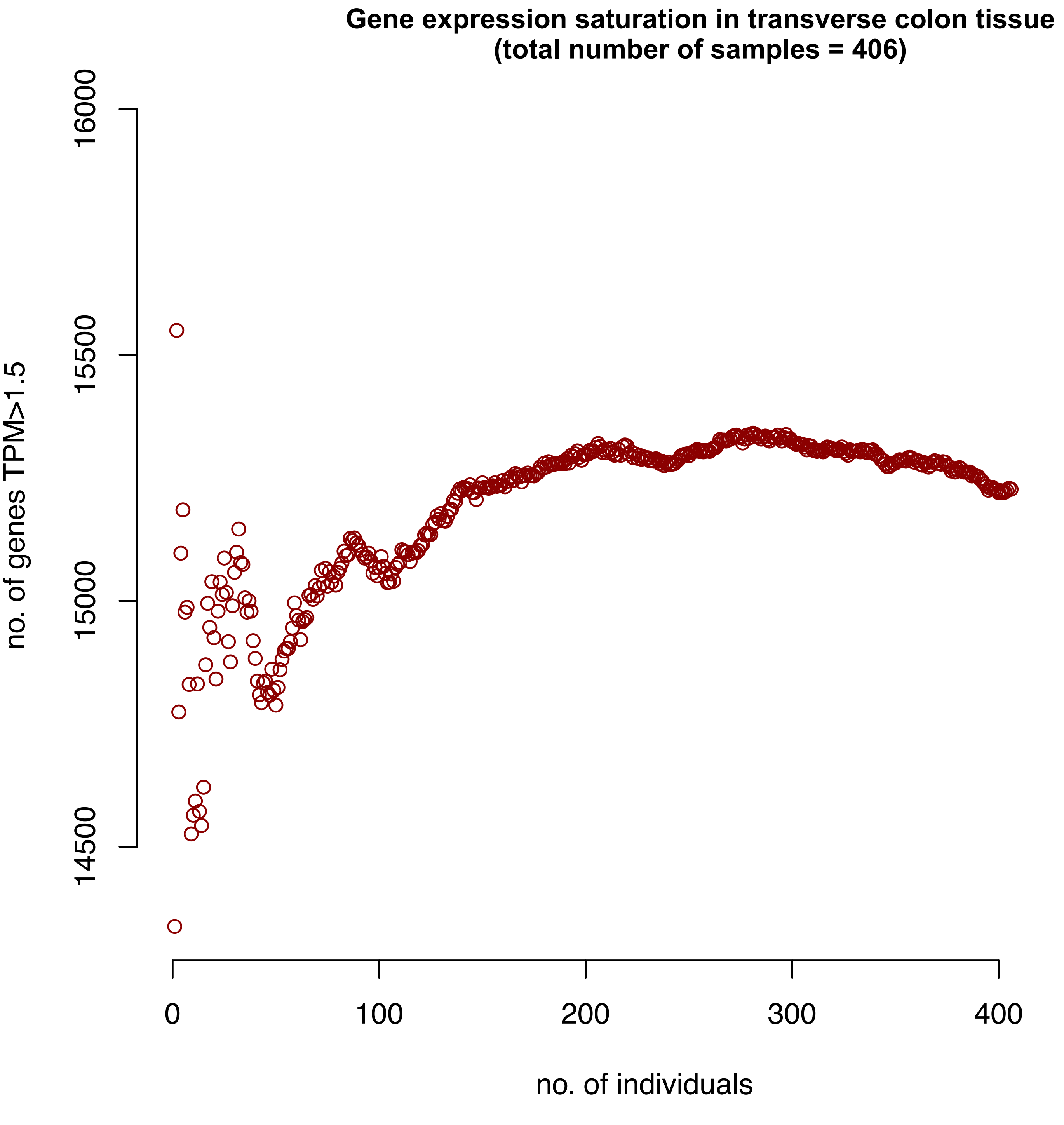
Gene expression saturation in transverse colon tissue
(total number of samples = 406)
no. of genes TPM>1.5
16000
15500
15000
14500
0
100
200
300
400
no. of individuals

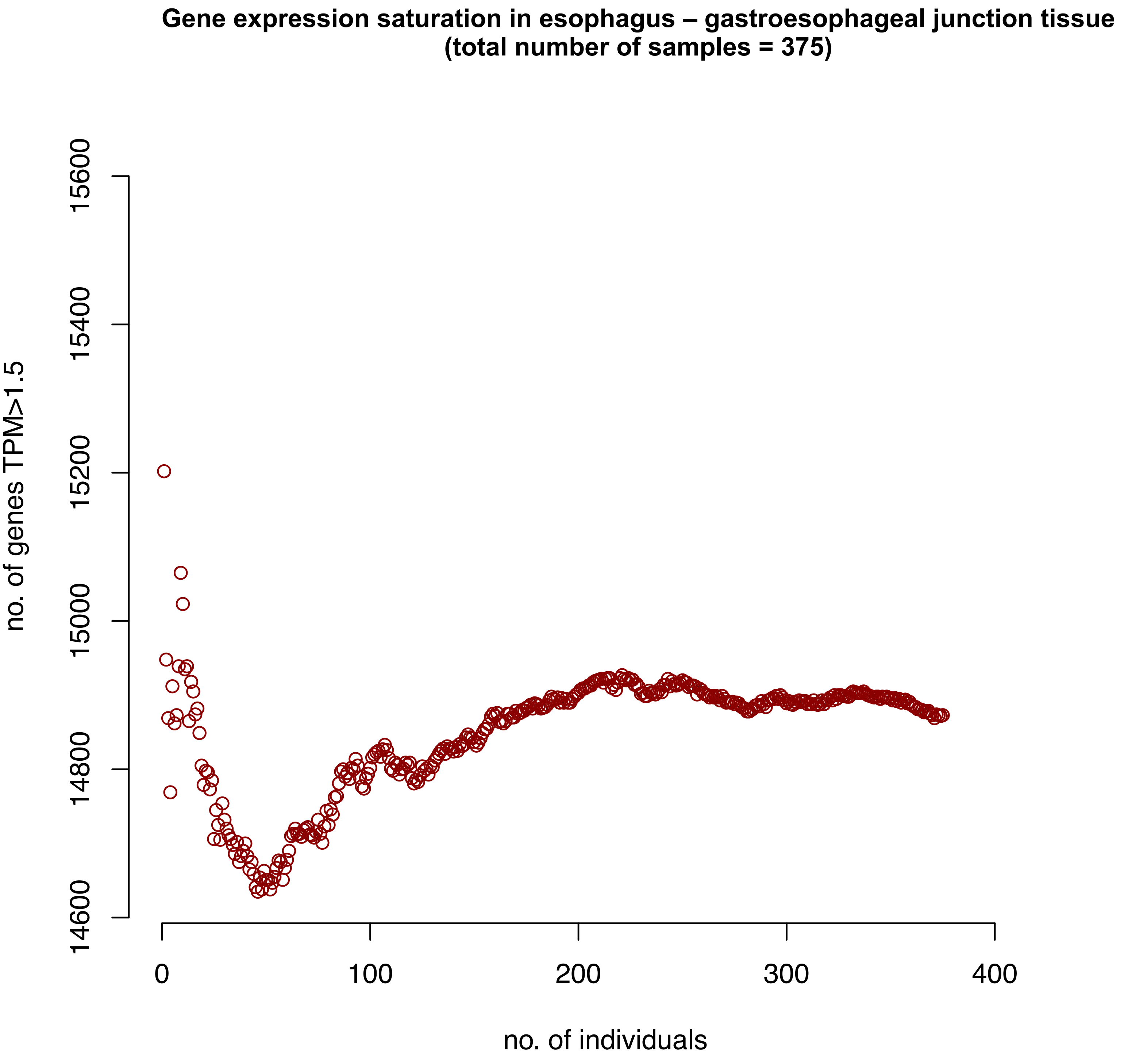
Gene expression saturation in esophagus – gastroesophageal junction tissue
(total number of samples = 375)
no. of genes TPM>1.5
15600
15400
15200
15000
14800
14600
0
100
200
300
400
no. of individuals

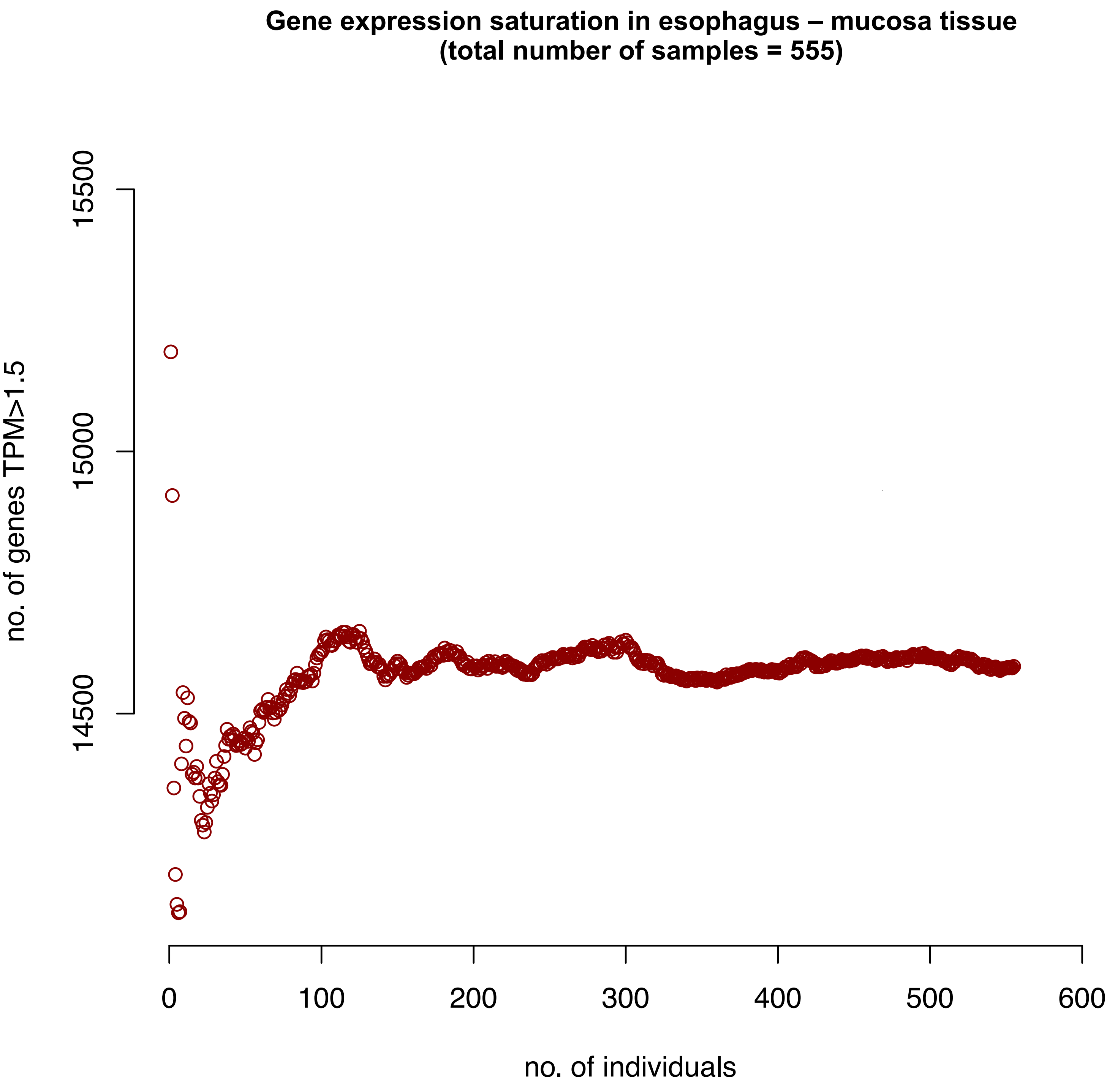
Gene expression saturation in esophagus – mucosa tissue
(total number of samples = 555)
no. of genes TPM>1.5
15500
15000
14500
0
100
200
300
400
500
600
no. of individuals

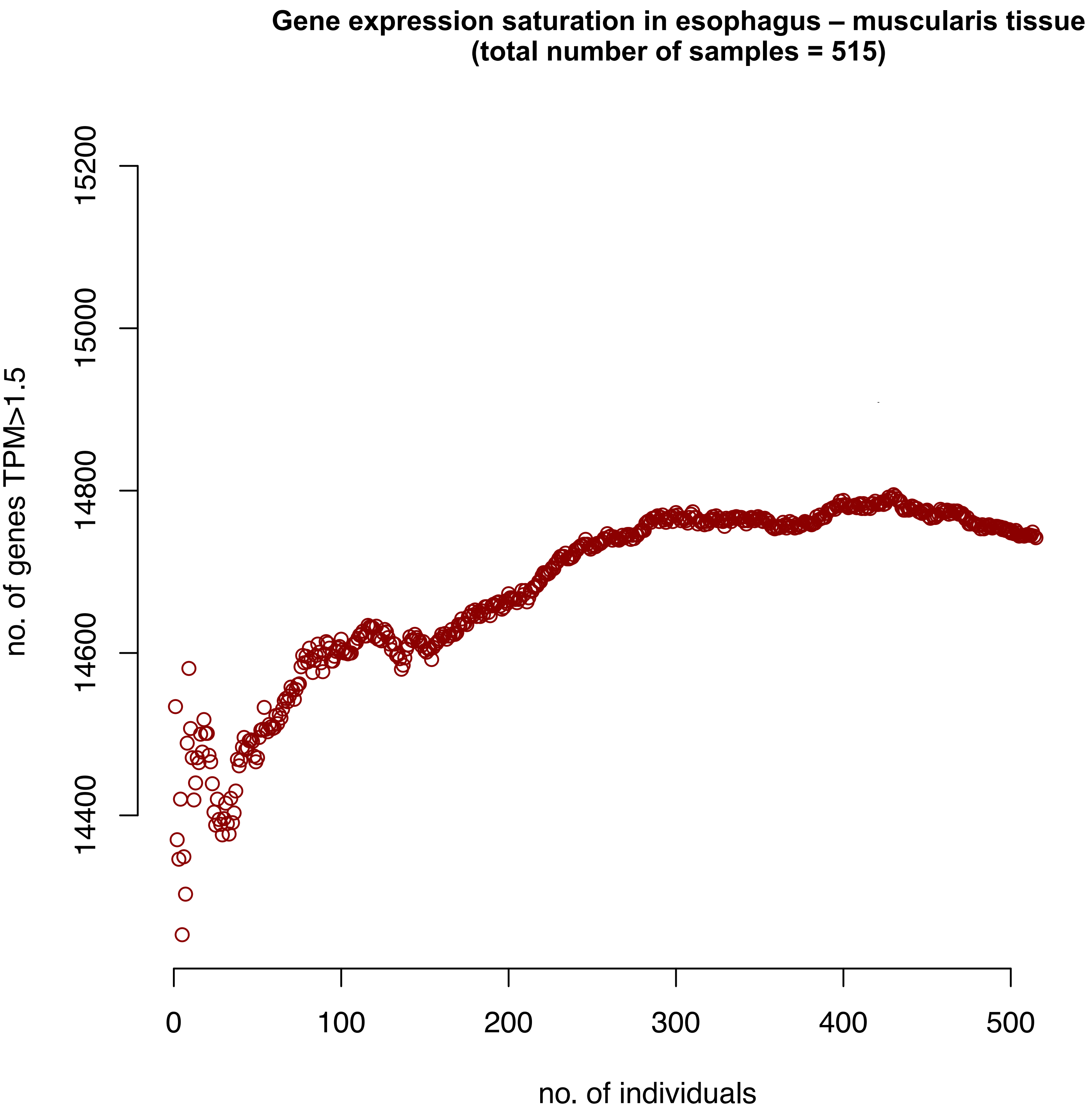
Gene expression saturation in esophagus – muscularis tissue
(total number of samples = 515)
no. of genes TPM>1.5
15200
15000
14800
14600
14400
0
100
200
300
400
500
no. of individuals

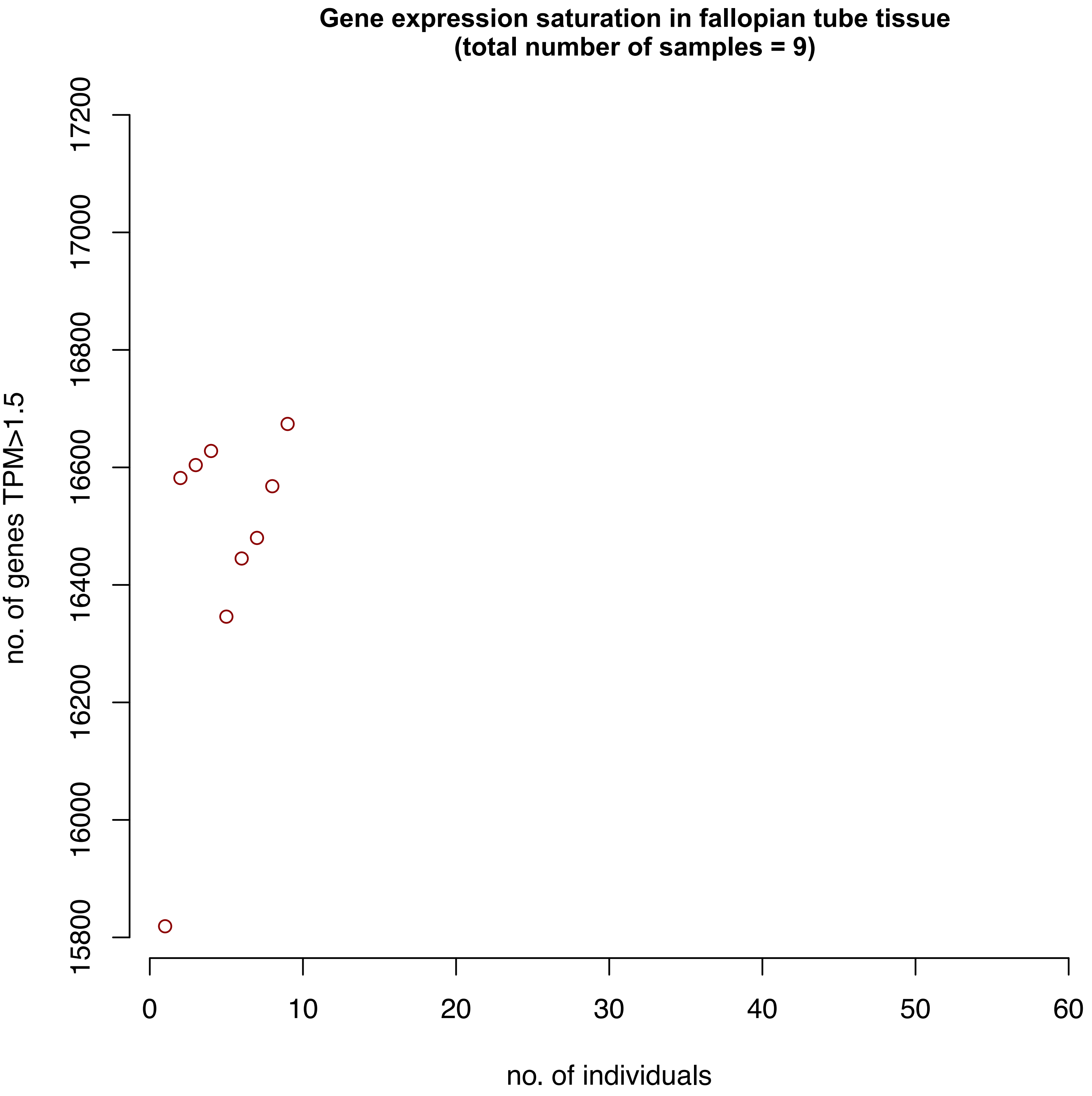
Gene expression saturation in fallopian tube tissue
(total number of samples = 9)
no. of genes TPM>1.5
15800
16000
16200
16400
16600
16800
17000
17200
0
10
20
30
40
50
60
no. of individuals

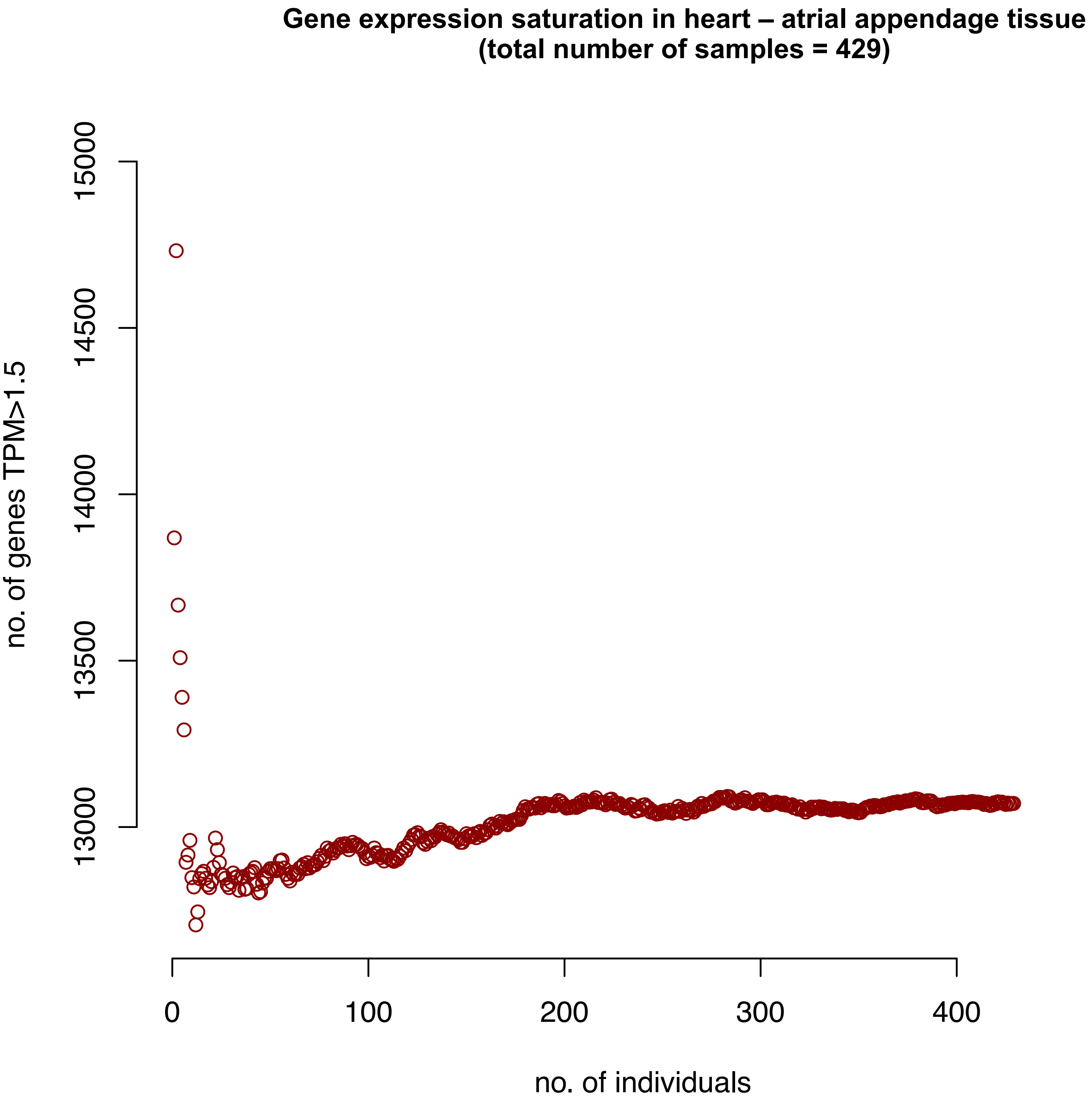
Gene expression saturation in heart – atrial appendage tissue
(total number of samples = 429)
no. of genes TPM>1.5
15000
14500
14000
13500
13000
0
100
200
300
400
no. of individuals

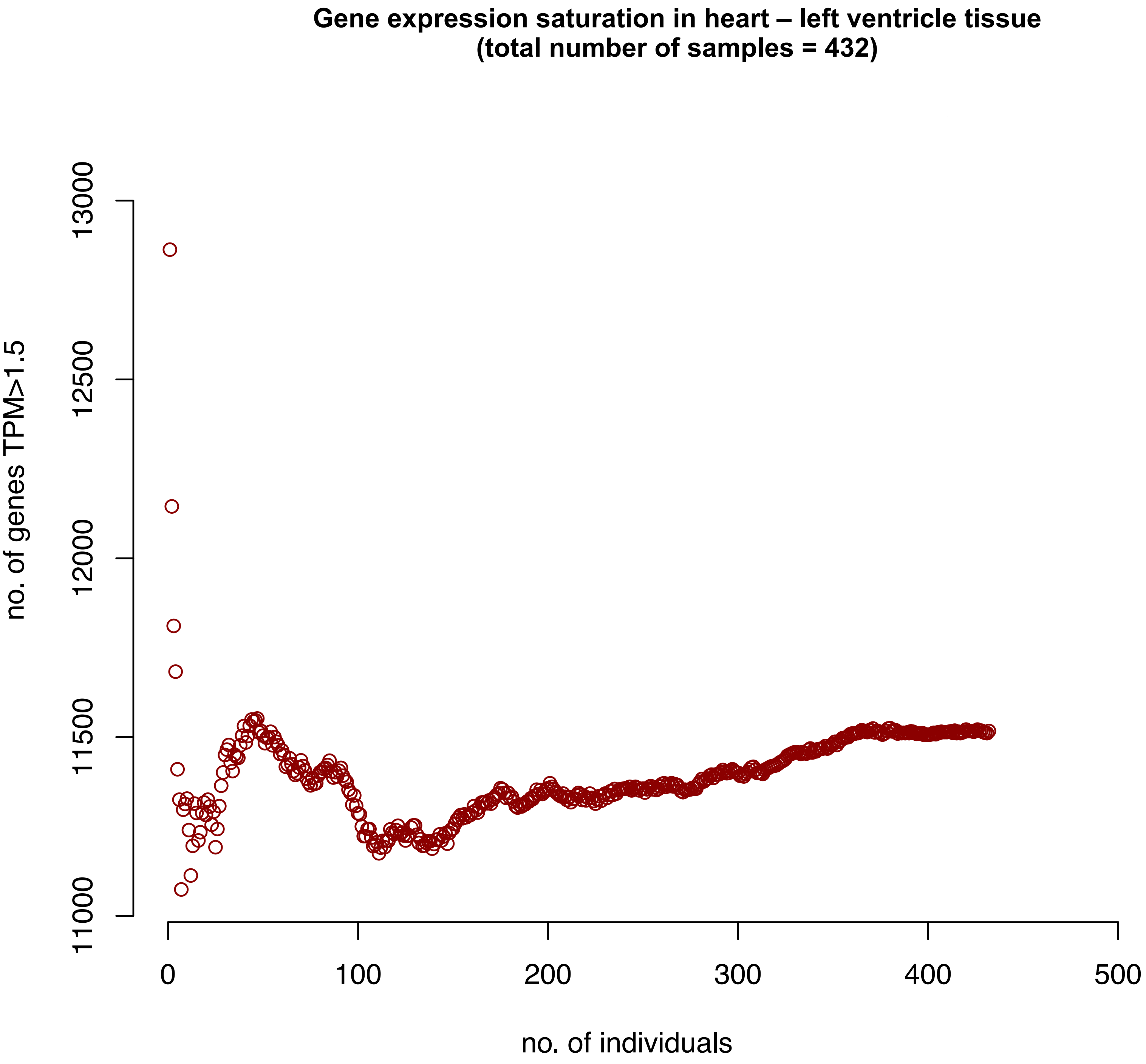

Gene expression saturation in heart – left ventricle tissue
(total number of samples = 432)
no. of genes TPM>1.5
13000
12500
12000
11500
11000
0
100
200
300
400
500
no. of individuals

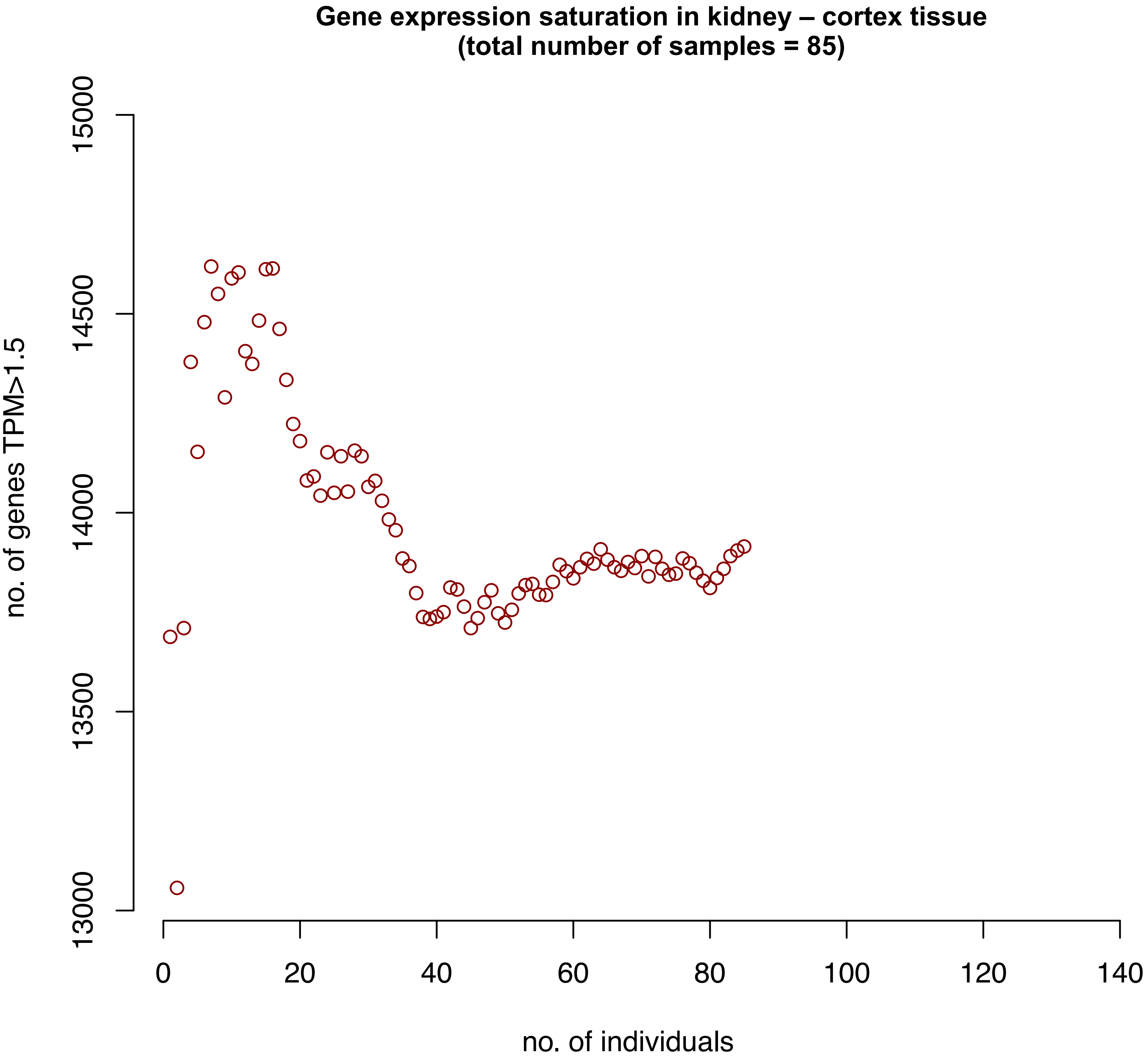
Gene expression saturation in kidney – cortex tissue
(total number of samples = 85)
no. of genes TPM>1.5
15000
14500
14000
13500
13000
0
20
40
60
80
100
120
140
no. of individuals

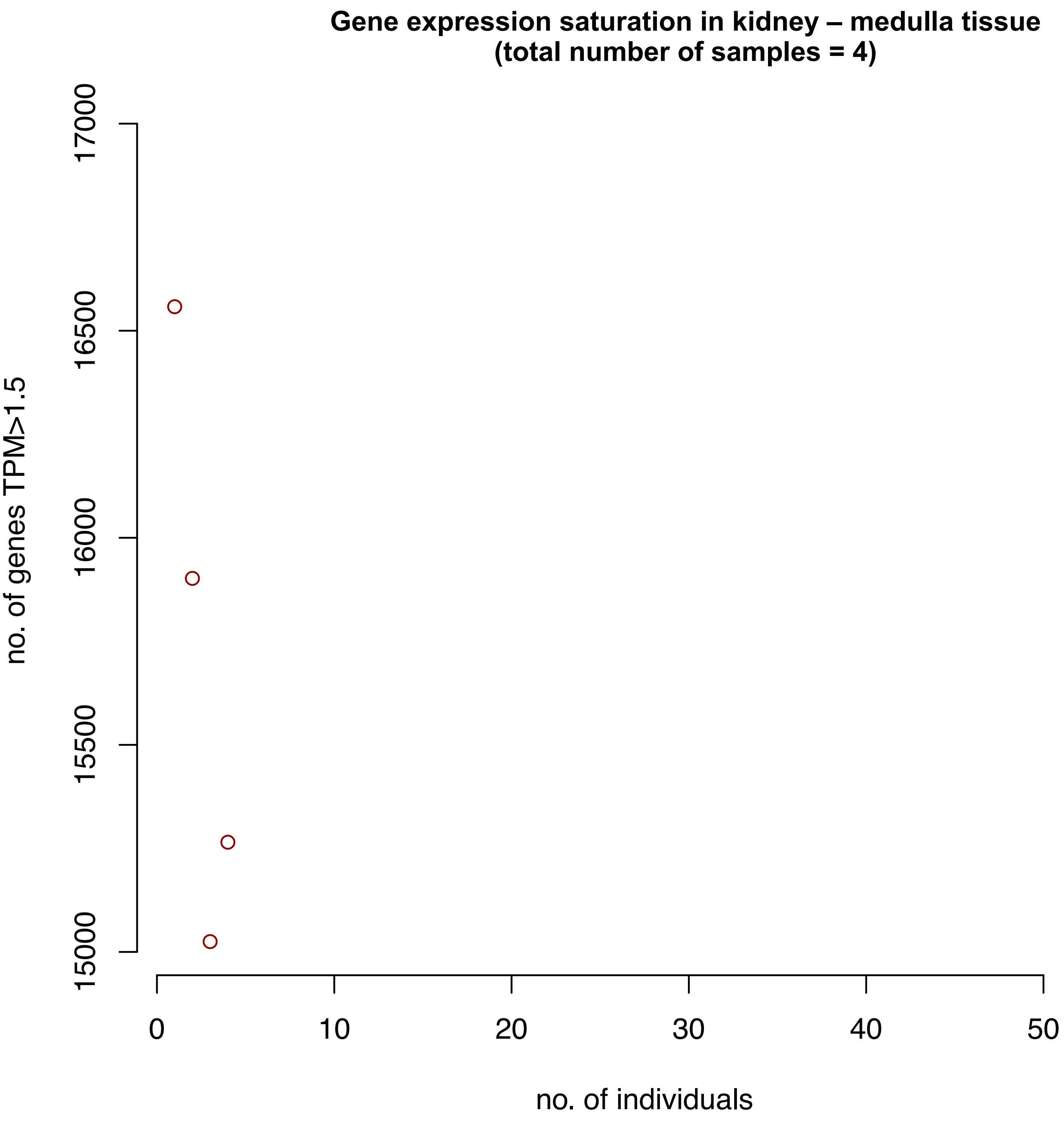

Gene expression saturation in kidney – medulla tissue
(total number of samples = 4)
no. of genes TPM>1.5
17000
16500
16000
15500
15000
0
10
20
30
40
50
no. of individuals

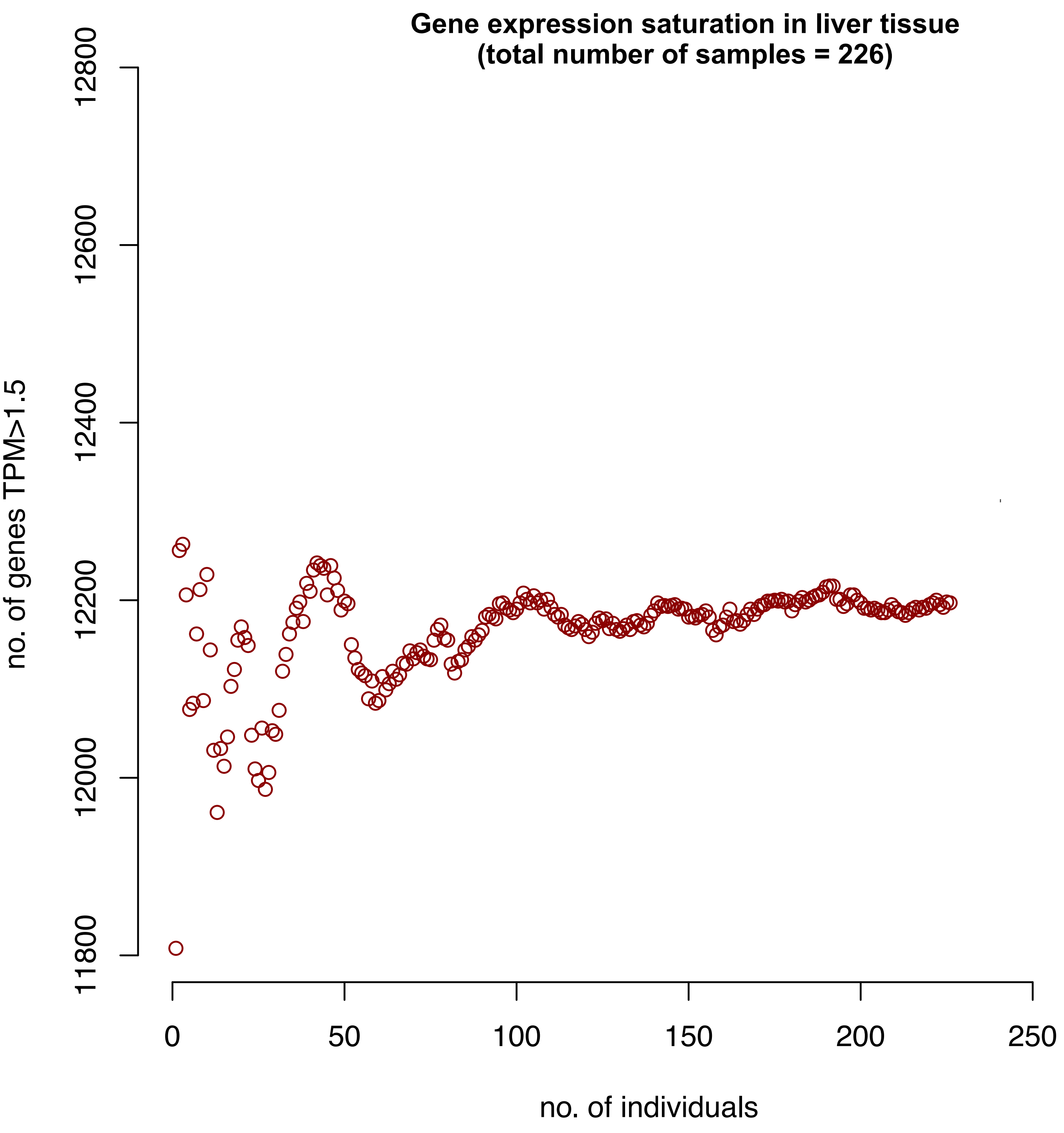

Gene expression saturation in liver tissue
(total number of samples = 226)
no. of genes TPM>1.5
no. of individuals
11800
12000
12200
12400
12600
12800
0
50
100
150
200
250

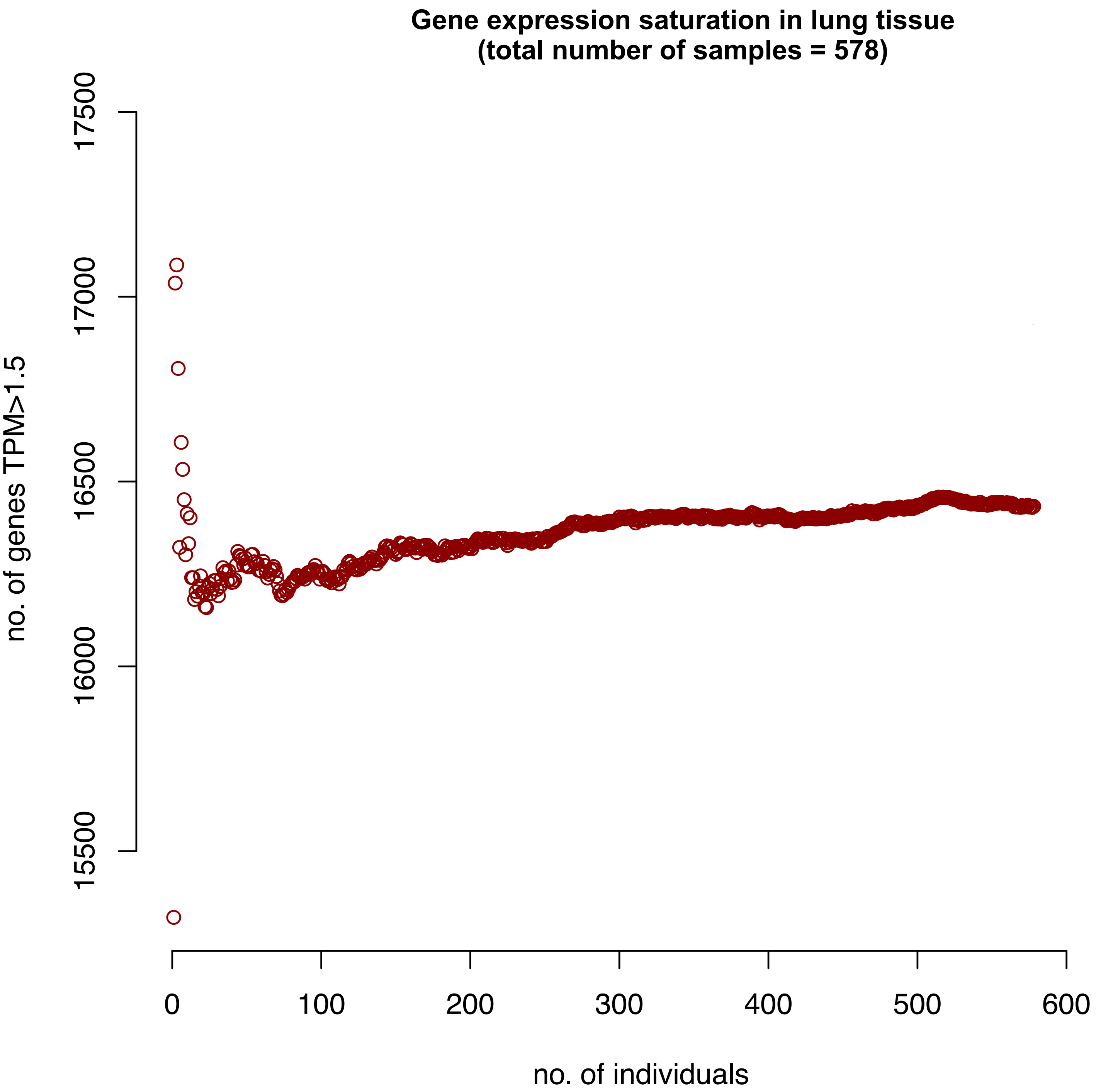
Gene expression saturation in lung tissue
(total number of samples = 578)
no. of genes TPM>1.5
no. of individuals
17500
17000
16500
16000
15500
0
100
200
300
400
500
600

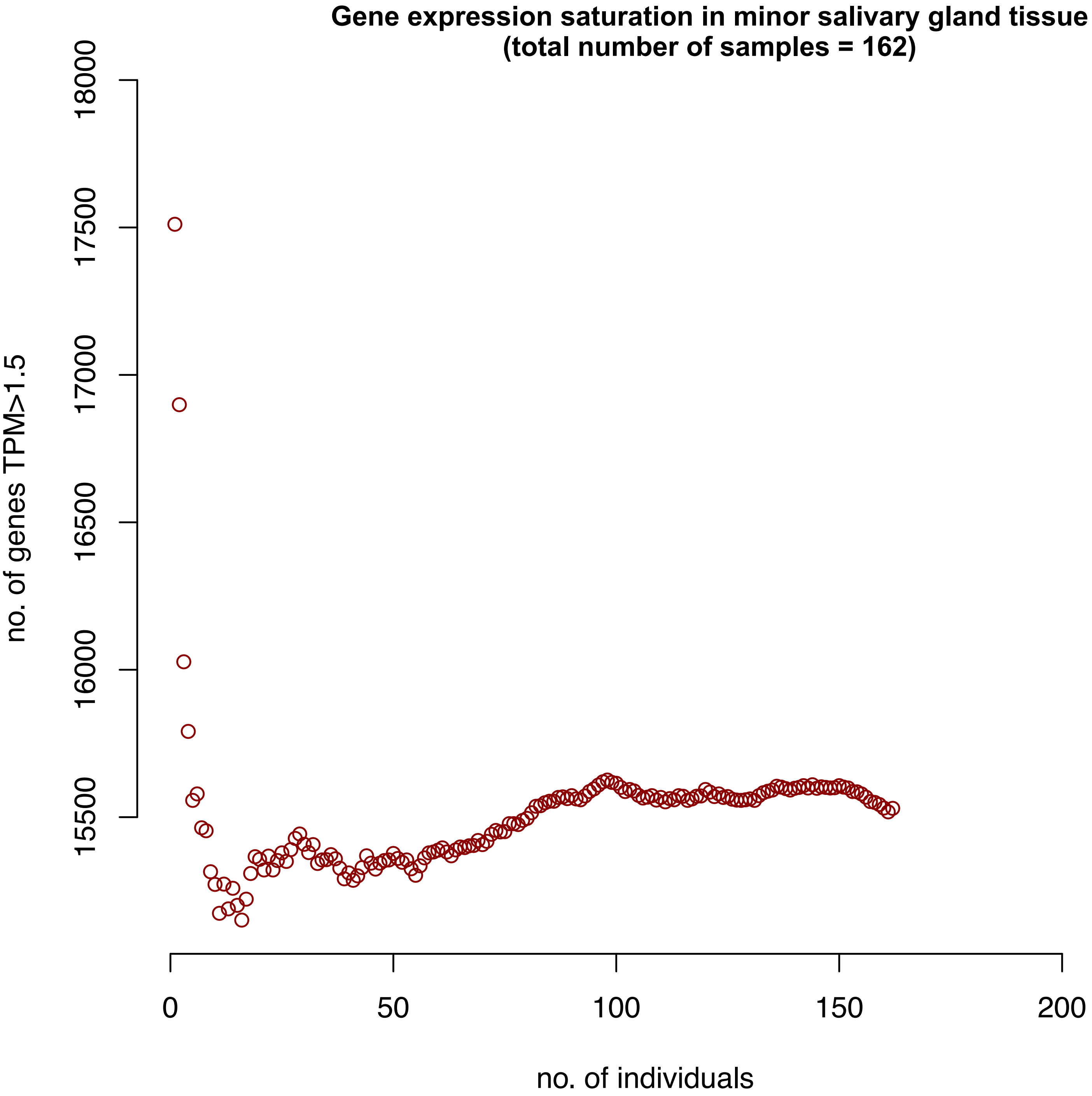
Gene expression saturation in minor salivary gland tissue
(total number of samples = 162)
no. of genes TPM>1.5
15500
16000
16500
17000
17500
18000
0
50
100
150
200
no. of individuals

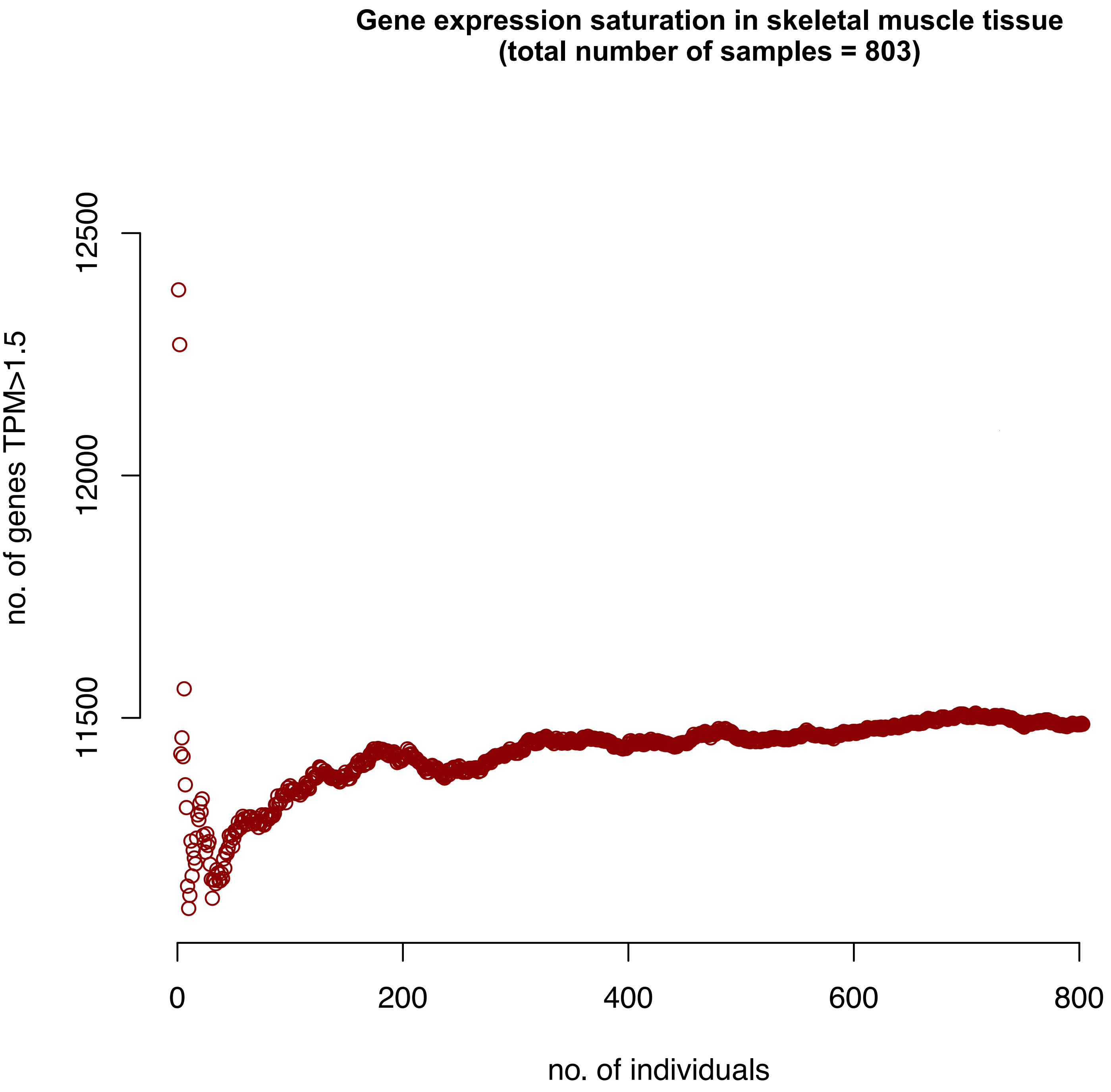
Gene expression saturation in skeletal muscle tissue
(total number of samples = 803)
no. of genes TPM>1.5
12500
12000
11500
0
200
400
600
800
no. of individuals

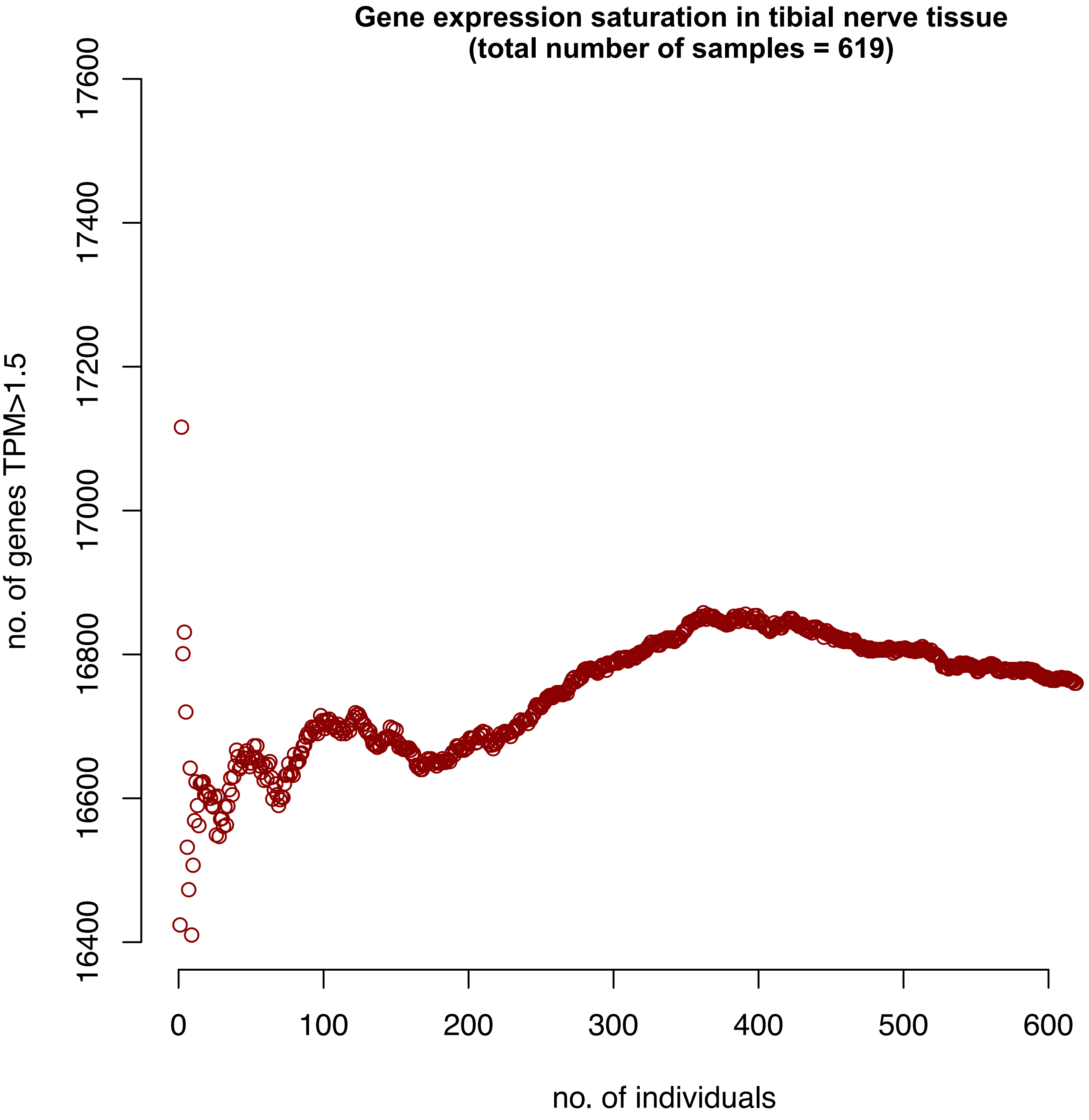
Gene expression saturation in tibial nerve tissue
(total number of samples = 619)
no. of genes TPM>1.5
16400
16600
16800
17000
17200
17400
17600
0
100
200
300
400
500
600
no. of individuals

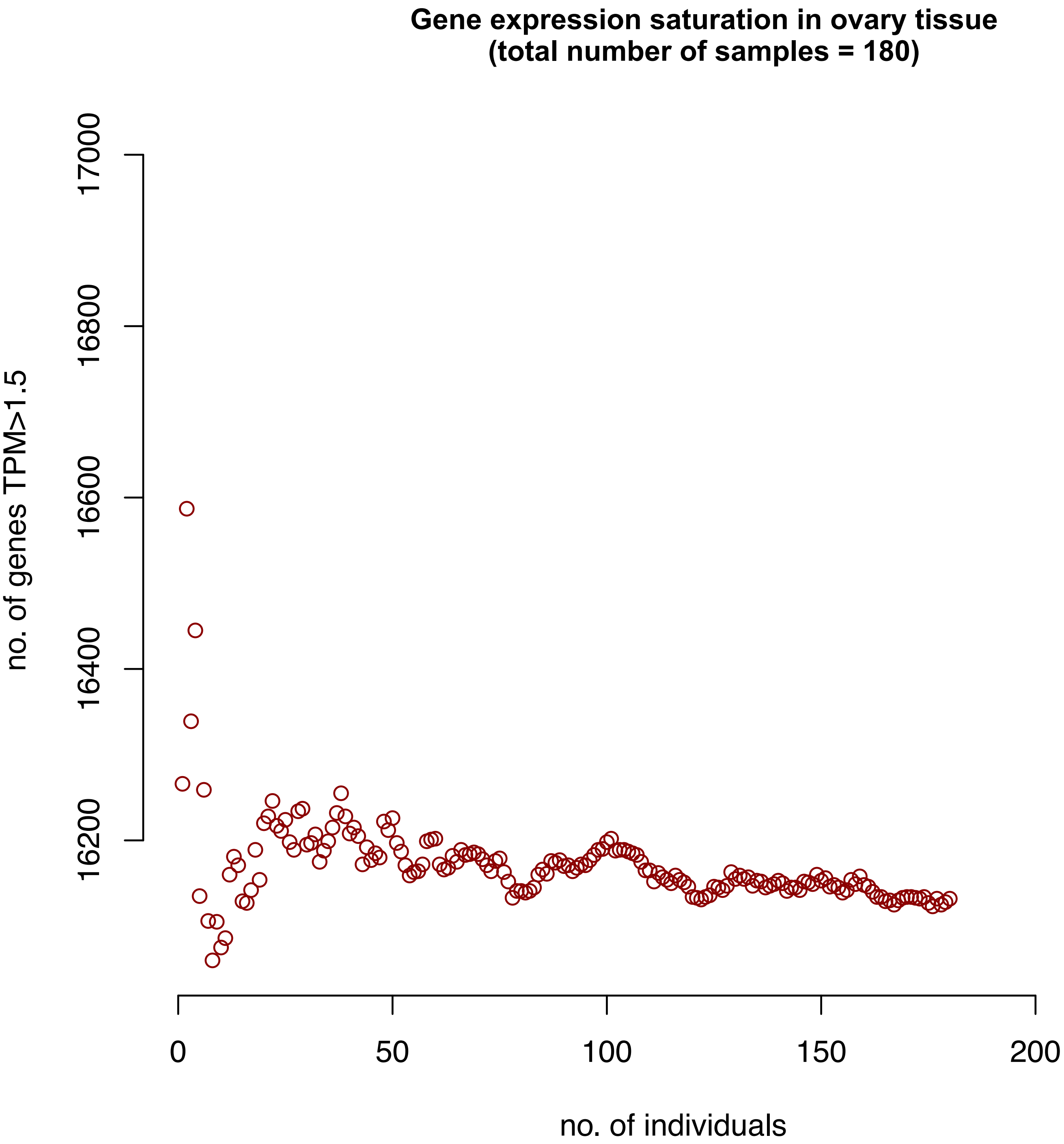
Gene expression saturation in ovary tissue
(total number of samples = 180)
no. of genes TPM>1.5
16200
16400
16600
16800
17000
0
50
100
150
200
no. of individuals

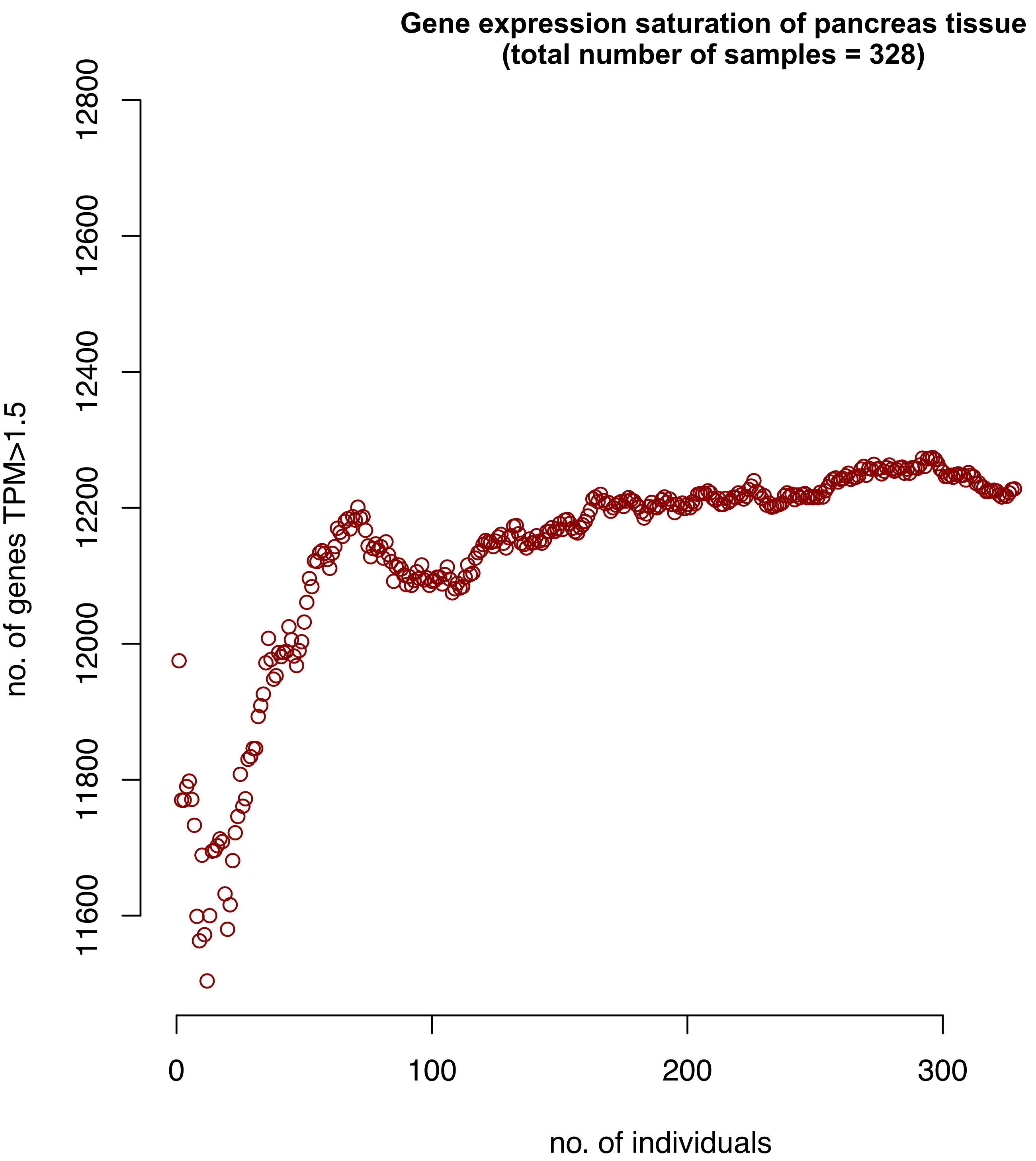

Gene expression saturation of pancreas tissue
(total number of samples = 328)
no. of genes TPM>1.5
11600
11800
12000
12200
12400
12600
12800
0
100
200
300
no. of individuals

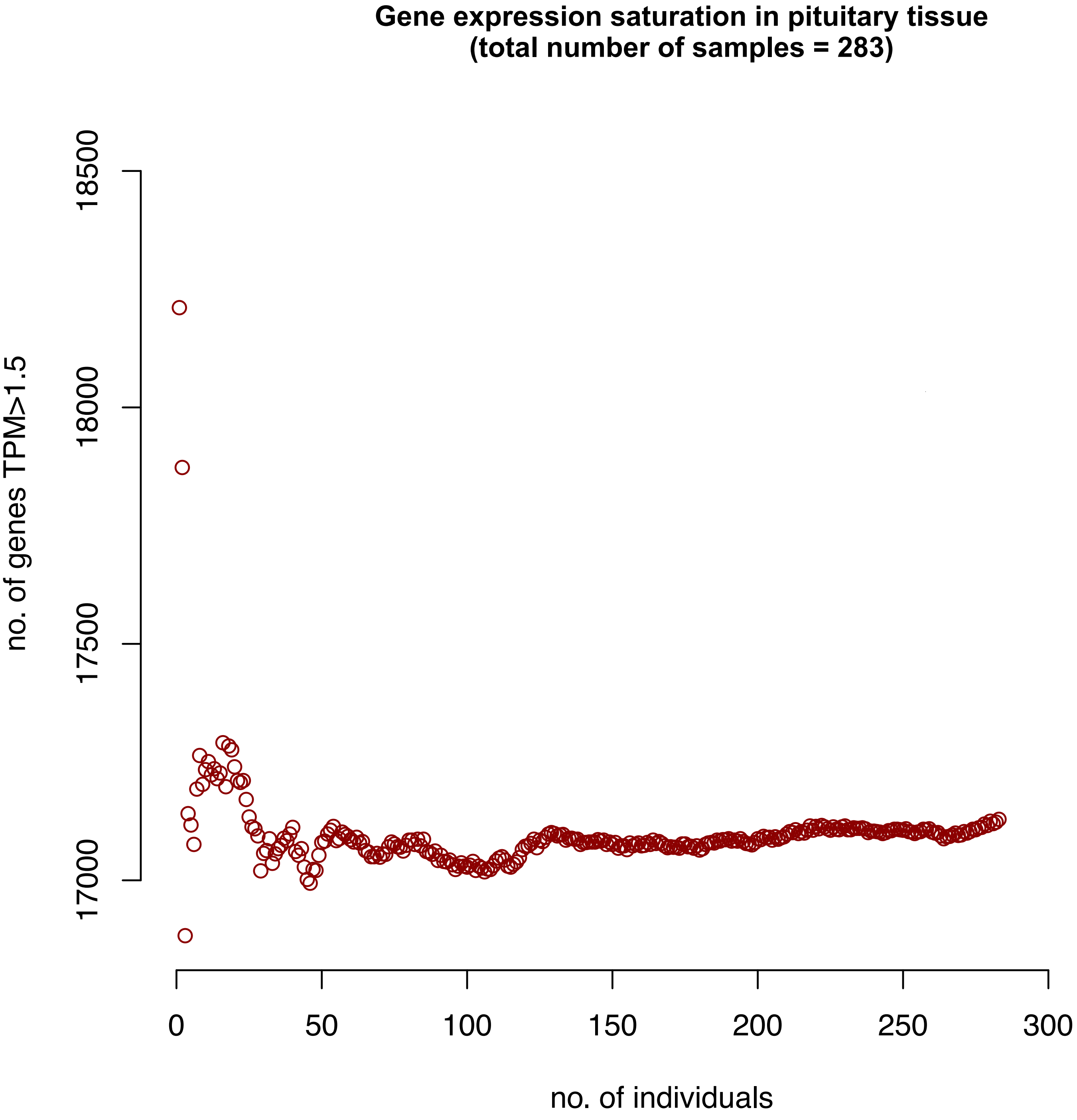
Gene expression saturation in pituitary tissue
(total number of samples = 283)
no. of genes TPM>1.5
17000
17500
18000
18500
0
50
100
150
200
250
300
no. of individuals

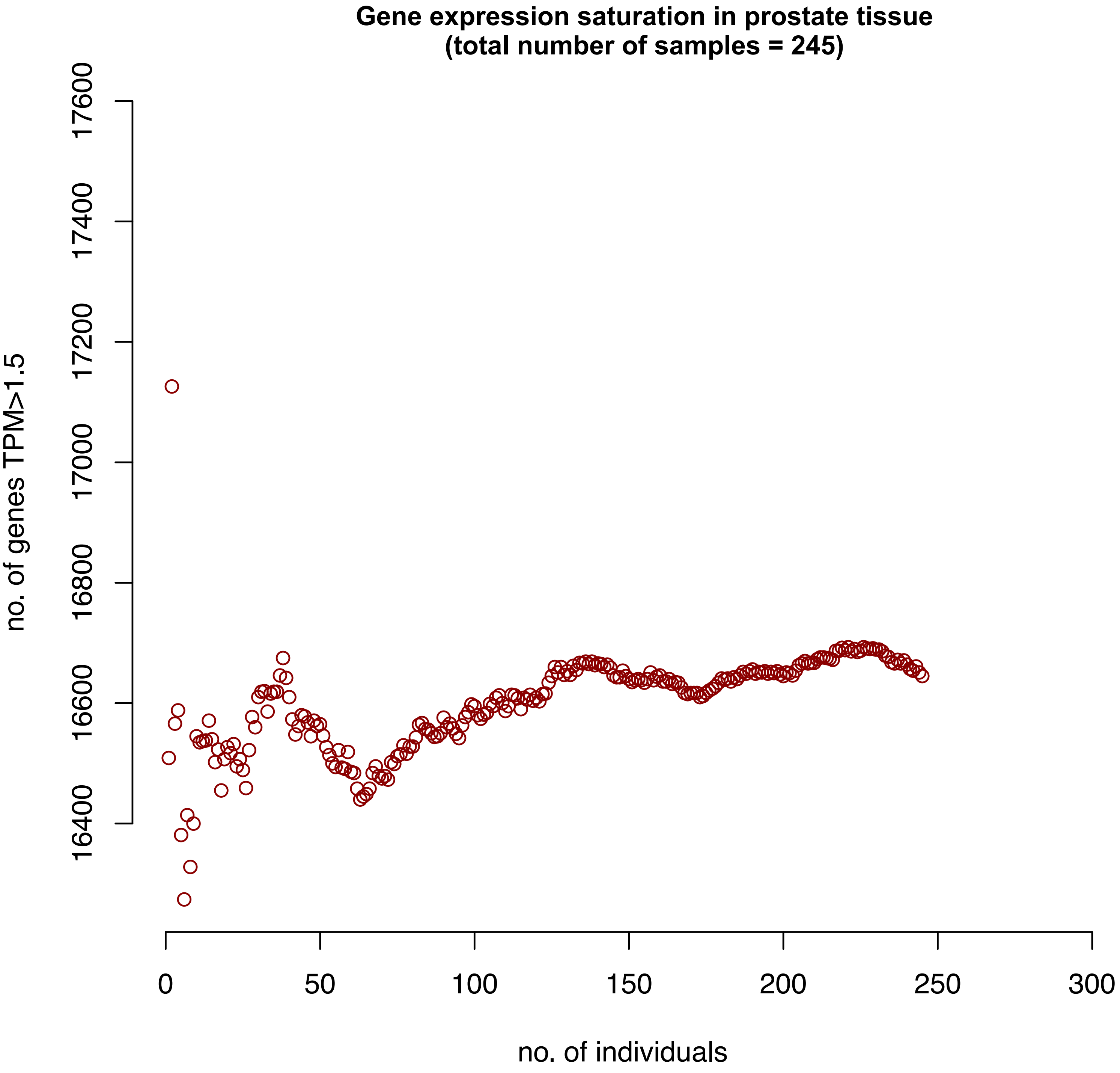
Gene expression saturation in prostate tissue
(total number of samples = 245)
no. of genes TPM>1.5
16400
16600
16800
17000
17200
17400
17600
0
50
100
150
200
250
300
no. of individuals

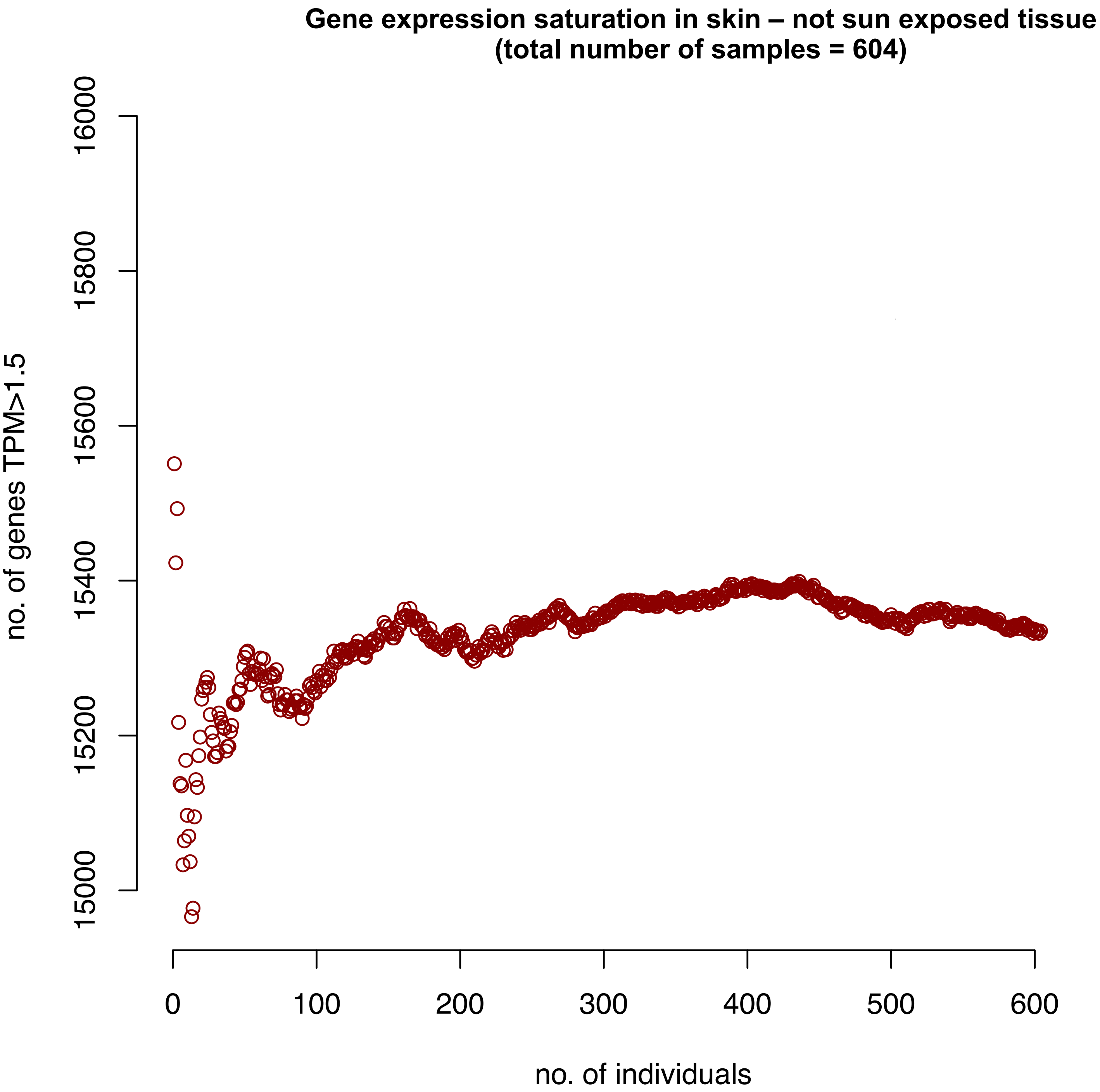
Gene expression saturation in skin – not sun exposed tissue
(total number of samples = 604)
no. of genes TPM>1.5
16000
15800
15600
15400
15200
15000
0
100
200
300
400
500
600
no. of individuals

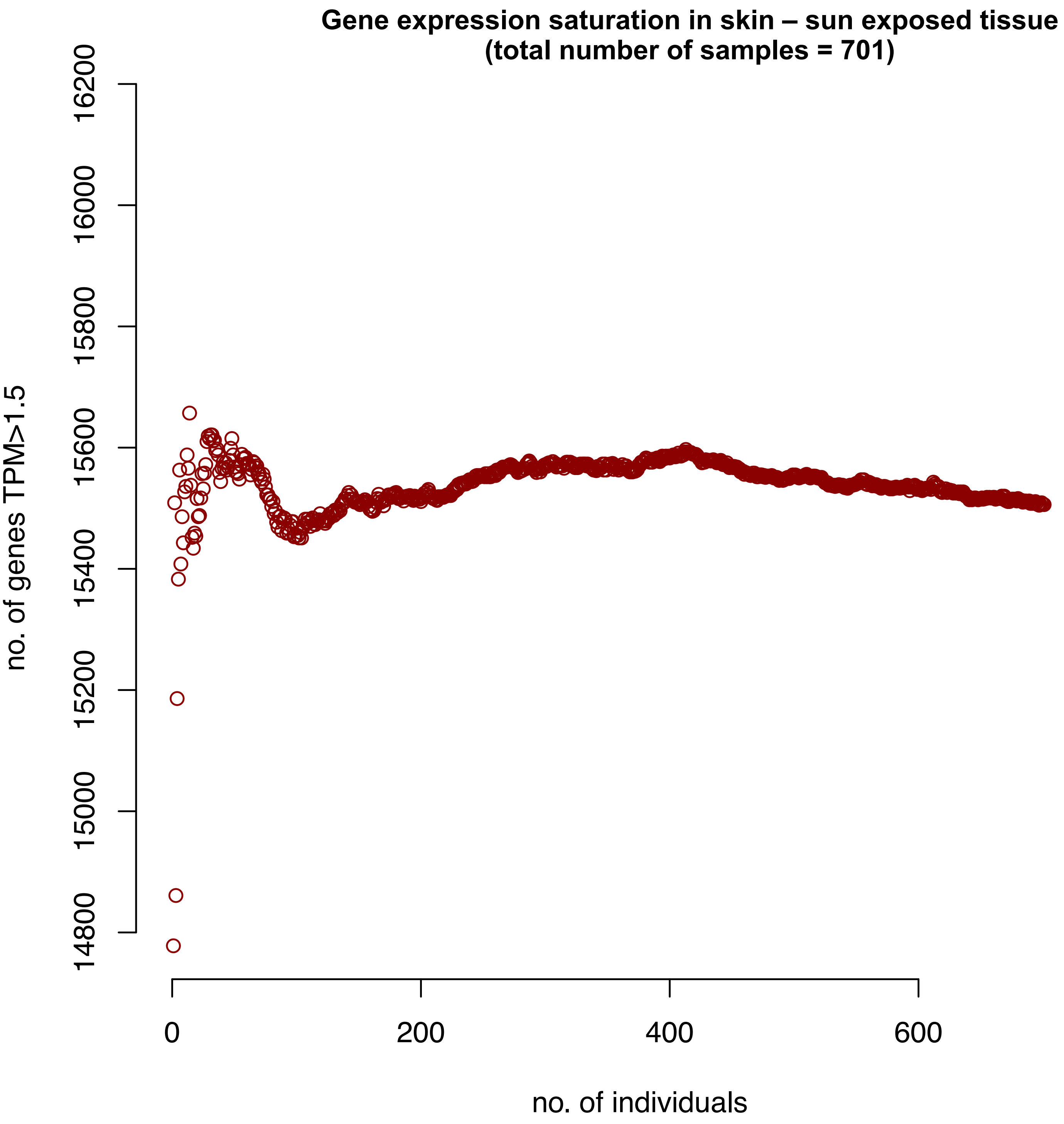
Gene expression saturation in skin – sun exposed tissue
(total number of samples = 701)
no. of genes TPM>1.5
14800
15000
15200
15400
15600
15800
16000
16200
0
200
400
600
no. of individuals

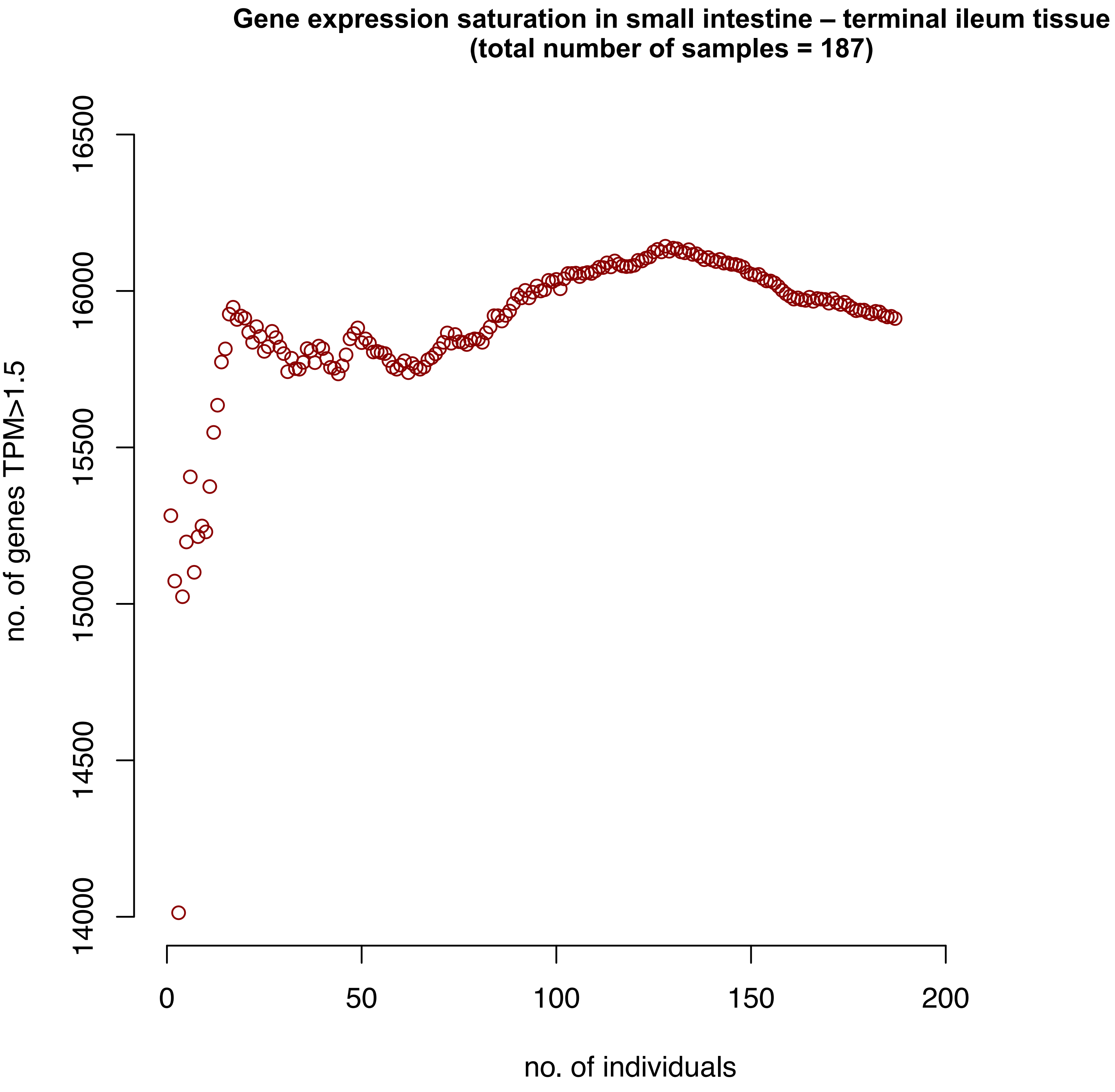
Gene expression saturation in small intestine – terminal ileum tissue
(total number of samples = 187)
no. of genes TPM>1.5
14000
14500
15000
15500
16000
16500
0
50
100
150
200
no. of individuals

Gene expression saturation in spleen tissue
(total number of samples = 241)

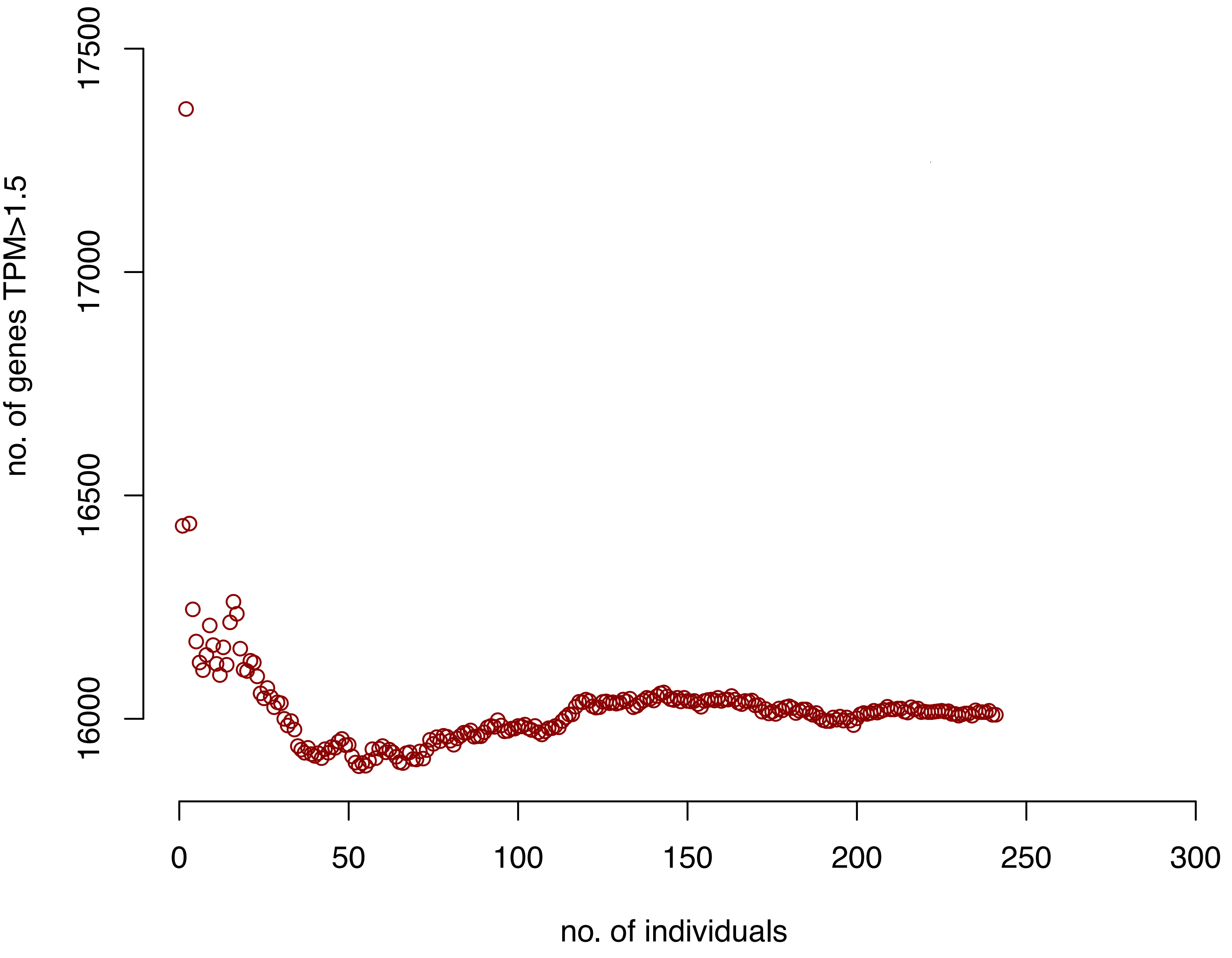

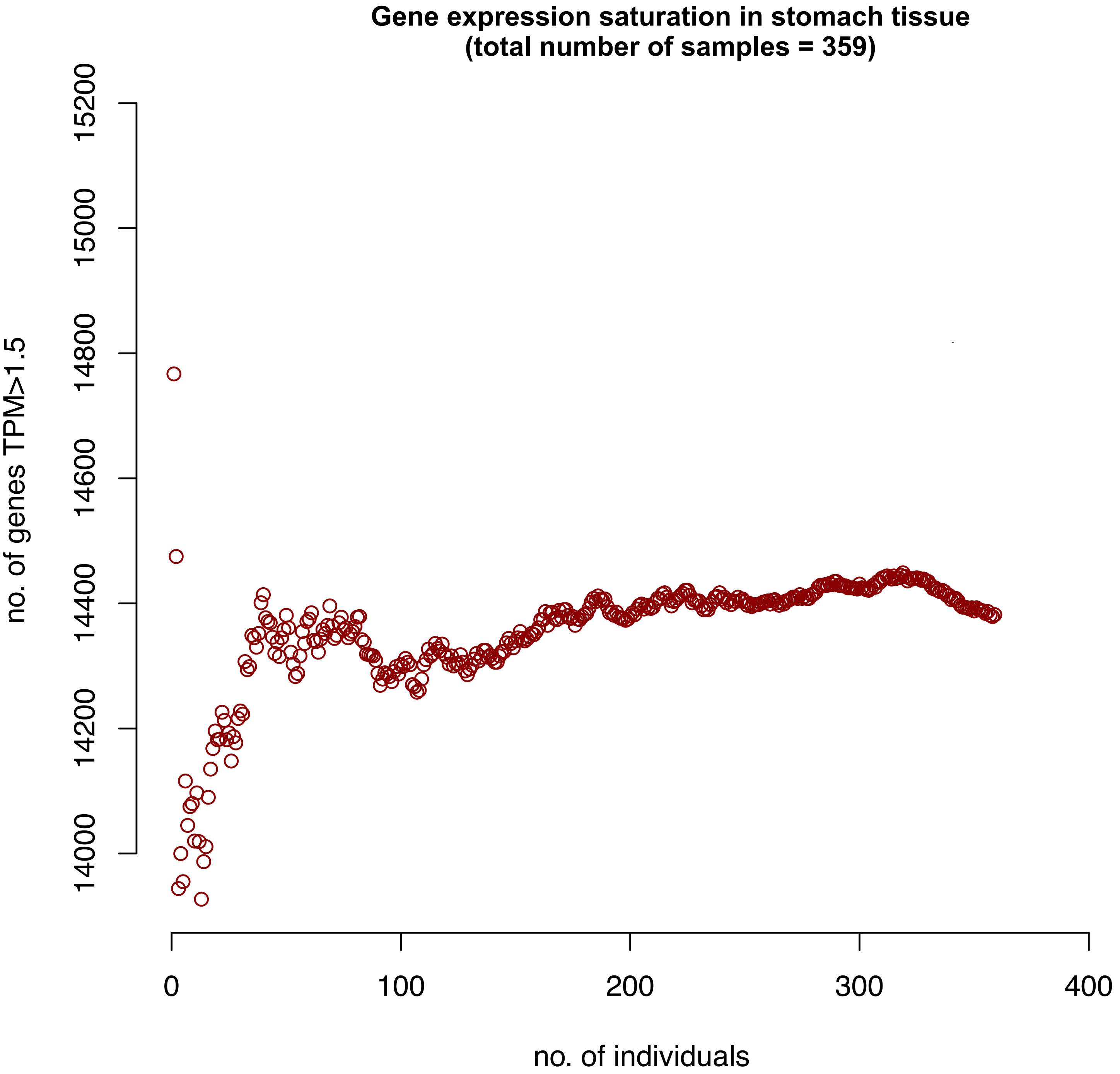
Gene expression saturation in stomach tissue
(total number of samples = 359)
no. of genes TPM>1.5
15200
15000
14800
14600
14400
14200
14000
0
100
200
300
400
no. of individuals

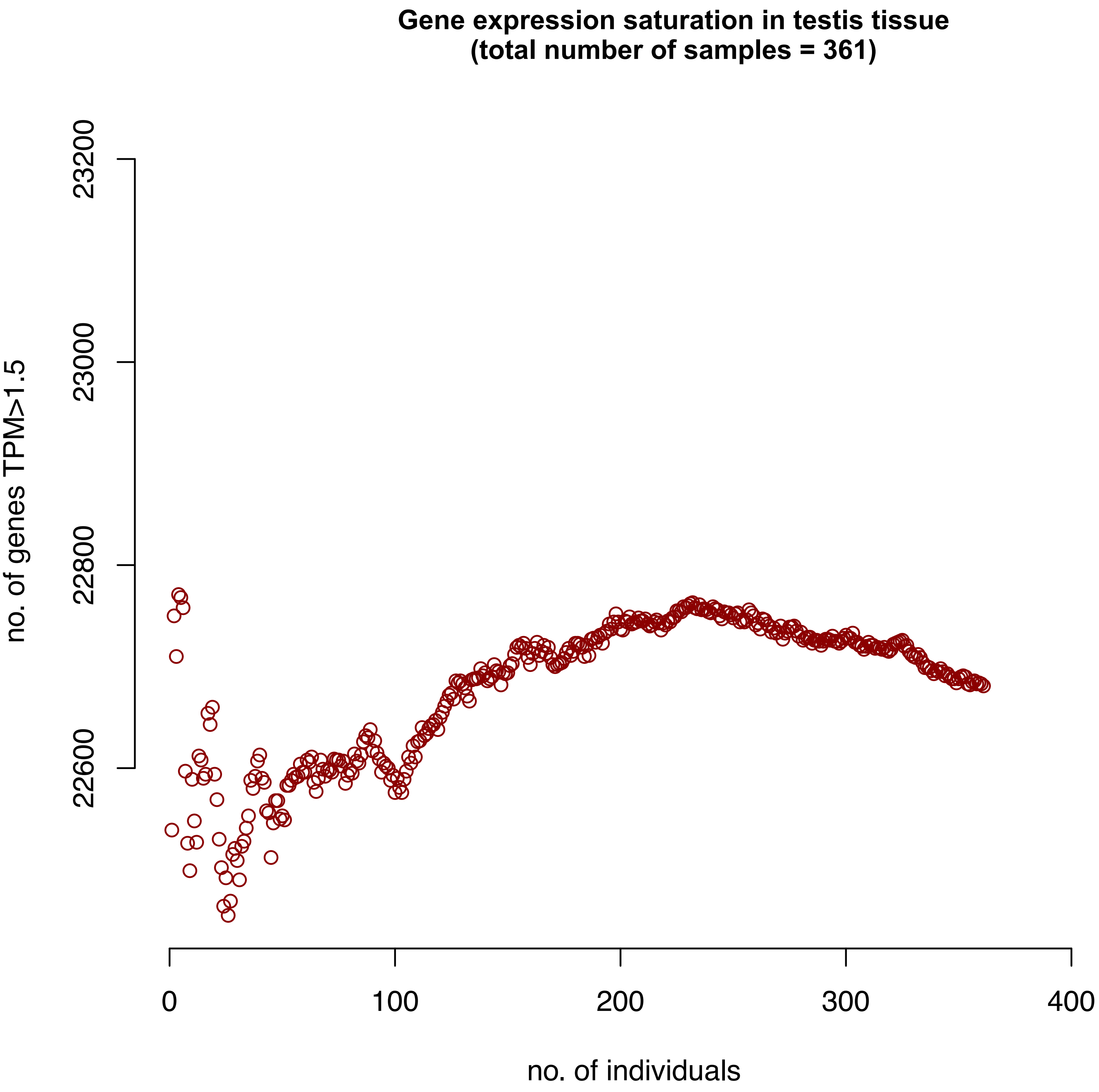

Gene expression saturation in testis tissue
(total number of samples = 361)
no. of genes TPM>1.5
23200
23000
22800
22600
0
100
200
300
400
no. of individuals

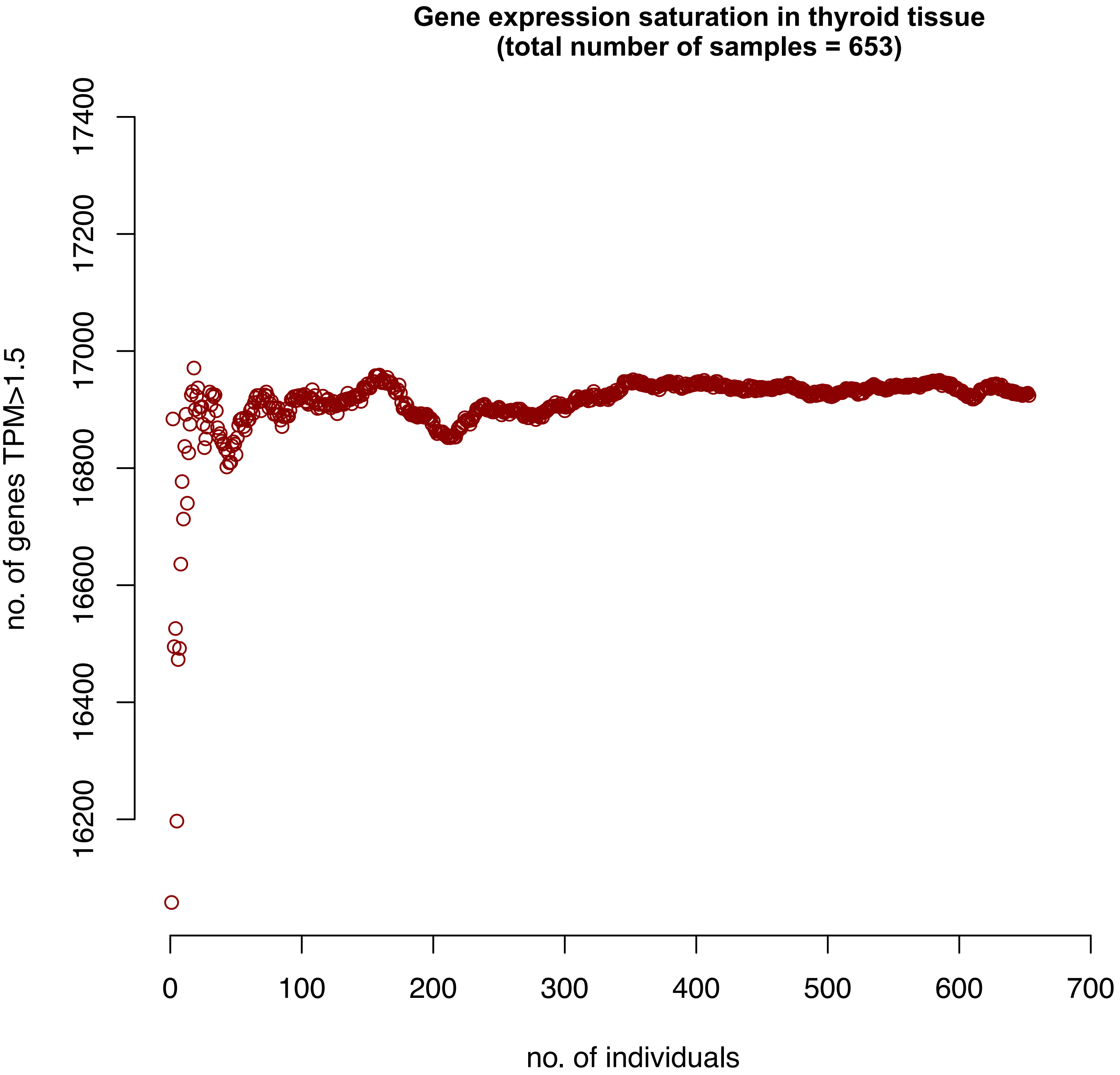
Gene expression saturation in thyroid tissue
(total number of samples = 653)
no. of genes TPM>1.5
16200
16400
16600
16800
17000
17200
17400
0
100
200
300
400
500
600
700
no. of individuals

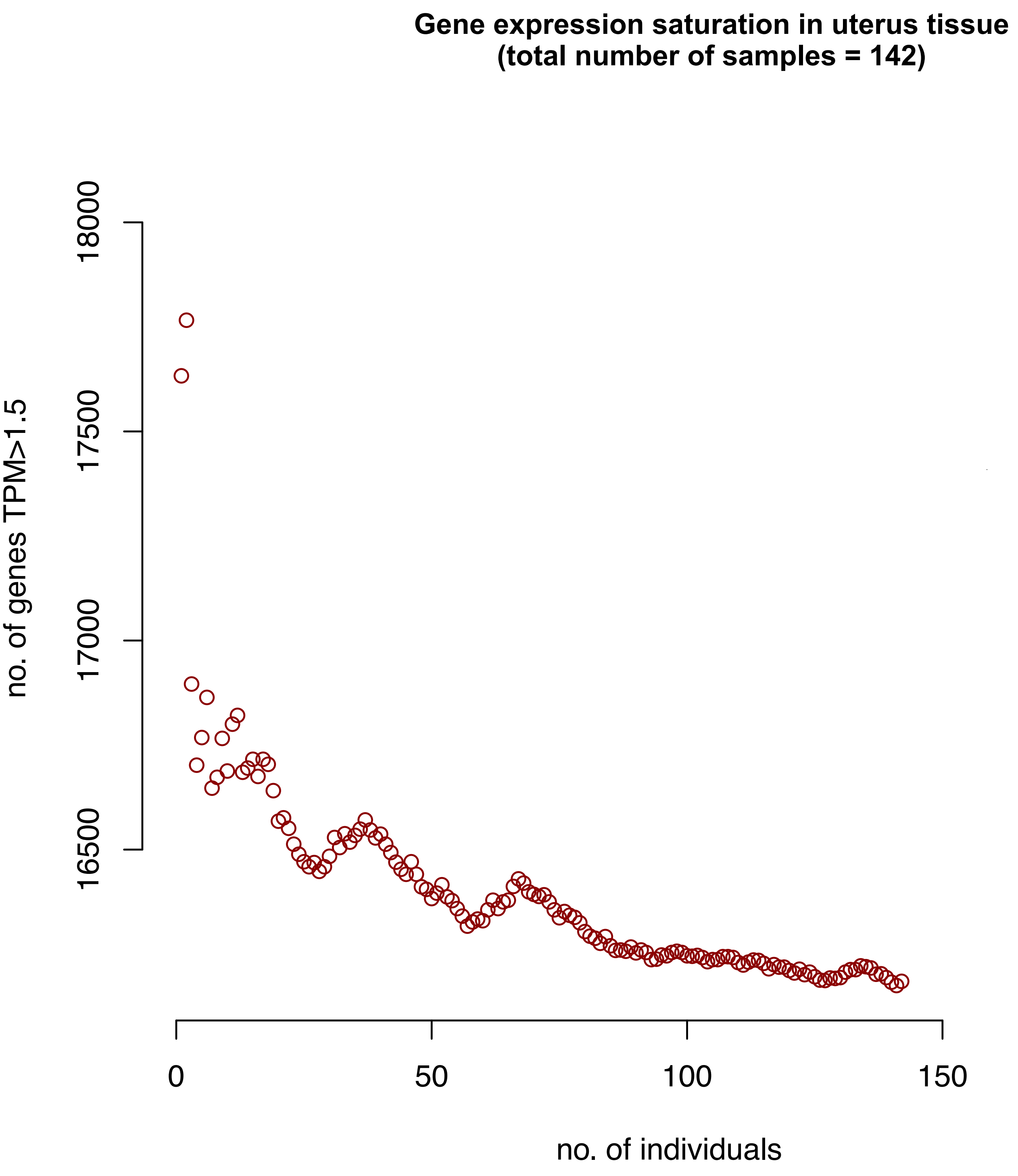
Gene expression saturation in uterus tissue
(total number of samples = 142)
no. of genes TPM>1.5
18000
17500
17000
16500
0
50
100
150
no. of individuals

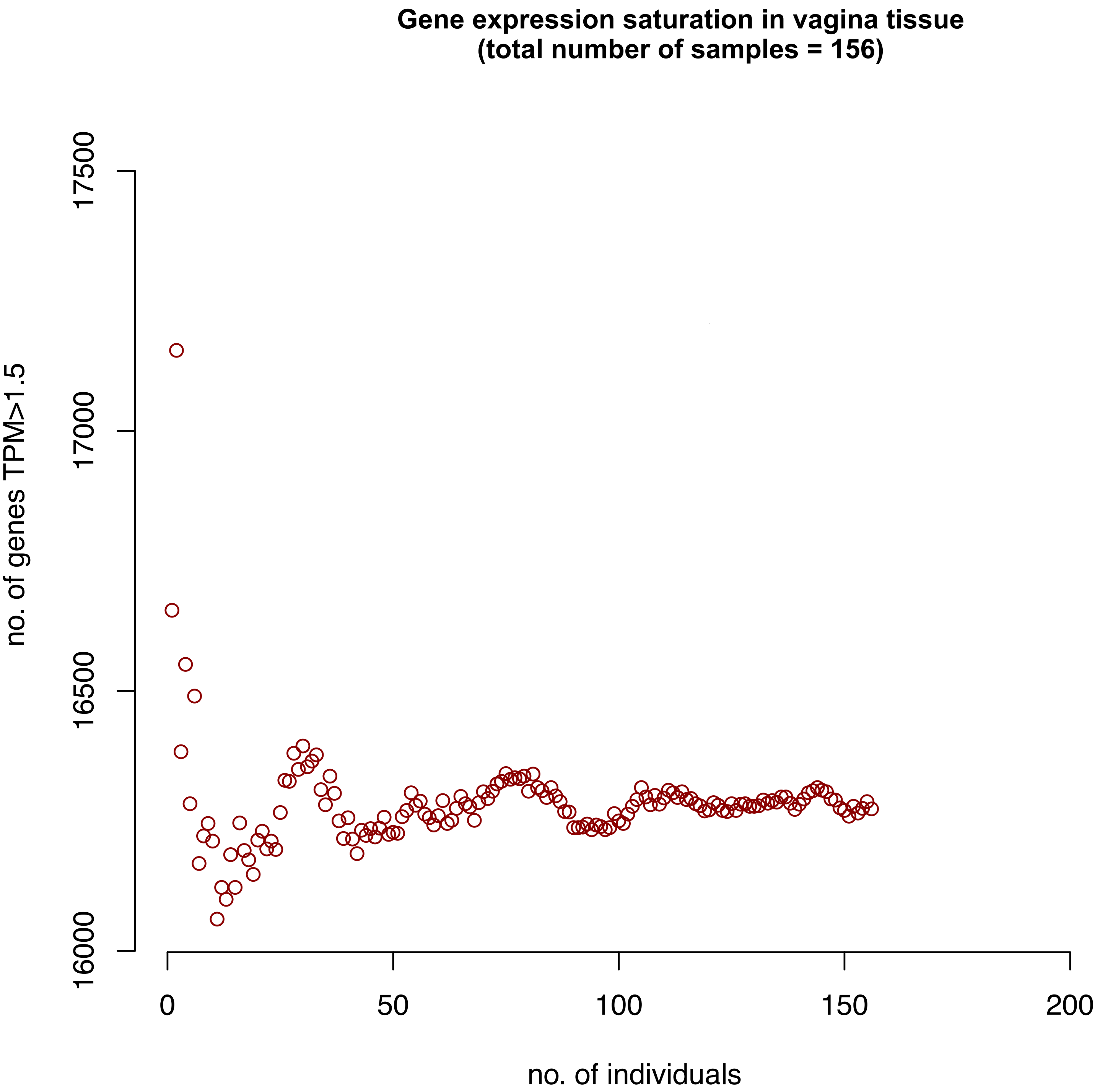
Gene expression saturation in vagina tissue
(total number of samples = 156)
no. of genes TPM>1.5
17500
17000
16500
16000
0
50
100
150
200
no. of individuals

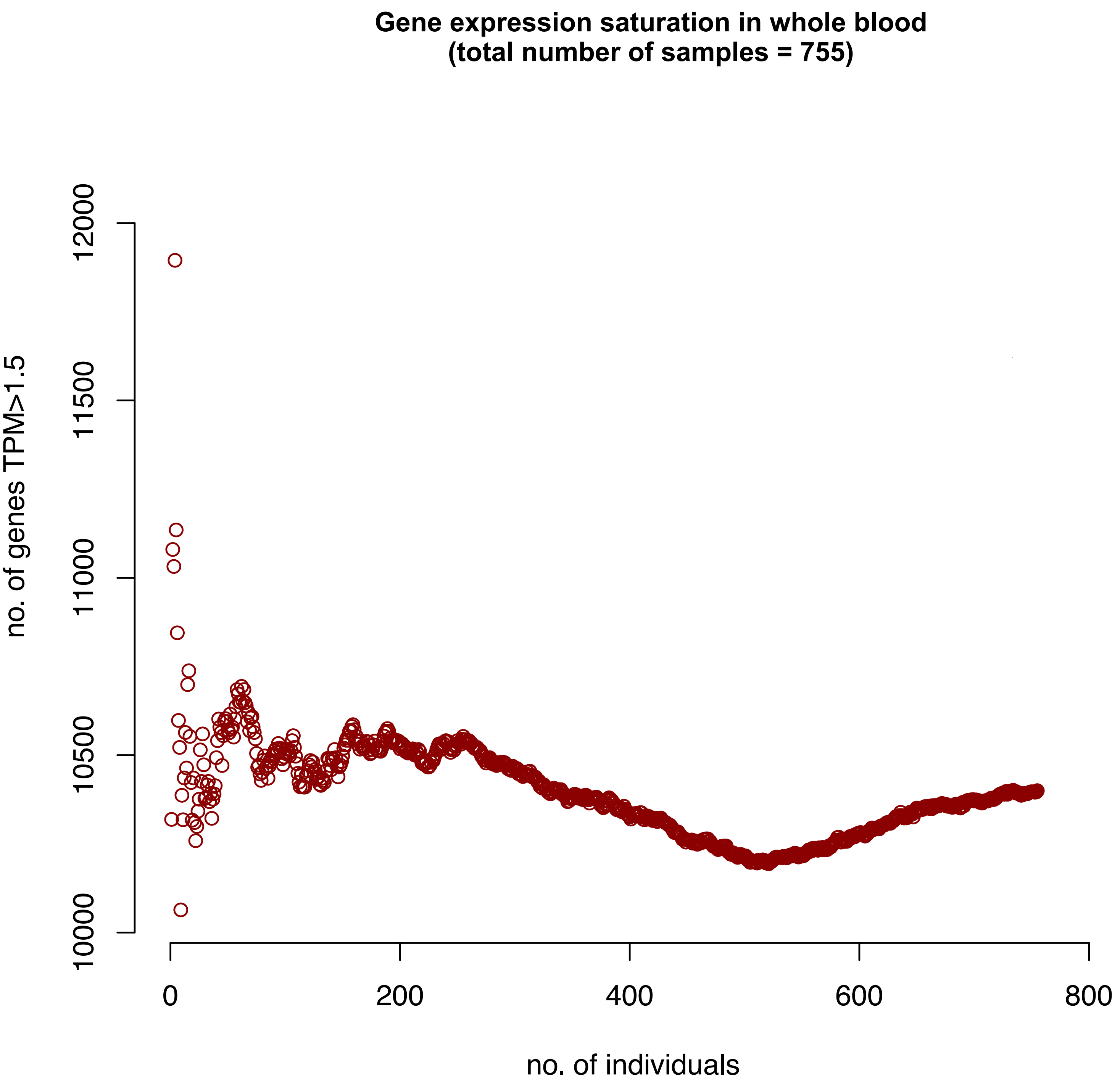
Gene expression saturation in whole blood
(total number of samples = 755)
no. of genes TPM>1.5
12000
11500
11000
10500
10000
0
200
400
600
800
no. of individuals
