# Supplementary figures and images for: Phenotype-tissue expression and exploration (PTEE) resource facilitates the choice of tissue for RNA-seq-based clinical genetics studies
Source: BMC Genomics. 2021 Nov 7;22:802. doi: 10.1186/s12864-021-08125-9 (PMC8573933; doi:10.1186/s12864-021-08125-9)

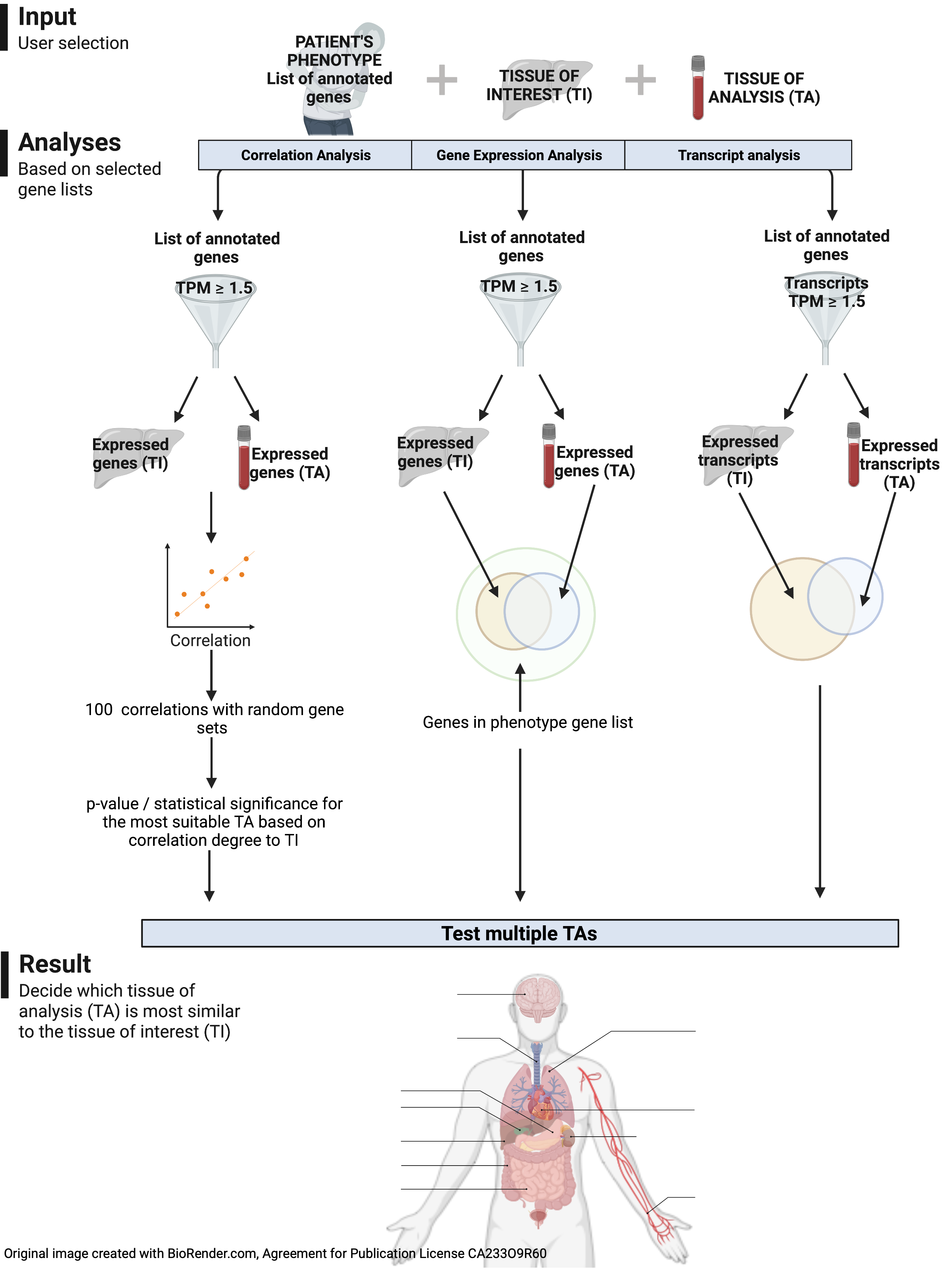

Supplement: Supplementary file 2 — Additional file 2: Supplementary Material – File 2: Scheme of PTEE workflow. (Original image created with BioRender.com, Agreement for Publication License CA233O9R60) [file 12864_2021_8125_MOESM2_ESM.png]
