# Supplementary material for: Phenotype-tissue expression and exploration (PTEE) resource facilitates the choice of tissue for RNA-seq-based clinical genetics studies
Source: BMC Genomics. 2021 Nov 7;22:802. doi: 10.1186/s12864-021-08125-9 (PMC8573933; doi:10.1186/s12864-021-08125-9)
Supplement: Supplementary file 3 — Additional file 3: Supplementary Material – File 3: Instructions for using PTEE. [file 12864_2021_8125_MOESM3_ESM.pdf]

Phenotype-Tissue Expression and Exploration (PTEE) resource informs the tissue choice for RNA-seq-based clinical genetics studies

Akhil Velluva[1,2], Maximillian Radtke[3], Susanne Horn [2], Bernt Popp [3], Konrad Pltazer [3], Erind Gjermeni [4,5], Chen-Ching Lin [6], Johannes R. Lemke [3], Antje Garten [7], Torsten Schöneberg [2], Rami Abou Jamra [3], Diana Le Duc [1,3]

Supplementary File 3

Instructions for the online tool usage.

For bugs reports or error messages please contact Akhil Velluva at akhilvbioinfo@gmail.com.

Phenotype-Tissue Expression & Exploration

See page 3 | See page 4 | See page 5 | See page 6

Choose Phenotype | Correlation Analysis | Expression Analysis | Transcript Analysis | Single Gene Analysis | About

Select Your Phenotype of Interest

Cardiac Arrythmia

Choose a phenotype based on the patient´s symptoms

Tissue of Interest

Heart Atrial Appendage Heart Left Ventricle

Choose one or more tissues of interest, based on the inferred pathophysiology

Tissue of Analysis

Whole Blood Muscle Skeletal

Choose one or more tissue of analysis

Reset

Deselect TI and TA

Custom Gene List

Upload gene list (see page 2)

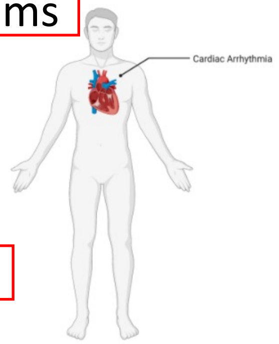

Show 10 entries

Search:

Data Source: GTEx Analysis Release V8
Displayed numbers represent median gene-level TPM by tissue

Gene ID	Ensembl ID	Whole Blood	Muscle Skeletal	Heart Atrial Appendage	Heart Left Ventricle
KCND3	ENSG00000171385.9	0.07	1.47	7.78	5.28
CASQ2	ENSG00000118729.11	0.12	126.63	416.66	439.09
RYR2	ENSG00000198626.15	0.04	0.09	75.78	82.30
CALM2	ENSG00000143933.16	234.45	131.85	233.71	205.41
CAV3	ENSG00000182533.6	0.00	46.48	10.39	13.03
GPD1L	ENSG00000152642.10	2.63	101.87	73.78	62.77
SCN5A	ENSG00000183873.15	0.11	0.47	36.13	35.11
SCN10A	ENSG00000185313.6	0.00	0.00	0.07	0.04
SLMAP	ENSG00000163681.14	2.60	12.16	17.80	13.74
FGF12	ENSG00000114279.13	0.01	0.19	25.43	18.29

Showing 1 to 10 of 39 entries

Previous 1 2 3 4 Next

Download displayed TPM table in CSV format

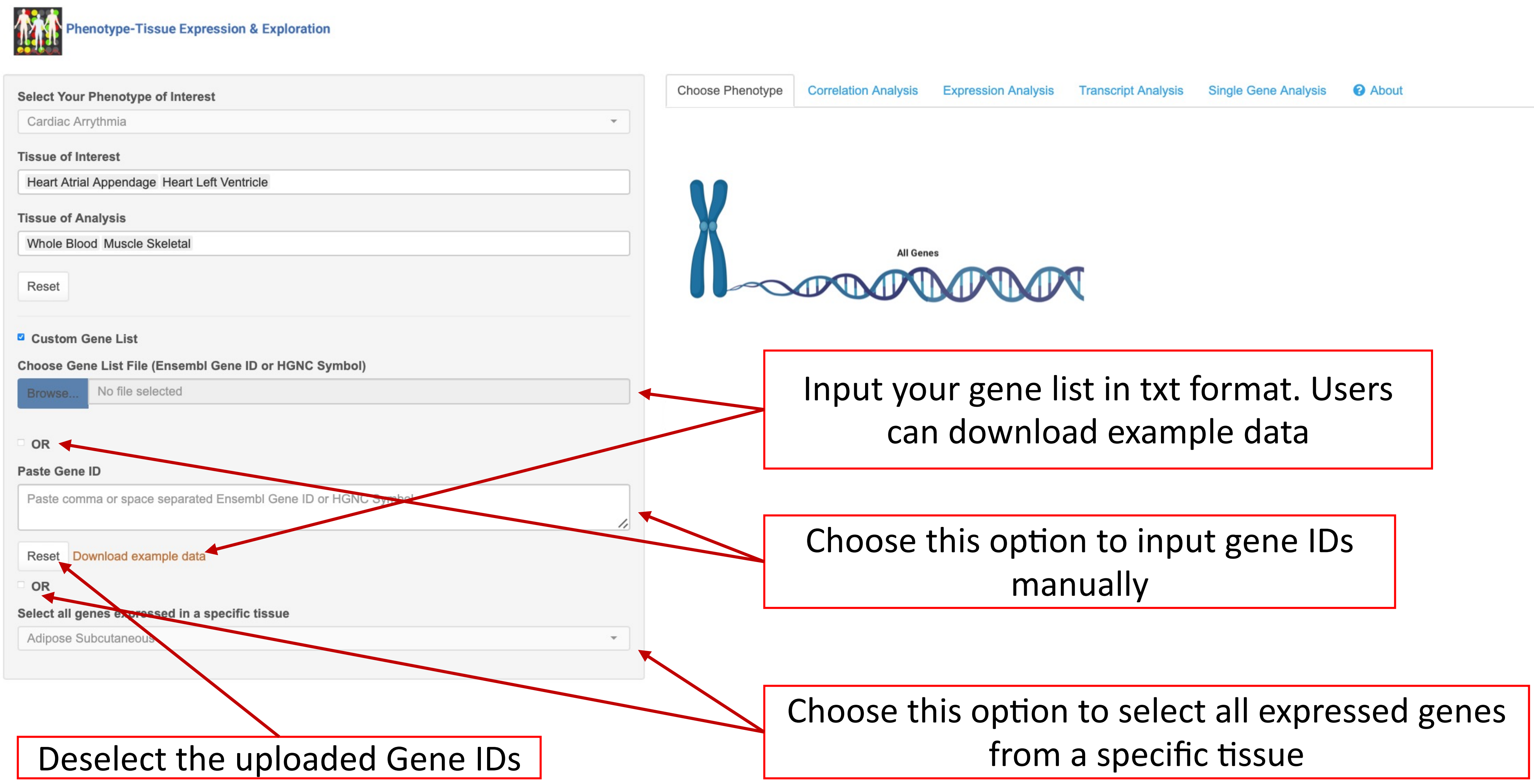
Phenotype-Tissue Expression & Exploration
Select Your Phenotype of Interest
Cardiac Arrythmia
Tissue of Interest
Heart Atrial Appendage
Heart Left Ventricle
Tissue of Analysis
Whole Blood
Muscle Skeletal
Reset
Custom Gene List
Choose Gene List File (Ensembl Gene ID or HGNC Symbol)
Browse...
No file selected
OR
Paste Gene ID
Reset
Download example data
OR
Select all genes expressed in a specific tissue
Adipose Subcutaneous
Choose Phenotype
Correlation Analysis
Expression Analysis
Transcript Analysis
Single Gene Analysis
About
All Genes
Input your gene list in txt format. Users can download example data
Choose this option to input gene IDs manually
Choose this option to select all expressed genes from a specific tissue
Deselect the uploaded Gene IDs

Phenotype-Tissue Expression & Exploration

Correlation-based on the expression levels of genes with the median TPM > 1.5 included in the list of the chosen phenotype/custom list

Tissue of Interest (TI)

Heart Atrial Appendage, Heart Left Ventricle

Tissue of Analysis (TA)

Whole Blood, Muscle Skeletal

Select file type for the plot

- (●) png
- ( ) pdf

Select the download file format for the plots

Choose Phenotype | Correlation Analysis | Expression Analysis | Transcript Analysis | Single Gene Analysis | About

Show 10 entries — Search:

Correlation matrix

	Heart Atrial Appendage	Heart Left Ventricle	Whole Blood	Muscle Skeletal
Heart Atrial Appendage	1	0.99371328515834	0.430413738492218	0.47683899669258
Heart Left Ventricle	0.99371328515834	1	0.365229139614015	0.434116354710954
Whole Blood	0.430413738492218	0.365229139614015	1	0.339340502162458
Muscle Skeletal	0.47683899669258	0.434116354710954	0.339340502162458	1

Showing 1 to 4 of 4 entries — Previous 1 Next

Download correlation coefficients table in CSV format

Pearson correlation coefficients

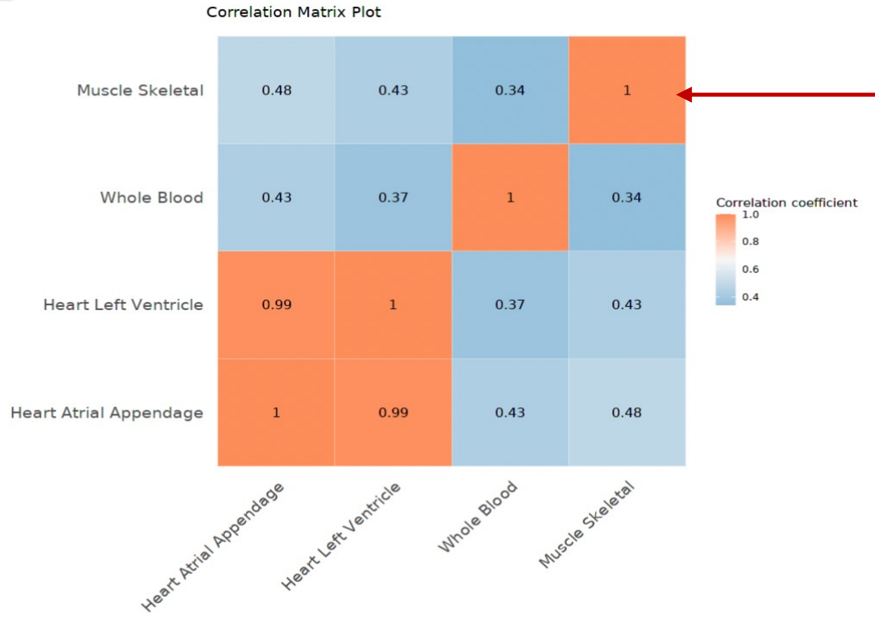

Download heat map plot

☑ Calculate P-Value from the Exact Binomial Test

Select this button if the user needs to find the p-value from the exact binomial test in 100 random iterations

Show 10 entries — Search:

Tissue of Analysis	P-Value	No. of highest correlations in 100 random iterations
Muscle Skeletal	6.11023682419108e-78	90
Whole Blood	0.548709834557997	10

Showing 1 to 2 of 2 entries — Previous 1 Next

Overlap of genes included in the inquired gene list (PI) and genes expressed (Median >1.5) in TA and TI

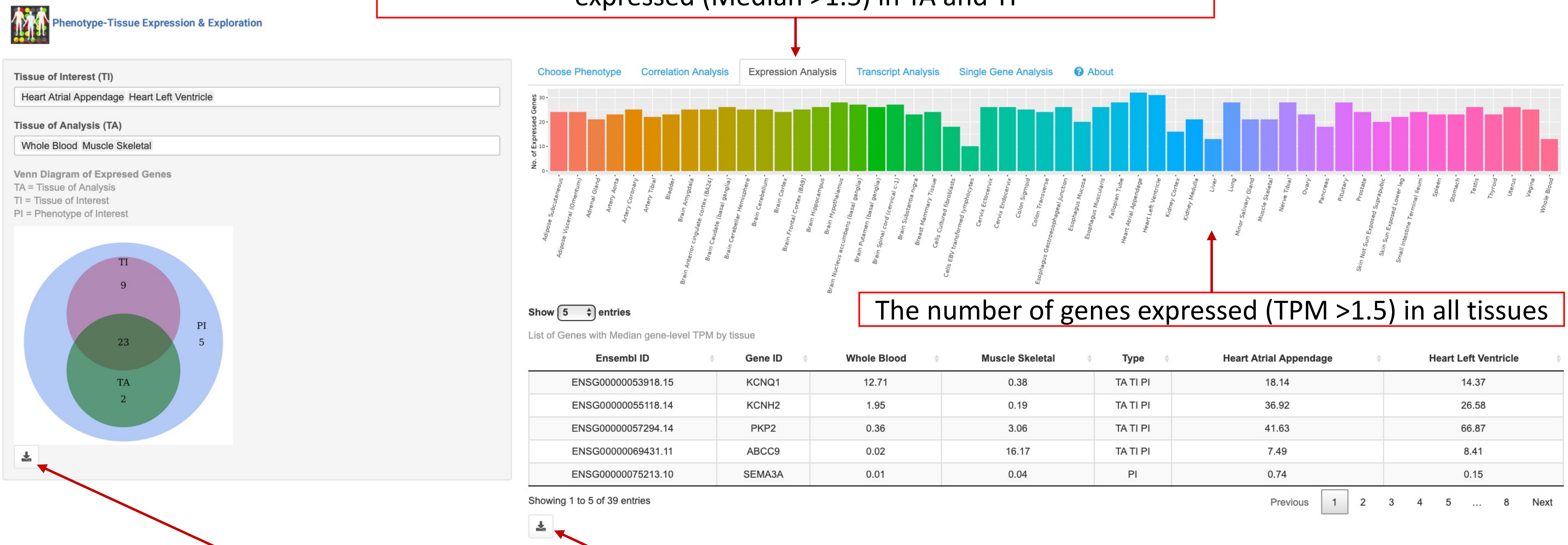

Ensembl ID	Gene ID	Whole Blood	Muscle Skeletal	Type	Heart Atrial Appendage	Heart Left Ventricle
ENSG00000053918.15	KCNQ1	12.71	0.38	TA TI PI	18.14	14.37
ENSG00000055118.14	KCNH2	1.95	0.19	TA TI PI	36.92	26.58
ENSG00000057294.14	PKP2	0.36	3.06	TA TI PI	41.63	66.87
ENSG00000069431.11	ABCC9	0.02	16.17	TA TI PI	7.49	8.41
ENSG00000075213.10	SEMA3A	0.01	0.04	PI	0.74	0.15

Download Venn diagram (PNG format)

Download median TPM values in CSV format

Overlap of transcripts included in the inquired gene list (PI) and transcripts expressed (Median >1.5) in TA and TI

Phenotype-Tissue Expression & Exploration

Tissue of Interest (TI)

Heart Atrial Appendage Heart Left Ventricle

Tissue of Analysis (TA)

Whole Blood Muscle Skeletal

Venn Diagram of Expresed Transcripts

TA = Tissue of Analysis

TI = Tissue of Interest

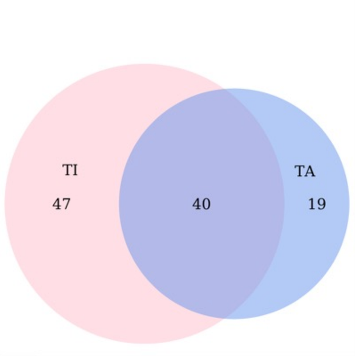

Download Venn diagram (PNG format)

Choose Phenotype | Correlation Analysis | Expression Analysis | Transcript Analysis | Single Gene Analysis | About

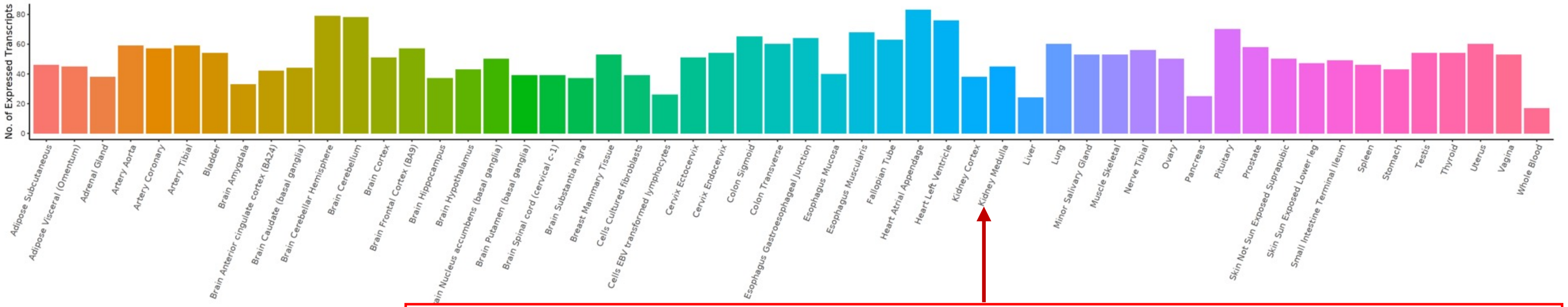

The number of transcripts expressed (TPM >1.5) in all tissues

Show 5 entries

List of Transcripts with Median Transcripts-level TPM by tissue

Gene ID	Ensembl Gene ID	Ensembl Transcript ID	Whole Blood	Muscle Skeletal	Type	Heart Atrial Appendage	Heart Left Ventricle
ABCC9	ENSG00000069431.11	ENST00000261200.8	0.00	9.38	TA TI	5.06	5.635
ABCC9	ENSG00000069431.11	ENST00000326684.8	0.00	2.18	TA	0.00	0.000
ABCC9	ENSG00000069431.11	ENST00000544039.5	0.00	3.50	TA	0.00	0.000
AKAP9	ENSG00000127914.16	ENST00000356239.7	0.00	2.11	TA	0.00	0.000
AKAP9	ENSG00000127914.16	ENST00000394534.6	0.00	4.79	TA TI	2.09	1.710

Showing 1 to 5 of 106 entries

Previous 1 2 3 4 5 … 22 Next

Download median TPM values in CSV format

Phenotype-Tissue Expression & Exploration

Explore expression of particular genes

Choose Phenotype | Correlation Analysis | Expression Analysis | Transcript Analysis | Single Gene Analysis | About

Enter Gene ID

CASQ2 RYR2

Select Tissue

Adipose Subcutaneous Adipose Visceral (Omentum)

Select the gene of interest included in the initial list (See choose phenotype)

Select the tissue to inquire gene expression

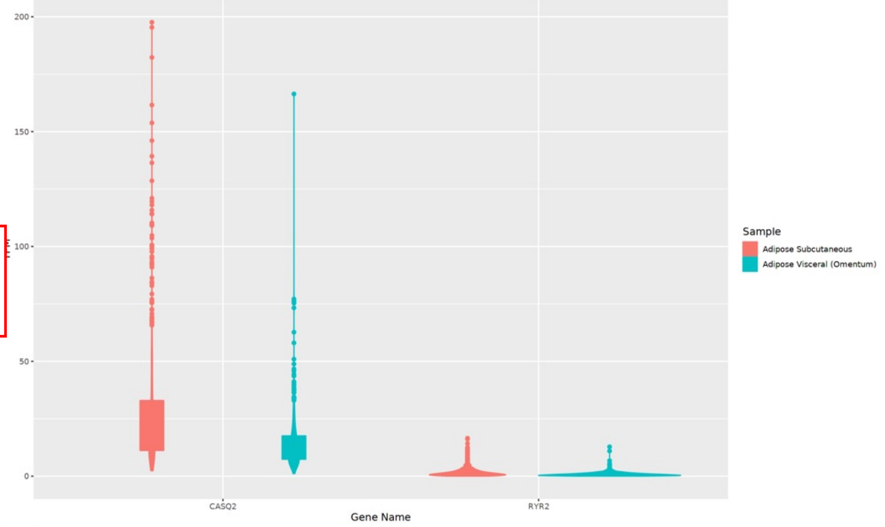

Download violin plot

Show 10 **entries**

Search:

Data Source: GTEx Analysis Release V8
Displayed numbers represent gene-level TPM by tissue

Gene Name	Tissue	TPM
CASQ2	Adipose Subcutaneous	8.541
CASQ2	Adipose Subcutaneous	9.931
CASQ2	Adipose Subcutaneous	55.48
CASQ2	Adipose Subcutaneous	7.459
CASQ2	Adipose Subcutaneous	9.511
CASQ2	Adipose Subcutaneous	15.79
CASQ2	Adipose Subcutaneous	9.744
CASQ2	Adipose Subcutaneous	27.02
CASQ2	Adipose Subcutaneous	9.767
CASQ2	Adipose Subcutaneous	128.6

Showing 1 to 10 of 2,408 entries

Previous 1 2 3 4 5 … 241 Next

Download TPM values in all samples from the selected tissue/tissues (CSV format)
